# Comprehensive Phytopharmacological Profiling of *Polygonum aviculare* L.: Antioxidant, Anti-Inflammatory, and α-Glucosidase Inhibitory Activities of Bioactive Constituents with Molecular Docking and In Silico Drug-Likeness Analysis

**DOI:** 10.3390/antiox15080947

**Published:** 2026-07-29

**Authors:** Yuen-Sing Lee, Sin-Min Li, Hui-Ju Chen, Jih-Jung Chen

**Affiliations:** 1 Biomedical Industry Ph.D. Program, College of Life Sciences, National Yang Ming Chiao Tung University, Taipei 112304, Taiwan; ysl.ls11@nycu.edu.tw; 2 Department of Pharmacy, School of Pharmaceutical Sciences, National Yang Ming Chiao Tung University, Taipei 112304, Taiwan; samuel147samuel147@gmail.com; 3 College of Management, Executive Master of Business Administration (EMBA), National Yang Ming Chiao Tung University, Taipei 112304, Taiwan; kay@healthmate.com.tw; 4 Department of Medical Research, China Medical University Hospital, China Medical University, Taichung 404333, Taiwan; 5 Traditional Herbal Medicine Research Center, Taipei Medical University Hospital, Taipei 110301, Taiwan

**Keywords:** *Polygonum aviculare*, anti-inflammation, traditional Chinese medicine, macrophage polarization, antioxidant, anti-α-glucosidase

## Abstract

This study investigated the extracts and bioactive constituents of the aerial parts of *Polygonum aviculare* L., a well-known traditional medicine for inflammation and diuresis, through bioactivity-guided investigation and molecular docking analysis. Solvent extracts demonstrated significant antioxidant, anti-α-glucosidase, and anti-inflammatory activities, supporting their value for further pharmacological investigation. Among the isolated compounds, gallic acid (**1**), quercetin (**3**), myricetin (**4**), avicularin (**6**), and isorhamnetin (**7**) exhibited excellent antioxidant activity in the DPPH assay (SC_50_ ≤ 6.50 μM) and were stronger than the positive control BHT (SC_50_ = 36.99 μM). Kaempferol (**9**) (IC_50_ of 4.50 µM) demonstrated the highest α-glucosidase inhibition, surpassing that of the clinically used acarbose (IC_50_ = 519.7 µM). Additionally, gallic acid and quercetin demonstrated significantly stronger NO inhibition than apigenin, while kaempferol showed comparable activity (IC_50_ = 15.06 ± 1.50 μM vs. 16.08 ± 1.86 μM for apigenin). Molecular docking analyses supported favorable interactions between the active compounds and α-glucosidase and inducible nitric oxide synthase (iNOS). Kaempferol reduced the expression of iNOS and COX-2 in LPS-stimulated RAW 264.7 macrophages and was associated with increased KLF4 and Arg-1 expression and decreased phosphorylation of p38 MAPK and IκBα, suggesting a potential role in promoting an anti-inflammatory M2-like macrophage phenotype. Structure–activity relationship analysis indicated that flavonoid aglycones and specific hydroxylation patterns contribute to the observed biological activities. Collectively, these findings identify quercetin and kaempferol as highly active constituents of *P. aviculare* and support their potential for further pharmacological investigation.

## 1. Introduction

Inflammatory disorders and oxidative stress-related pathologies represent significant global health burdens, contributing to chronic diseases such as diabetes, cardiovascular disorders, and cancer [1]. The World Health Organization (WHO) reports that non-communicable diseases (NCDs) are responsible for nearly three-quarters of global mortality, with cardiovascular diseases alone accounting for the majority of these fatalities [2]. Chronic inflammation drives tissue damage and promotes carcinogenesis, with oxidative stress exacerbating cellular dysfunction through reactive oxygen species (ROS)-mediated damage to proteins, lipids, and DNA [3]. Conventional treatments like NSAIDs and immunosuppressive agents frequently induce undesirable side effects, including gastrointestinal hemorrhage and heightened susceptibility to infections [4,5]. This safety concern has intensified the search for safer alternatives from natural sources, with plant-derived bioactive compounds emerging as promising candidates in modern drug discovery pipelines [6]. To leverage this potential, our research has focused on systematically isolating bioactive phytochemicals from natural sources, yielding compounds with validated anti-inflammatory, anticancer, and immunomodulatory properties [7,8,9,10,11,12,13,14,15].

Oxidative stress, chronic low-grade inflammation, and postprandial hyperglycemia are mechanistically interrelated hallmarks of metabolic syndrome and type 2 diabetes mellitus. Excessive accumulation of reactive oxygen species (ROS) and free radicals has been implicated in the pathogenesis of both conditions, contributing to pancreatic β-cell dysfunction, impaired insulin signaling, endothelial damage, and macrophage-mediated inflammatory responses [16,17]. In particular, free radical-induced oxidative damage to cellular macromolecules has been shown to activate pro-inflammatory NF-κB and MAPK signaling pathways, perpetuating a self-reinforcing cycle of tissue injury and metabolic dysregulation [18]. A plant constituent capable of attenuating free radical accumulation, suppressing macrophage-mediated inflammation, and inhibiting α-glucosidase activity would therefore represent a multi-target candidate of particular relevance to metabolic disease management. The present study was designed within this integrative framework to systematically evaluate whether the phytochemical constituents of *P. aviculare* aerial parts address these three interrelated targets concurrently.

**Figure 1:**
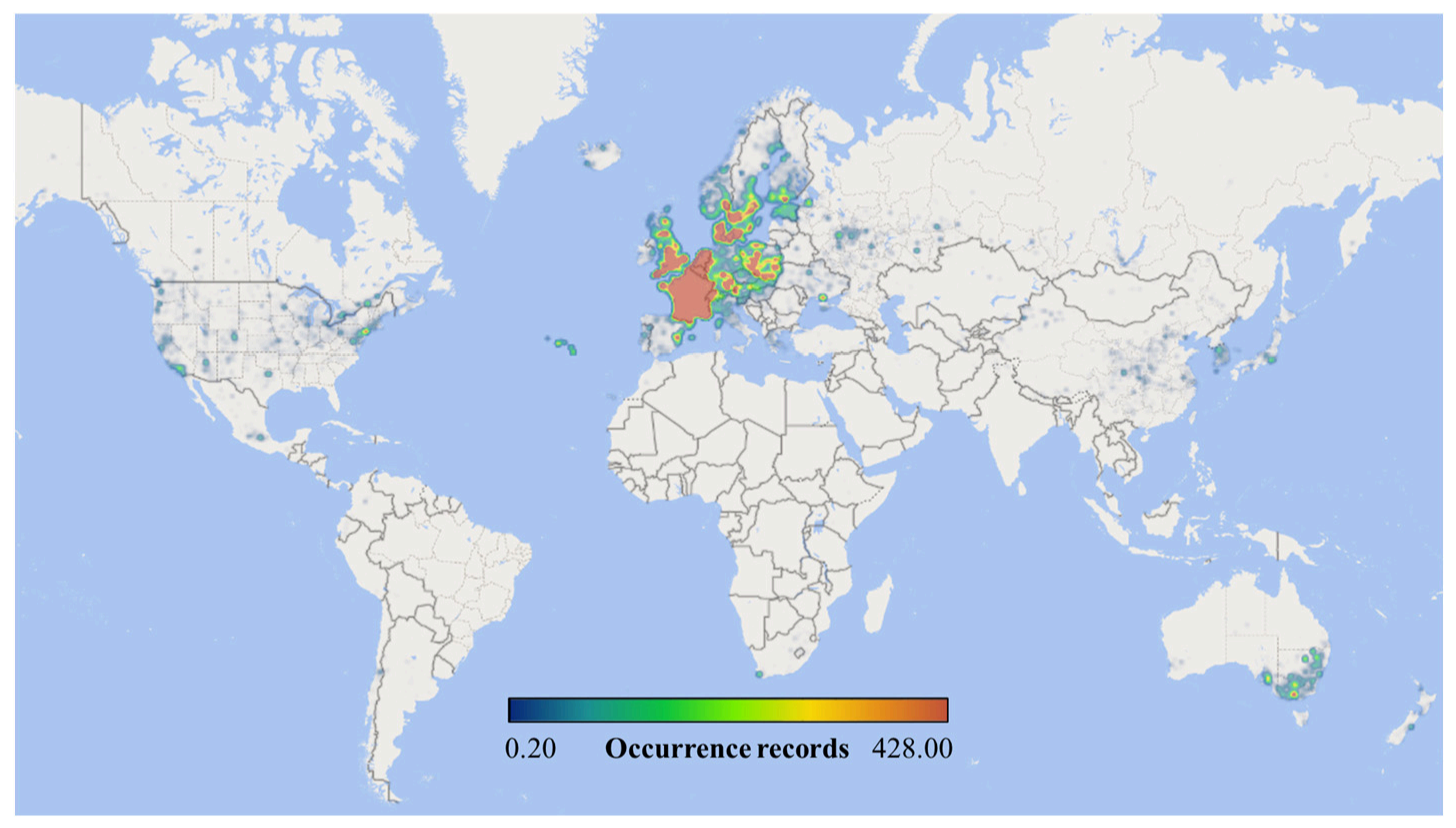
Global distribution map of *Polygonum aviculare*, illustrating its native range in Eurasia and its widespread introduced presence across temperate and subtropical zones worldwide. Occurrence records represent the number of georeferenced specimens and observational data points in the GBIF database (https://www.gbif.org).

*Polygonum aviculare* L. (common knotgrass) has been an integral part of Traditional Chinese Medicine (TCM) for over two millennia, with classical texts such as the *Shennong Bencao Jing* (200 BCE) documenting its use for urinary disorders, including dysuria and urolithiasis. Native to Eurasia, *P. aviculare* is a highly adaptable cosmopolitan species that is now widely distributed across nearly all temperate and subtropical regions globally, as shown in Figure 1 [19]. Its application in treating inflammatory conditions is recorded in the 1596 CE classical text, *Compendium of Materia Medica* (*Ben Cao Gang Mu*), which describes topical preparations for abscesses and skin inflammation. Modern ethnopharmacological studies corroborate these traditional uses globally, documenting hypoglycemic applications in Morocco and the Canary Islands, anti-infective uses for wound treatment in Pakistan, and anti-inflammatory applications for abscesses in Serbia [20]. Its therapeutic applications in folk medicine are attributed to a diverse array of bioactive constituents, including flavonoids, phenolic acids, and tannins [20]. Previous studies have reported antioxidant properties in *P. aviculare* extracts [21], while its anti-inflammatory potential, via the inhibition of pro-inflammatory cytokines, has been preliminarily explored [22]. Additionally, emerging evidence suggests hypoglycemic and antidiabetic effects through α-glucosidase inhibition [23], though systematic evaluations remain limited. Despite its centuries of traditional use, the mechanistic underpinnings of *P. aviculare*’s multifunctional bioactivity remain insufficiently explored, limiting its validation and development as a standardized ingredient for industrial applications. Given its high flavonoid content, which suggests polypharmacological potential against inflammation and metabolic dysregulation, a systematic investigation is crucial to unlock its full potential as a source of high-value, multi-target bioactive compounds for the health product and functional food industries [24].

**Figure 2:**
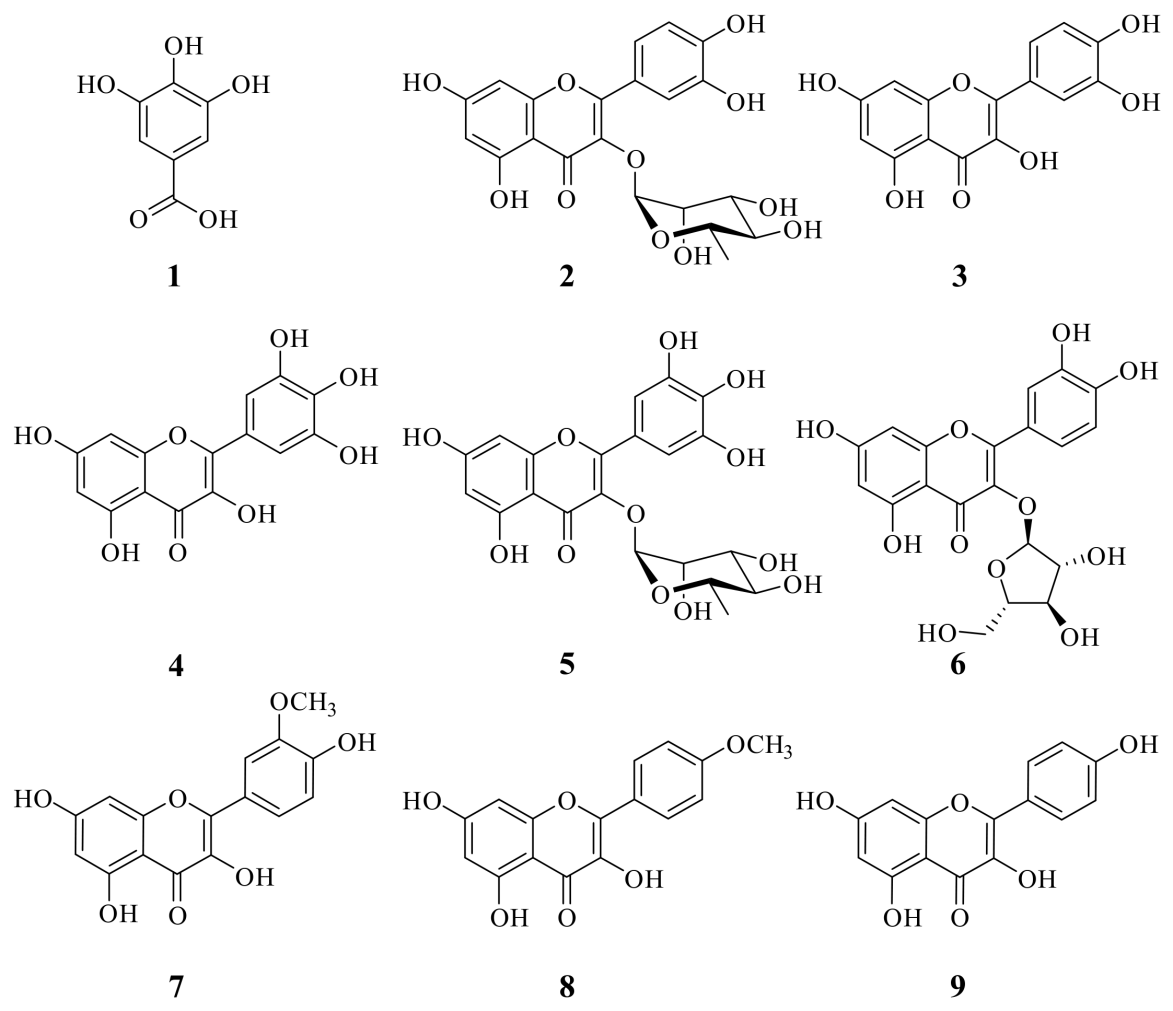
Chemical structures of the nine compounds isolated from the aerial parts of *Polygonum aviculare* L., including gallic acid (**1**), quercitrin (**2**), quercetin (**3**), myricetin (**4**), myricitrin (**5**), avicularin (**6**), isorhamnetin (**7**), kaempferide (**8**), and kaempferol (**9**).

In this study, we isolated nine compounds (Figure 2), including gallic acid (**1**), quercitrin (**2**), quercetin (**3**), myricetin (**4**), myricitrin (**5**), avicularin (**6**), isorhamnetin (**7**), kaempferide (**8**), and kaempferol (**9**) from *P. aviculare* and evaluated their antioxidant, anti-inflammatory, and anti-α-glucosidase activities. Computational docking identified stable interactions between key compounds and therapeutic targets for α-glucosidase and iNOS. Natural products are a rich source of therapeutic agents, with many drugs derived from plant sources [25,26,27]. *P. aviculare* fits this trend, offering a blend of historical use and untapped pharmacological potential. Our work bridges traditional knowledge with modern validation, positioning *P. aviculare* as a sustainable source of multifunctional bioactive compounds for future investigation targeting inflammation, oxidative stress, and metabolic disorders.

## 2. Materials and Methods

### 2.1. Chemical Reagents

All chemical reagents were procured from certified commercial sources, with primary materials obtained from Sigma-Aldrich (St. Louis, MO, USA) (DMSO, ABTS, DPPH, Trolox, gallic acid, Folin–Ciocalteu reagent, BSA, SDS, EDTA, NADPH, FMN, FAD, BH-4, HEPES, DTT, LPS, iNOS, arginine, α-glucosidase), Tokyo Chemical Industry (Tokyo, Japan) (NBT, PMS), SHOWA Chemical (Tokyo, Japan) (potassium peroxodisulfate, Na_2_HPO_4_, NaH_2_PO_4_), Merck (Darmstadt, Germany) (EtOH, TLC plates 60 F-254, silica gel 70–230, 230–400 mesh), Alfa Aesar (Ward Hill, MA, USA) (FeCl_3_, AlCl_3_, *p*-NPG), Acros Organics (Geel, Belgium) (potassium acetate, acarbose, BHT, NADH), MedChemExpress (Monmouth Junction, NJ, USA) (quercetin), J.T. Baker (Phillipsburg, NJ, USA) (glycine), and Bio-Rad (Hercules, CA, USA) (TEMED, ammonium persulfate). ^1^H-NMR spectra (400/600 MHz, Bruker, Billerica, MA, USA) were recorded with *J* in Hz and δ in ppm (abbreviations: br, s, d, t, q, m). UV spectra (Hitachi U-200, Tokyo, Japan) and IR spectra (Shimadzu IR Affinity-1S, Kyoto, Japan) were determined; ESI-MS data were acquired on a Bruker APEX II (Bruker, Billerica, MA, USA). Chromatographic separations employed silica gel column chromatography (CC), thin-layer chromatography (TLC), or preparative thin-layer chromatography (PTLC). Full spectral data are shown in the Supporting Information (Figures S1–S27).

### 2.2. Material of Plant

The aerial parts of *P. aviculare* were acquired from Guangwin Trade Co., Ltd. (Taipei, Taiwan) in September 2024 and identified by Prof. J.-J. Chen through morphological comparison with the voucher specimen HAST:90842 deposited at the Herbarium, Biodiversity Research Museum, Academia Sinica, Taipei, Taiwan.

### 2.3. Plant Material Extraction

The aerial parts of *P. aviculare* were air-dried, pulverized, and extracted with different solvents, including *n*-hexane, ethanol, methanol, deionized water, acetone, ethyl acetate, and dichloromethane. Briefly, 20 g of powdered material was mixed with 120 mL of each solvent, sonicated for 1 h, and subsequently macerated at room temperature for 3 days. The extracts were filtered and concentrated under reduced pressure at 37 °C using a rotary evaporator. For hot-water extraction, 40 g of powdered material was immersed in 800 mL of deionized water for 30 min and then decocted at 100 °C for 30 min until the volume was reduced to approximately 400 mL. The extract was subsequently filtered and concentrated under reduced pressure at 37 °C. All solvent extracts were stored at −20 °C for subsequent analysis.

### 2.4. Extraction and Component Isolation

**Figure 3:**
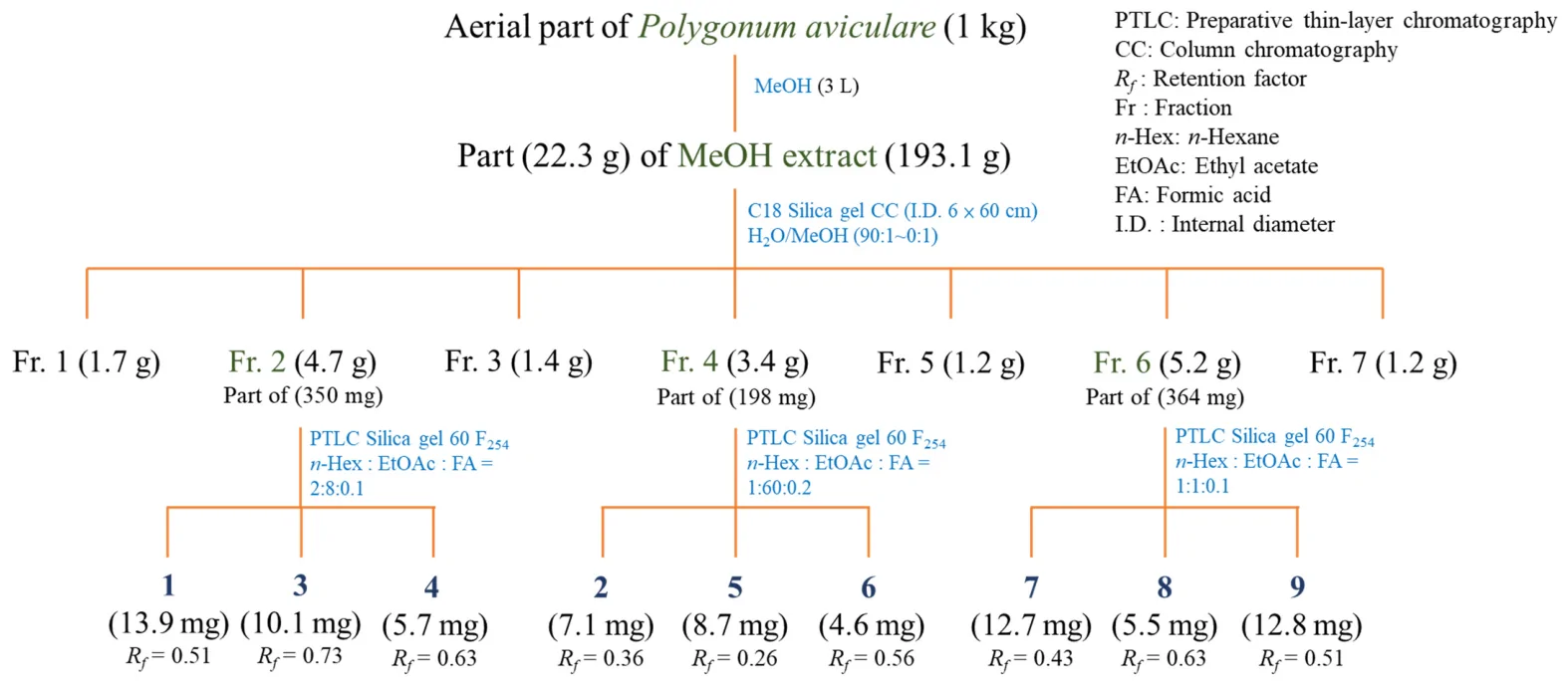
Workflow for the extraction and isolation of *P. aviculare* constituents.

The aerial parts of *P. aviculare* (1 kg) were extracted with 3 L of MeOH, and concentrated under reduced pressure to yield a crude extract (193.1 g). A portion (22.3 g) of the MeOH extract was subjected to C18 silica gel column chromatography (I.D. 6 × 60 cm, H_2_O/MeOH, 90:1 → 0:1) to obtain seven fractions: Fr. 1–Fr. 7. Part (350 mg) of Fr. 2 was separated by PTLC (silica gel 60 F_254_, *n*-hexane/EtOAc/formic acid = 2:8:0.1) to give gallic acid (13.9 mg, *R_f_* = 0.51), quercetin (10.1 mg, *R_f_* = 0.73), and myricetin (5.7 mg, *R_f_* = 0.63). Part (198 mg) of Fr. 4 was separated by PTLC (silica gel 60 F_254_, *n*-hexane/EtOAc/formic acid = 1:60:0.2) to give compounds quercitrin (7.1 mg, *R_f_* = 0.36), myricitrin (8.7 mg, *R_f_* = 0.26), and avicularin (4.6 mg, *R_f_* = 0.56). Part (364 mg) of Fr. 6 was separated by PTLC (silica gel 60 F_254_, *n*-hexane/EtOAc/formic acid = 1:1:0.1) to give isorhamnetin (12.7 mg, *R_f_* = 0.43), kaempferide (5.5 mg, *R_f_* = 0.63), and kaempferol (12.8 mg, *R_f_* = 0.51). The isolated compounds, including gallic acid [28], quercitrin [29], quercetin [30], myricetin [31], myricitrin [32], avicularin [33], isorhamnetin [34], kaempferide [35], and kaempferol [36] were elucidated via NMR, and the structures are depicted in Figure 3. Additionally, the chemical profile of the crude MeOH extract and the peak assignments of the isolated compounds **1**–**9** were verified and confirmed using HPLC analysis (Figure S29).

### 2.5. Total Phenolic Content (TPC) Determination

The TPC was quantified via Folin–Ciocalteu (FC) assay [37]. Samples were prepared at 100 μg/mL in deionized water and reacted with 0.5 N FC reagent (1:1 *v*/*v*), followed by the addition of 20% Na_2_CO_3_ (400 μL) and dark incubation for 40 min at a temperature of 25 °C. Absorbance was measured at 750 nm using a microplate reader, and TPC was calculated from a gallic acid standard curve, expressed as mg gallic acid equivalents per gram (mg GAE/g).

### 2.6. Total Flavonoid Content (TFC) Determination

The TFC was measured using an aluminum chloride (AlCl_3_) colorimetric assay [38]. The tested sample was prepared at 100 μg/mL in MeOH. Then, the tested sample solution (200 μL) was combined with 100 μL of 10% AlCl_3_ solution and 0.1 mM potassium acetate (100 μL), followed by 30 min incubation at 25 °C. Absorbance was measured at 415 nm with microplate reader. The TFC was quantified using the quercetin calibration curve and reported in milligram quercetin equivalents per gram (QE mg/g).

### 2.7. Determination of DPPH Scavenging Capability

DPPH radical scavenging assay was implemented according to the previously described method [39], with butylated hydroxytoluene (BHT) serving as the positive control. An ethanolic DPPH solution (400 μM) was prepared for the experiment. Test sample concentrations (400, 200, 100, 50, and 25 µg/mL) were combined with the DPPH solution in equal volumes (100 μL each) and reacted for 30 min under dark conditions. Absorbance measurements were taken at 520 nm with a microplate reader. The radical scavenging capacity was calculated as a percentage inhibition relative to the control.

### 2.8. Determination of ABTS Scavenging Capability

The ABTS assay was assessed using the reported method [39]. The stock solution was prepared by reacting 28 mM ABTS solution with 9.6 mM potassium persulfate (1:1 *v*/*v* in distilled water), followed by 16 h of dark incubation at 4 °C. The solution was adjusted with ethanol to an absorbance of 0.70 ± 0.02 at 740 nm. Test samples (10 μL) at concentrations ranging between 400, 200, 100, 50, and 25 µg/mL were mixed with 190 μL of the working solution, followed by 6 min incubation at room temperature. The antioxidant activity was quantified spectrophotometrically by monitoring absorbance reduction at 740 nm. Results were expressed as percentage inhibition relative to the blank control.

### 2.9. Superoxide Radical Scavenging Activity

The superoxide radical scavenging capacity was evaluated according to established methodology [9] with cynaroside as a positive control. Cynaroside (luteolin-7-*O*-glucoside) was selected as the positive control for this assay because it is a structurally related flavonoid with well-documented and highly potent superoxide-scavenging properties, serving as a reliable benchmark for evaluating the radical-scavenging capacity of the isolated constituents. The reaction system contained 16 mM Tris-HCl buffer (pH 8.0) supplemented with 50 μL each of NBT (300 μM), PMS (120 μM), and different concentrations of the test sample. The superoxide production was activated by adding 50 μL of NADH (468 μM), followed by 5 min incubation at 25 °C. Antioxidant activity was calculated by measuring the reduction in absorbance at 560 nm with microplate reader. Scavenging capacity was calculated relative to sample concentration and expressed as percentage inhibition.

### 2.10. Ferric Reducing Antioxidant Power (FRAP)

The ferric reducing capacity was determined following the FRAP assay protocol [40]. The working reagent, freshly prepared by mixing acetate buffer (pH 3.6), 20 mM FeCl_3_, and 10 mM TPTZ (in 40 mM HCl) at 10:1:1 (*v*/*v*/*v*), was pre-warmed to 37 °C. Samples or standards (100 μL) were reacted with 900 μL reagent in microcentrifuge tubes, vortexed, and incubated (37 °C, 40 min) before measuring absorbance at 593 nm. Results were quantified against a Trolox standard curve (0–100 mM) and expressed as mM TE/g dry weight, with samples beyond the linear range diluted and reanalyzed.

### 2.11. α-Glucosidase Inhibitory Activity Assay

α-Glucosidase (from *Saccharomyces cerevisiae*; Type I, lyophilized powder, ≥10 units/mg protein; Catalog No. G5003, Lot No. 0000338460) was purchased from Sigma-Aldrich (St. Louis, MO, USA). The α-glucosidase inhibitory activity was assessed by incubating test samples (100 μL) with enzyme solution (1 U/mL, 20 μL) in phosphate buffer (pH 6.8) for 40 min at 37 °C, initiating the reaction with *p*-NPG substrate (0.53 mM, 380 μL), terminating with Na_2_CO_3_ (0.1 M, 500 μL), and measuring absorbance at 405 nm, using acarbose as a positive control. The calculation of α-glucosidase inhibition (%) is shown below. $A_{\mathrm{sample}}\;$ and $A_{\mathrm{control}}$ denote the absorbance values of the tested sample and blank control, respectively.

$$\alpha \text{-}\mathrm{Glucosidase}\;\mathrm{inhibition}\;(\%)=\frac{A_{\mathrm{control}}-A_{\mathrm{sample}}}{A_{\mathrm{control}}}\times 100\%$$

### 2.12. Cell Culture

The RAW 264.7 murine macrophage cell line utilized in these in vitro assays was originally obtained from Bioresource Collection and Research Center (BCRC, Hsinchu, Taiwan; No. 60001). Cells were maintained in Dulbecco’s modified Eagle’s medium (DMEM; HiMedia, Mumbai, Maharashtra, India) containing 10% fetal bovine serum (FBS; Product No. 89510-886, Avantor, Radnor, PA, USA) and 1% penicillin–streptomycin (Gibco, Thermo Fisher Scientific, Waltham, MA, USA). Cell cultures were maintained at 37 °C in a humidified incubator with 5% CO_2_. Dulbecco’s phosphate-buffered saline (DPBS, Elabscience Biotechnology Inc., Wuhan, China) was purchased from Biological Industries (Foreston, MN, USA), and Cell banker 1 used for cell storage was obtained from ZENOAQ (Fukushima, Japan) [11].

### 2.13. Nitric Oxide (NO) Inhibition Assay

NO production was assessed in LPS-stimulated RAW 264.7 macrophages using a modified Griess assay [11]. Cells were seeded in 96-well plates (2 × 10^4^ cells/well) 24 h before experimentation. Following 1 h pretreatment with test compounds (37 °C, 5% CO_2_), inflammation was induced with LPS (100 ng/mL). After 20 h incubation, culture supernatants (50 μL) were mixed with Griess reagent (50 μL) for 20 min, and nitrite production was quantified by measuring absorbance at 550 nm.

### 2.14. Cell Viability Assay

Cell viability was determined using the MTT assay. This method is an improvement upon Mosmann’s method [41].

### 2.15. Western Blot Analysis

Protein expression was assessed by Western blotting [11]. Cells (1 × 10^6^) were seeded in 6 cm culture dishes and cultured for 24 h. The cells were treated with the tested sample in the presence of LPS (100 ng/mL) and incubated for 24 h. The medium was then removed, and cells were washed twice with 1 mL of DPBS. Cells were harvested in RIPA buffer by scraping, collected, and then stored at −80 °C. The following day, samples were centrifuged at 15,000 rpm for 30 min at 4 °C, and the supernatant was collected and stored at −80 °C as protein samples. For sodium dodecyl sulfate–polyacrylamide gel electrophoresis (SDS-PAGE), 8% polyacrylamide separating gel and 5% polyacrylamide stacking gel were prepared. Protein samples were mixed with 4× sample dye (300 mM Tris-Cl, 12% SDS, 0.6% bromophenol blue, 40% glycerol, and 200 mM β-mercaptoethanol) to a fixed volume, heated at 100–110 °C for 10 min, and then cooled on ice. After centrifugation, the samples were loaded into wells of the gel and subjected to SDS-PAGE for electrophoresis. Then, proteins were transferred to a polyvinylidene difluoride (PVDF) membrane at 100 V for 120 min. Following protein transfer, the PVDF membranes were horizontally cut into distinct strips based on pre-stained molecular weight markers. This membrane cutting procedure allowed for the simultaneous incubation and detection of target proteins with significantly different molecular weights, such as iNOS (130 kDa), COX-2 (74 kDa), KLF4 (60 kDa), and the internal control β-actin (43 kDa), on separate membrane sections, thereby avoiding the need for stripping and reprobing for these specific targets. The cut PVDF membrane strips were blocked with blocking buffer (2% BSA in TBST or 5% nonfat milk in TBST) at room temperature for 1 h and then incubated with their respective primary antibodies (1:1000–1:500) overnight at 4 °C. The membranes were then washed three times with TBST for 5 min each, followed by incubation with the secondary antibody (1:3000) at room temperature for 1 h. After three additional washes with TBST, enhanced chemiluminescence (ECL) was used to visualize protein bands, and luminescence was captured using a fluorescence imaging system. Conversely, for target proteins with identical or highly similar molecular weights evaluated from the same gel, such as phosphorylated form versus their total protein expression or closely migrating targets, stripping and reprobing procedures were performed. To analyze these targets, the PVDF membrane was treated with stripping buffer at room temperature for 10 min, washed twice with TBST for 3 min each, reblocked, and subsequently reprobed with primary and secondary antibodies as previously described. Chemiluminescent signals were captured using ImageQuant LAS 4000, with exposure times adjusted individually for each target protein to achieve optimal signal intensity without saturation. Protein bands were detected by enhanced chemiluminescence. For densitometric quantification, protein band intensities were analyzed using ImageJ software (version 1.53, National Institutes of Health, Bethesda, MD, USA). The expression levels of iNOS, COX-2, KLF4, Arg-1, p38, phospho-p38 (p-p38), IκBα, and phospho-IκBα (p-IκBα) were normalized to β-actin as the internal loading control.

### 2.16. Molecular Modeling Docking Study

Molecular docking simulations were conducted with Discovery Studio 2019 (Dassault Systèmes BIOVIA, San Diego, CA, USA) [9]. Ligand structures were generated in ChemDraw Ultra 12.0 and energy-minimized using the MMFF94 force field. The crystal structures of *Saccharomyces cerevisiae* isomaltase (PDB ID: 3A4A) and *Mus musculus* iNOS (PDB ID: 1M9T) can be found in the Protein Data Bank (https://www.rcsb.org/structure/3A4A and https://www.rcsb.org/structure/1M9T, respectively; accessed on 25 February 2026). Receptor preparation involved addition of hydrogen atoms and energy minimization to the protein structures prior to docking. Gasteiger–Marsili partial charges were assigned to ligands; non-polar hydrogens were merged; and files were saved in PDBQT format. For iNOS (PDB: 1M9T), the docking grid box was centered on the active site at x = 124.8, y = 115.4, z = 30.9 with dimensions of 25 × 25 × 25 Å. For α-glucosidase (PDB: 3A4A), the grid box was centered at x = 21.2, y = −0.8, z = 18.6 with dimensions of 18 × 18 × 18 Å. Molecular docking was performed with the exhaustiveness parameter set to 20, and the top 10 binding poses ranked by calculated free energy of binding (ΔG, kcal/mol). Docking reliability was validated by re-docking the co-crystallized ligand into its respective binding site; the resulting RMSD of 0.3947 Å (<2.0 Å) confirmed the suitability of the docking protocol (Figure S30). Binding site residues and ligand–protein interactions were identified and visualized using Discovery Studio Visualizer 2021 through three-dimensional analysis of optimal docking conformations.

### 2.17. Drug-Likeness Score

Drug-likeness parameters (Lipinski’s Rule, TPSA, BBB score, solubility) were calculated using MolSoft’s cheminformatics toolkit, with compounds meeting all criteria for oral bioavailability (https://molsoft.com/mprop/), accessed on 25 February 2026 [42].

### 2.18. Statistical Analysis

Triplicate experiments (*n* = 3) were expressed as the mean ± standard deviation (SD). Statistical analysis was determined by Student’s *t*-test, with significance defined as * *p* < 0.05, ** *p* < 0.01, and *** *p* < 0.001, respectively.

## 3. Results and Discussion

### 3.1. TPC and TFC of Different Solvent Extracts

The TPC and TFC of various solvent extracts of the aerial part of *P. aviculare* were determined, and the results are indicated in Table 1. The 100 °C water extract (102.91 ± 12.79 GAE mg/g) displayed the highest TPC, followed by the methanol extract (80.27 ± 4.66 GAE mg/g) and the ethanol extract (74.49 ± 9.06 GAE mg/g). In contrast, *n*-hexane extract showed the lowest TPC. For TFC, methanol extract (72.98 ± 7.42 QE mg/g) and acetone extract (71.08 ± 6.64 QE mg/g) demonstrated the highest values, while again *n*-hexane extract (7.52 ± 0.86 QE mg/g) had the lowest TFC. This suggests that most phenols and flavonoids are concentrated in the polar layers, particularly when extracted with boiling water. In contrast, the non-polar layer exhibits limited phenol and flavonoid content, indicating that these compounds are primarily associated with hydroxyl moieties.

### 3.2. DPPH Radical Scavenging Effect

The antioxidant capacity of solvent extracts and isolated compounds was quantitatively evaluated using DPPH radical scavenging assays, with comparative results shown in Table 2 and Table 3. BHT was used as the positive control. The methanol extract (SC_50_ = 77.04 ± 5.99 μg/mL) demonstrated the strongest DPPH radical scavenging activity among the solvent extracts, followed by the 100 °C water extract (SC_50_ = 100.76 ± 7.08 μg/mL) and ethanol extract (SC_50_ = 134.43 ± 2.06 μg/mL). The ethyl acetate extract (SC_50_ = 248.78 ± 6.53 μg/mL) also showed significant activity. Among the pure compounds, gallic acid (SC_50_ = 3.37 ± 0.82 μM) possessed the strongest DPPH radical scavenging effect, followed by myricetin (SC_50_ = 3.55 ± 0.48 μM), quercetin (SC_50_ = 4.31 ± 0.51 μM), isorhamnetin (SC_50_ = 4.60 ± 0.32 μM), and avicularin (SC_50_ = 6.50 ± 0.91 μM). These findings indicate that the number of hydroxyl groups is directly related to the activity, as quercetin and myricetin, with the most OH groups, showed the highest antioxidant activity, while kaempferide exhibited the least efficiency. The DPPH radical scavenging activities of gallic acid, myricetin, quercetin, avicularin, and isorhamnetin were all more potent than the positive control BHT (SC_50_ = 36.99 ± 4.54 μM).

### 3.3. ABTS Cation Radical Scavenging Effect

The antioxidant capacity of solvent extracts and isolated compounds was evaluated through ABTS radical scavenging assays, with BHT serving as the positive control. The results were presented in Table 2 and Table 3. The 100 °C water extract showed the strongest free radical scavenging activity against ABTS, with SC_50_ values of 36.04 ± 0.46 μg/mL, followed by the ethanol extract, with SC_50_ values of 47.50 ± 0.92 μg/mL. Most of the isolated components possessed greater ABTS radical scavenging effects than BHT (SC_50_ = 17.36 ± 3.14 μM). Among the pure compounds, gallic acid displayed the strongest activity (SC_50_ = 6.46 ± 0.60 μM), followed by quercetin (SC_50_ = 7.89 ± 0.83 μM), myricetin (SC_50_ = 9.62 ± 1.77 μM), and avicularin (SC_50_ = 12.44 ± 2.53 μM). The ABTS assay demonstrated better detection of antioxidant activity for aglycone flavonoids (compounds **2**, **5**, and **6**) compared to the DPPH assay, attributable to the enhanced aqueous compatibility of ABTS chemistry that facilitates optimal interaction with polar antioxidants. Conversely, methoxylated flavonoids like isorhamnetin and kaempferide exhibited reduced efficacy in ABTS.

### 3.4. Superoxide Radical Scavenging Effect

The 100 °C water extract (SC_50_ = 56.34 ± 3.47 μg/mL) exhibited measurable superoxide scavenging effects. Among the pure compounds, quercetin (SC_50_ = 30.67 ± 3.30 μM) and myricetin (SC_50_ = 35.54 ± 5.01 μM) showed the strongest activity. Based on the structural principles, isorhamnetin, kaempferide, and kaempferol were less effective compared with quercitrin, quercetin, myricetin, myricitrin, and avicularin in superoxide, suggesting that the dihydroxy moiety on the B ring is critical for optimal radical stabilization.

### 3.5. Ferric Reducing Antioxidant Power

The antioxidant capacity of extracts and compounds was measured as Trolox equivalents (TE)/g via ferric reduction. Among the solvent extracts, the 100 °C water extract demonstrated the strongest reducing power (TE = 651.23 ± 6.53 mM/g), followed by the ethanol extract (TE = 604.22 ± 6.50 mM/g) and ethyl acetate extract (TE = 449.96 ± 5.82 mM/g). The *n*-hexane extract displayed minimal activity (TE = 82.35 ± 2.34 mM/g).

For pure compounds, quercetin (TE = 25,511.50 ± 88.61 mM/g) showed the highest reducing activity, outperforming myricetin (TE = 15,243.65 ± 39.74 mM/g), isorhamnetin (TE = 15,026.44 ± 63.13 mM/g), and gallic acid (TE = 9871.37 ± 42.34 mM/g). All four compounds significantly surpassed the positive control BHT (TE = 4561.78 ± 12.93 mM/g).

Among flavonoid derivatives, the structural features of quercetin and myricetin, particularly their hydroxyl group substitution patterns, correlate with their superior antioxidant activity. Specifically, a higher number of hydroxyl groups enhances antioxidant potency; for example, quercetin and myricetin possess the greatest number of hydroxyl groups, and the antioxidant activities are the greatest compared with kaempferide. In contrast, glycosylation consistently reduces antioxidant potential, as seen in the diminished performance of glycosylated derivatives relative to their aglycone forms. Quercitrin is the rhamnoside of quercetin, whereas avicularin is its arabinoside. Notably, both glycosides exhibit varying levels of effectiveness, with avicularin exhibiting greater antioxidant activity. A similar pattern occurs with myricetin and myricitrin. The FRAP assay reveals a more obvious trend for glycosides quercitrin, myricitrin, and avicularin (TE < 4000 mM/g). The effect of methoxy depends on its position on the flavonoid. Adding a methoxy group at the C-3′ position, such as isorhamnetin, retains some antioxidant activity but impairs superoxide scavenging ability. Adding a methoxy group at the C-4′ position, such as kaempferide, impairs all antioxidant activity. These results indicate that keeping the C-4′ hydroxyl group free from methoxy is critical for maintaining strong antioxidant activity. Isorhamnetin, kaempferide, and kaempferol showed reduced superoxide scavenging, suggesting that the position of the OH group is more favorable when it is *ortho*-adjacent.

### 3.6. α-Glucosidase Inhibitory Effect

As shown in the tables (Table 4), all solvent extracts and isolated components from *P. aviculare* were evaluated for anti-α-glucosidase inhibitory activity. Acarbose was employed as the positive control. Among all solvent extracts, the ethanol extract showed the best inhibitory activity against α-glucosidase (IC_50_ = 138.62 ± 16.93 μg/mL), followed by the acetone extract (IC_50_ = 162.99 ± 14.91 μg/mL), water 100 °C extract (IC_50_ = 187.67 ± 12.98 μg/mL), and ethyl acetate extract (IC_50_ = 195.01 ± 4.86 μg/mL). The ethanol, acetone, water at 100 °C, and ethyl acetate displayed more effective inhibition against α-glucosidase than acarbose (IC_50_ = 368.67 ± 21.40 μg/mL). Among the isolated compounds (Table 5), kaempferol displayed the most potent anti-α-glucosidase activity (IC_50_ = 4.5 ± 0.90 μM), followed by myricetin (IC_50_ = 9.25 ± 0.17 μM), quercetin (IC_50_ = 13.4 ± 2.88 μM), kaempferide (IC_50_ = 31.3 ± 3.90 μM), and isorhamnetin (IC_50_ = 33.9 ± 2.17 μM). Gallic acid, quercetin, myricetin, avicularin, kaempferol, kaempferide, and isorhamnetin possessed more powerful α-glucosidase inhibitory effects than acarbose (IC_50_ = 519.7 ± 18.67 μM). The structure–activity relationship (SAR) revealed that glycosylation markedly reduced inhibitory potency, with suppression severity dependent on sugar moiety. Rhamnose conjugates (quercitrin and myricitrin) exhibited the greatest activity loss, followed by arabinose derivatives (avicularin), showing the same pattern across antioxidant activity assays.

### 3.7. Anti-Inflammation Assay

The anti-inflammatory potential was evaluated through the inhibition of nitric oxide (NO) production in LPS-stimulated RAW 264.7 macrophages. As shown in Table 6, the acetone extract showed the best inhibitory activity against NO production (IC_50_ = 28.57 ± 3.18 μg/mL), followed by the ethanol extract (IC_50_ = 30.69 ± 3.84 μg/mL) and the methanol extract (IC_50_ = 31.04 ± 1.89 μg/mL). Among the isolated compounds shown in Table 7, quercetin exhibited the strongest NO inhibition (IC_50_ = 5.04 ± 1.25 μM), followed by gallic acid (IC_50_ = 12.60 ± 2.03 μM), kaempferol (IC_50_ = 15.06 ± 1.50 μM), isorhamnetin (IC_50_ = 15.50 ± 2.22 μM), and avicularin (IC_50_ = 23.47 ± 3.68 μM). Myricetin and kaempferide were excluded from this efficacy ranking due to observed cytotoxicity at higher concentrations. Notably, quercetin, gallic acid, kaempferol, and isorhamnetin exhibited stronger NO inhibitory activity than apigenin (IC_50_ = 16.08 ± 1.86 μM). Apigenin was strategically selected as the positive control because its fundamental flavone skeleton provides an optimal structural baseline for our comparative structure–activity relationship (SAR) analysis. The use of this structurally related natural inhibitor [43] allowed us to evaluate how specific structural variations, such as B-ring hydroxylation in the isolated *P. aviculare* flavonoids, influence and enhance anti-inflammatory efficacy. This selection is further justified by its robust and reproducible suppression of NO production within our specific RAW 264.7 assay system.

SAR analysis indicated that kaempferide with a methoxy group at C-4′ and displayed reduced biological activity, following the same trend as the antioxidant assays. Notably, a comparison among quercetin (IC_50_ = 5.04 ± 1.25 μM), isorhamnetin (IC_50_ = 15.50 ± 2.22 μM), and kaempferol (IC_50_ = 15.06 ± 1.50 μM) reveals that an *ortho*-dihydroxy configuration (3′,4′-diOH) on the B-ring, as present in quercetin, appears to play a critical role in enhancing NO inhibitory efficacy. Replacing the C-3′ hydroxyl group with a methoxy group (isorhamnetin) or lacking it entirely (kaempferol) results in an approximately three-fold reduction in potency. Furthermore, the comparable activities of kaempferol and the positive control apigenin indicate that the presence of a C-3 hydroxyl group on the C-ring does not markedly influence this specific anti-inflammatory effect.

### 3.8. Cell Viability Assessment

Prior to the NO inhibition assay, the cytotoxic potential of all nine isolated compounds and positive control apigenin was assessed in RAW 264.7 macrophages using the MTT assay across a concentration range of 2.5–100 μM (Figure S28). Except for myricetin and kaempferide, which caused cell death at higher concentrations and were consequently marked as not applicable (N/A) in Table 7, all active compounds maintained cell viability above 80% at their effective NO-inhibitory concentrations. This confirms that the suppression of NO production reflects genuine pharmacological activity rather than a secondary consequence of compromised cellular metabolic capacity. Notably, kaempferol did not exhibit significant cytotoxicity across the entire concentration range.

### 3.9. Western Blot Analysis of Anti-Inflammatory Mechanisms

**Figure 4:**
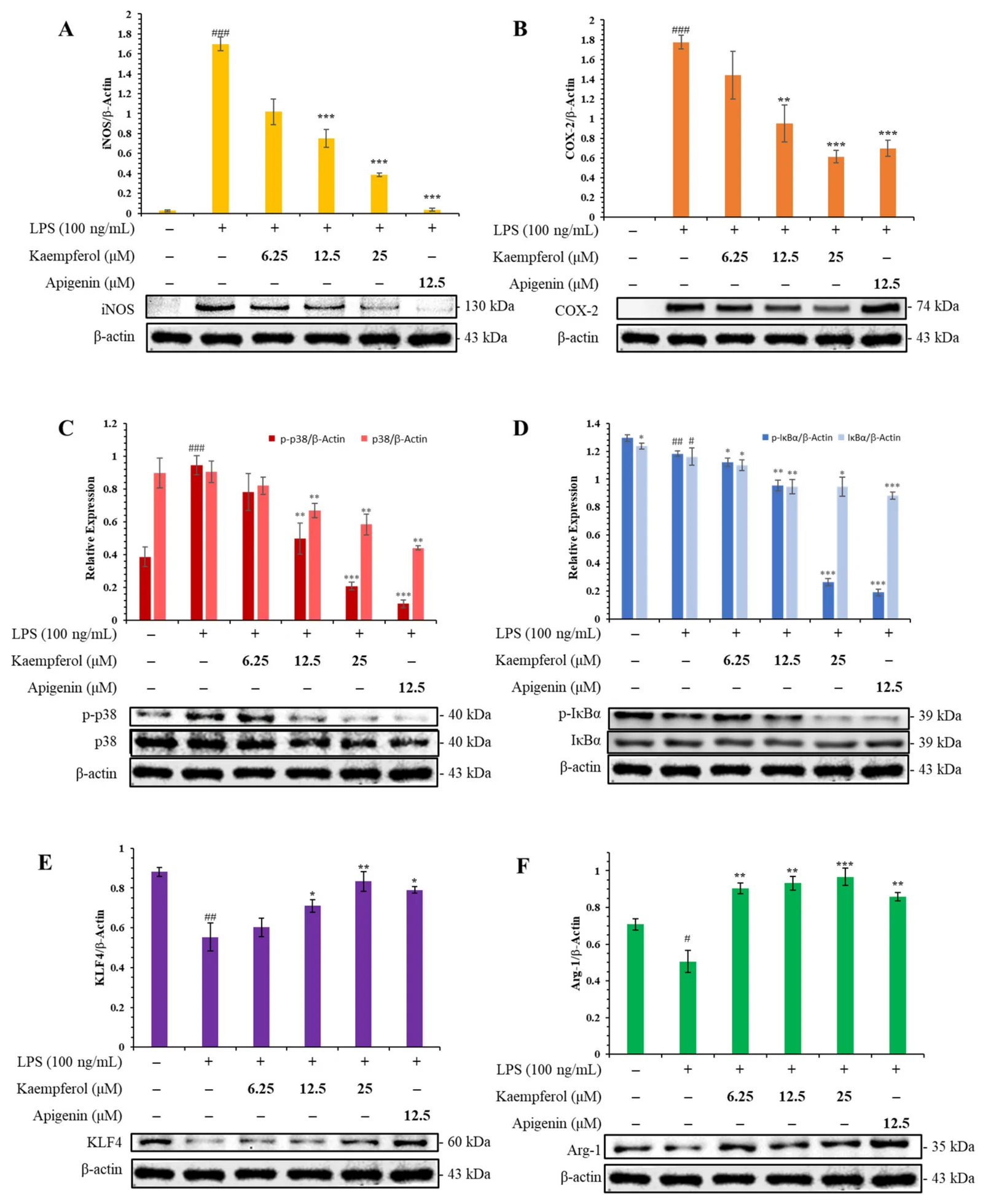
Effects of kaempferol on (**A**) iNOS, (**B**) COX-2, (**C**) p-p38 and p38, (**D**) p-IκBα and IκBα, (**E**) KLF4, and (**F**) Arg-1 in RAW 264.7 cells. Protein expression was analyzed by Western blotting. All target proteins, including phosphorylated forms, were normalized to β-actin as the internal control. Data are expressed as mean ± SD from three independent biological replicates (*n* = 3). # *p* < 0.05, ## *p* < 0.01, and ### *p* < 0.001 compared with the untreated control group; * *p* < 0.05, ** *p* < 0.01, and *** *p* < 0.001 compared with the LPS-stimulated group.

Given its superior cell viability across all tested concentrations, kaempferol was selected to investigate its modulatory effects on key inflammatory signaling pathways. The expression levels of specific proteins were analyzed to elucidate the underlying mechanisms (Figure 4): inducible nitric oxide synthase (iNOS) is a critical enzyme generating excessive nitric oxide (NO) during inflammation [44], and cyclooxygenase-2 (COX-2), which mediates prostaglandin production associated with pain and vasodilation [45]. The MAPK signaling pathway, which includes phosphorylated forms of p38 (p-p38), Erk (p-Erk), JNK (p-JNK), serves as a critical regulator in the production of pro-inflammatory cytokines and mediators [46]. Activation of these kinases can amplify inflammatory responses, making them important targets for anti-inflammatory interventions. On the other hand, IκBα acts as a natural inhibitor of nuclear factor-kappa B (NF-κB) activation by sequestering NF-κB in the cytoplasm, thereby preventing its nuclear translocation and subsequent induction of pro-inflammatory gene expression [47]. Phosphorylation of IκBα (p-IκBα) leads to its degradation, releasing NF-κB and enabling its transcriptional activity [48,49], and Krüppel-like factor 4 (KLF4) and Arginase-1 (Arg-1) are markers associated with the anti-inflammatory M2 macrophage phenotype, which are associated with anti-inflammatory and tissue-repair mechanisms [50,51].

According to the results, LPS stimulation (100 ng/mL) markedly upregulated pro-inflammatory mediators iNOS and COX-2, and concurrently enhanced the phosphorylation of p38 MAPK (p-p38) and IκBα (p-IκBα). Additionally, LPS reduced expression of immunomodulatory factors KLF4 and Arg-1 relative to untreated controls. Kaempferol (**9**) treatment attenuated these LPS-induced alterations in iNOS and COX-2 expression, exhibiting progressive suppression, particularly evident at 25 μM; p-p38 phosphorylation was substantially reduced at higher concentrations, while p-IκBα phosphorylation showed significant inhibition at 25 μM. Conversely, kaempferol treatment markedly upregulated the expression of the M2 polarization markers KLF4 and Arg-1 compared to the LPS-stimulated group. Apigenin (12.5 μM) demonstrated comparable efficacy in suppressing iNOS, COX-2, p-p38, and p-IκBα while restoring KLF4 and Arg-1 expression, consistent with its established anti-inflammatory activity in macrophage models [43]. Collectively, kaempferol was associated with altered expression of multiple inflammation-related mediators, suggesting that its anti-inflammatory effects may involve several signaling pathways; however, further mechanistic studies are required to clarify the underlying molecular basis of this activity.

The present study demonstrates the regulating effects of kaempferol on upregulating KLF4 and Arg-1 expression in LPS-stimulated RAW 264.7 macrophages, suggesting a potential association with M2-like macrophage polarization. Furthermore, whether kaempferol directly drives this polarization and the specific molecular mechanisms involved warrant further investigation.

### 3.10. Molecular Docking Findings

**Figure 5:**
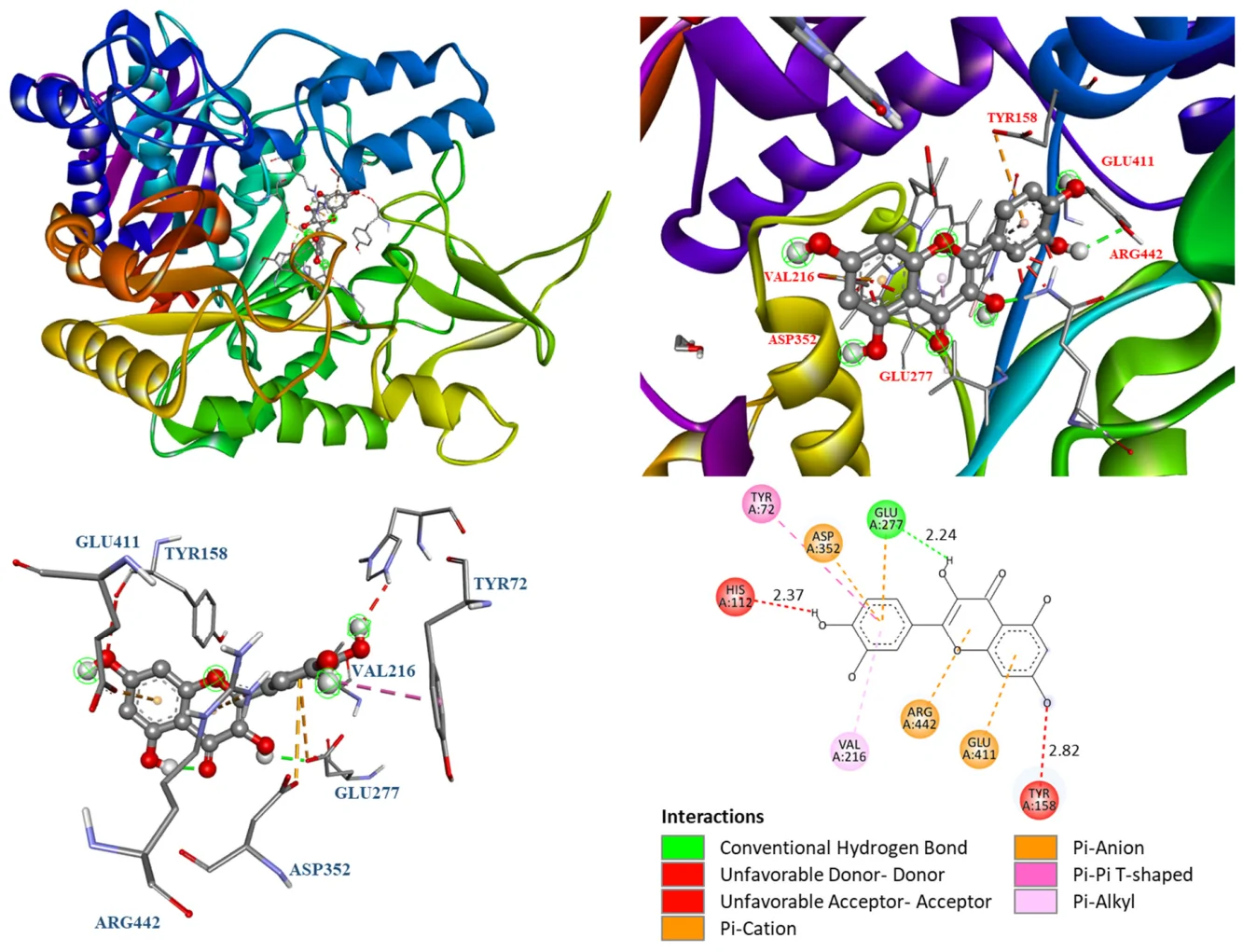
Molecular interactions of quercetin within the active site of *Saccharomyces cerevisiae* isomaltase (PDB ID: 3A4A), utilized as a structural surrogate for α-glucosidase.

**Figure 6:**
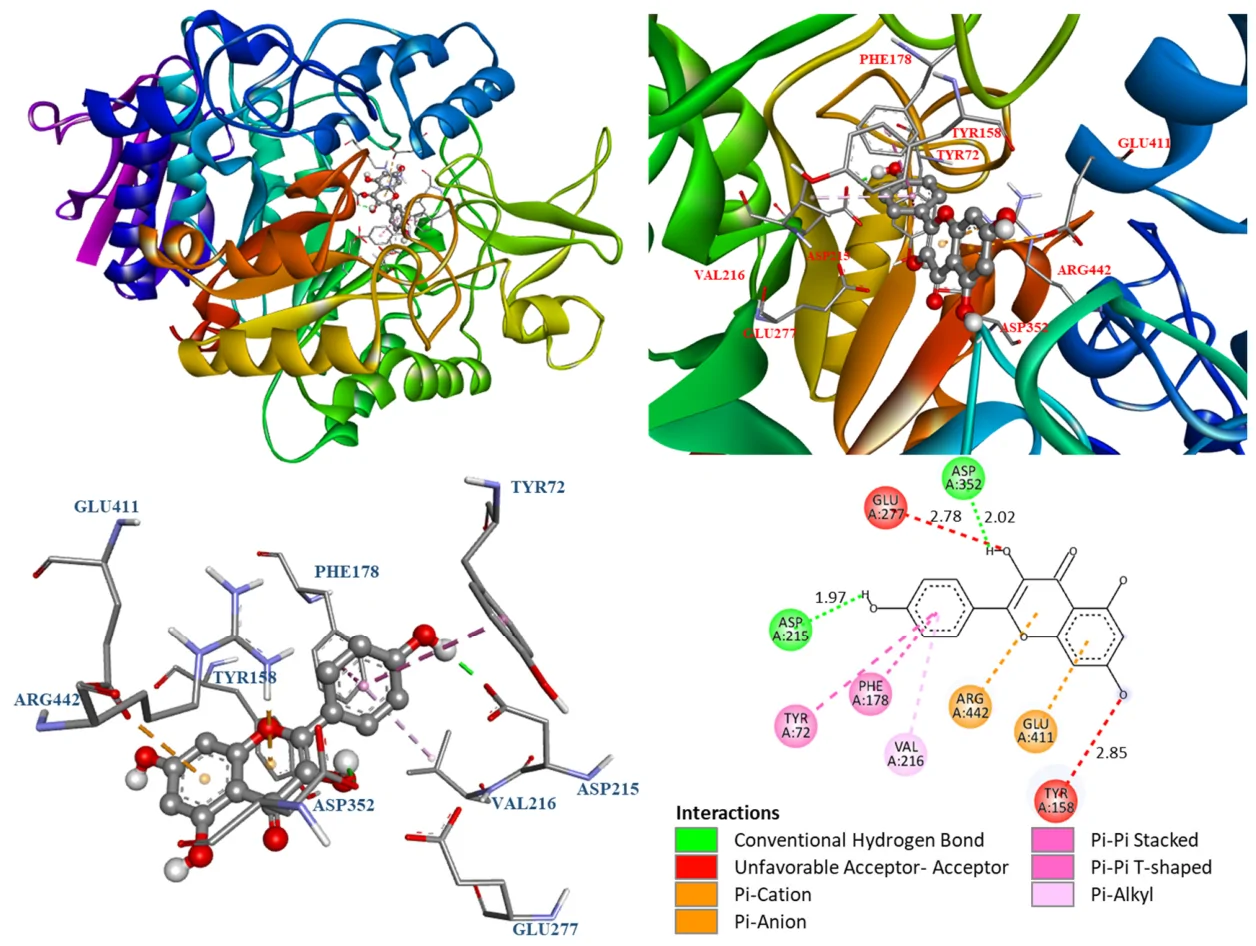
Molecular interactions of kaempferol within the active site of *Saccharomyces cerevisiae* isomaltase (PDB ID: 3A4A), utilized as a structural surrogate for α-glucosidase.

**Figure 7:**
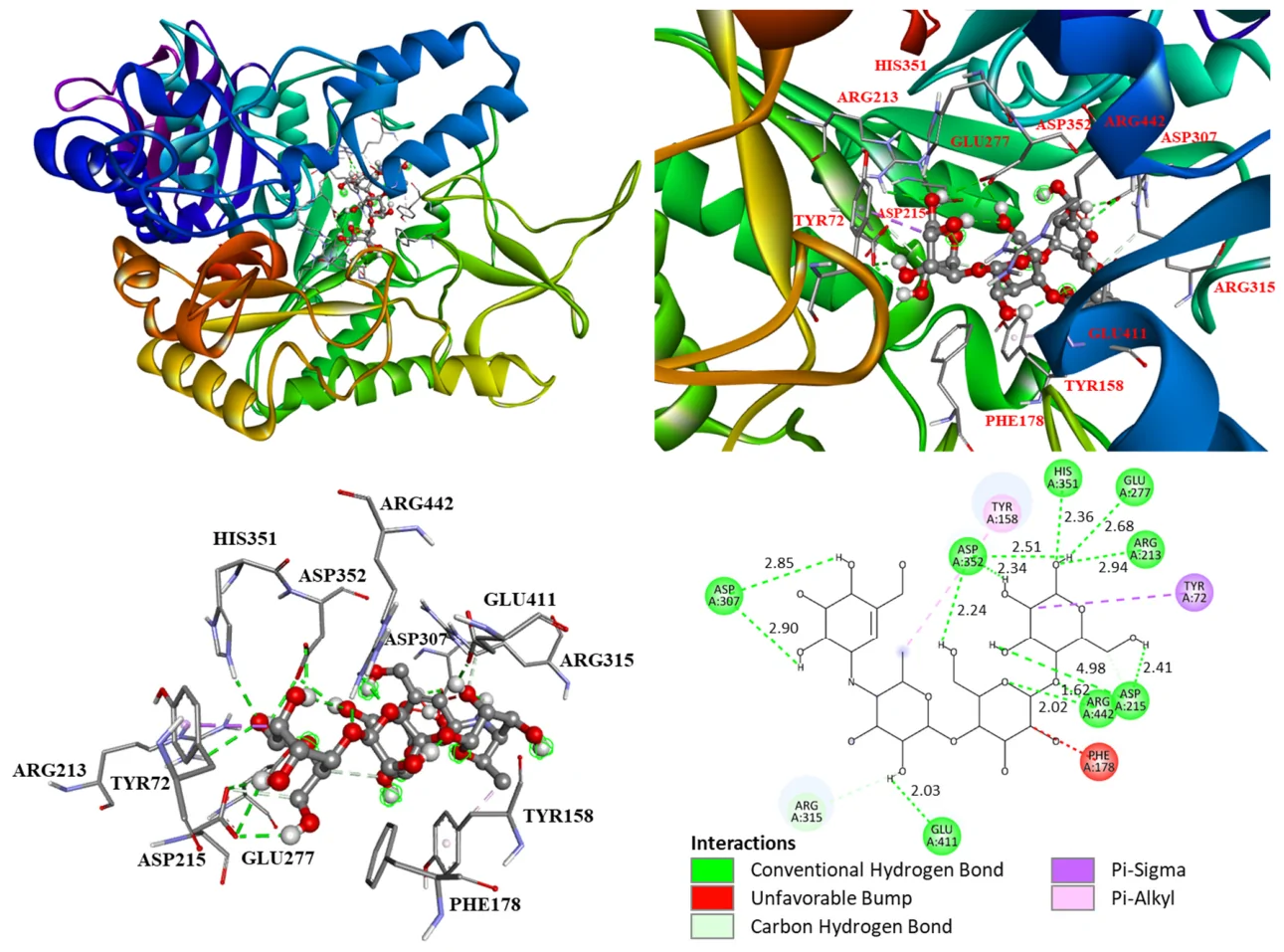
Molecular interactions of acarbose within the active site of *Saccharomyces cerevisiae* isomaltase (PDB ID: 3A4A), utilized as a structural surrogate for α-glucosidase.

**Figure 8:**
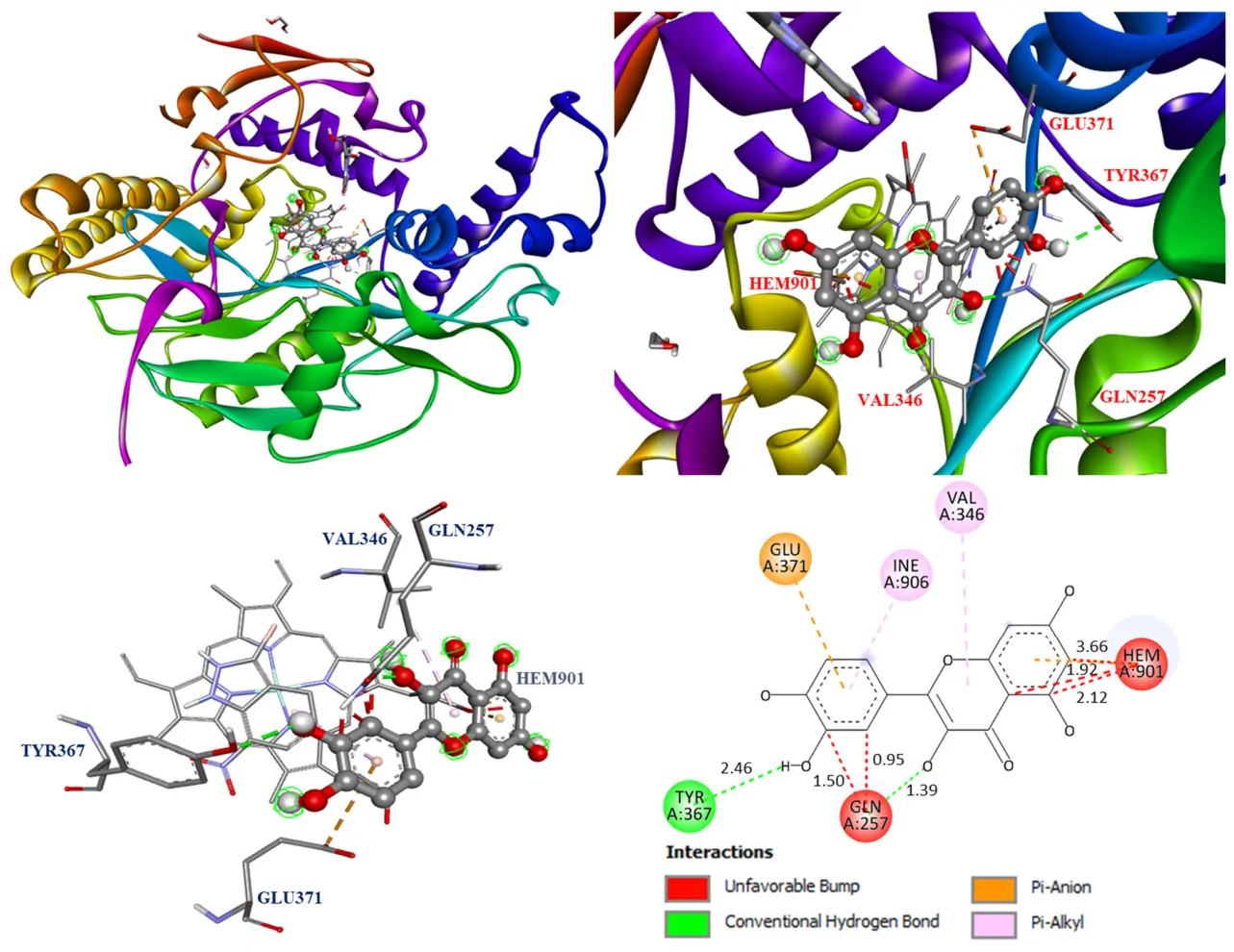
Molecular interactions of quercetin within the active site of *Mus musculus* inducible nitric oxide synthase (iNOS) (PDB ID: 1M9T).

**Figure 9:**
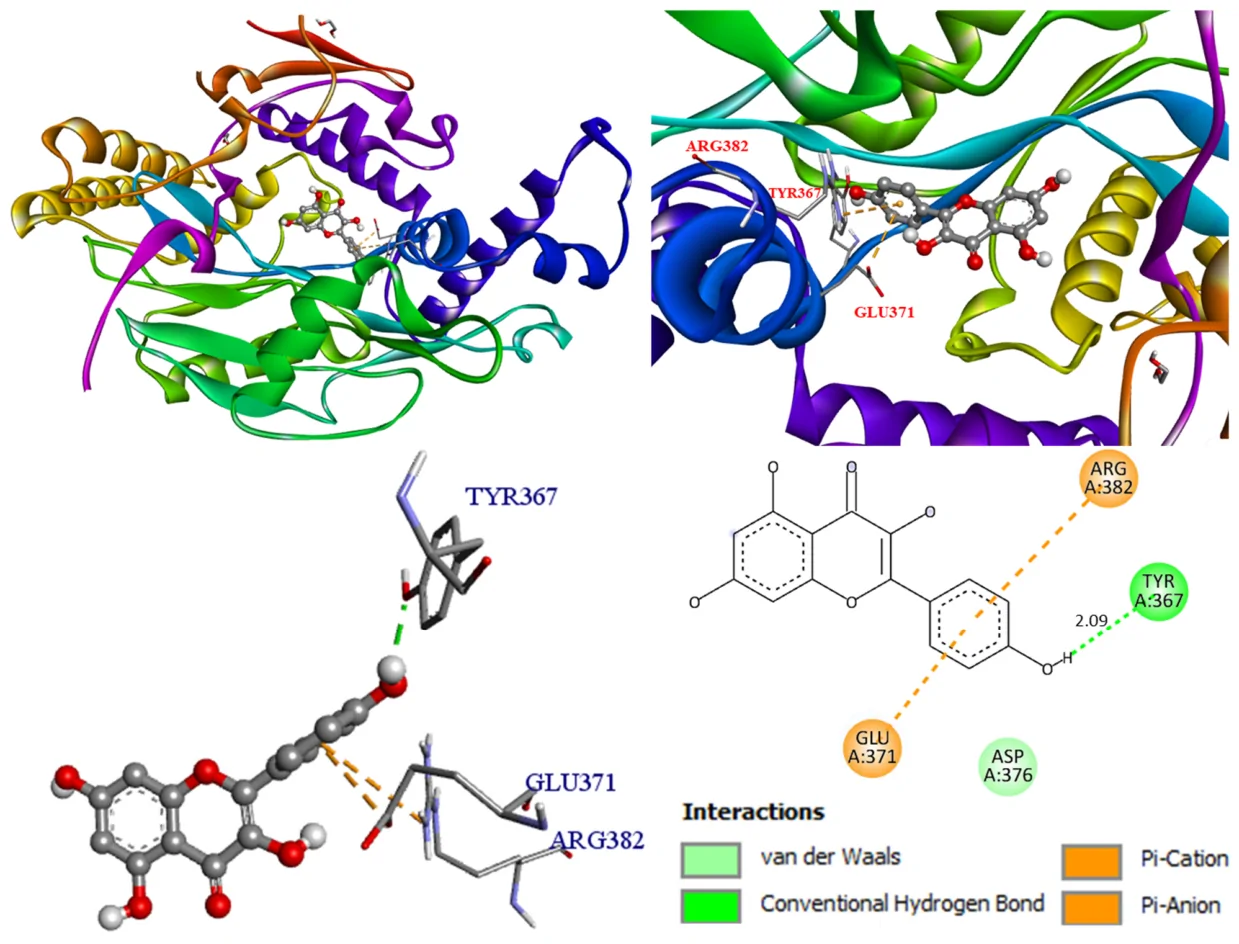
Molecular interactions of kaempferol within the active site of *Mus musculus* inducible nitric oxide synthase (iNOS) (PDB ID: 1M9T).

**Figure 10:**
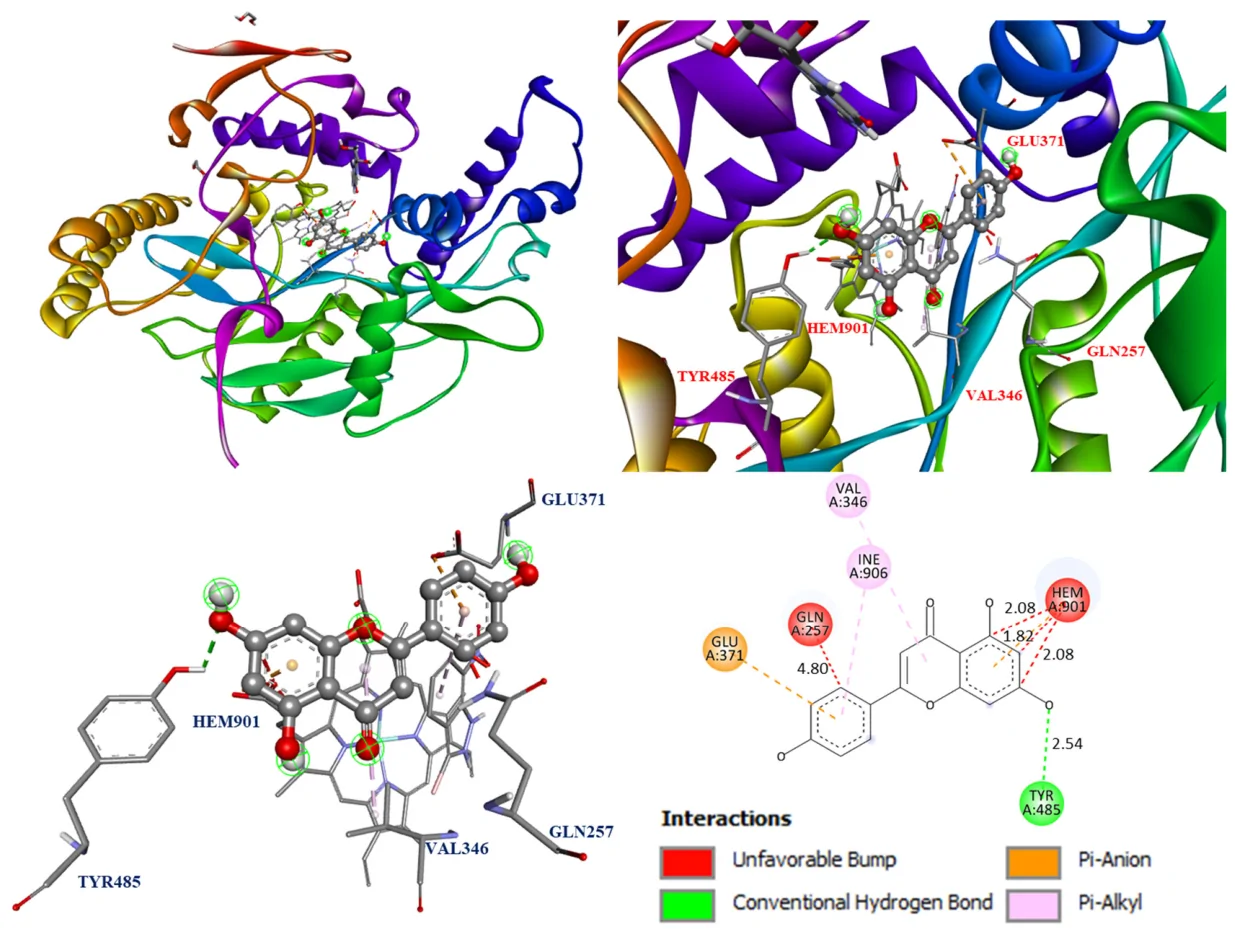
Molecular interactions of apigenin within the active site of *Mus musculus* inducible nitric oxide synthase (iNOS) (PDB ID: 1M9T).

The molecular docking results are presented in Figure 5, Figure 6, Figure 7, Figure 8, Figure 9 and Figure 10. Figure 5 demonstrates that quercetin, the primary bioactive compound in *P. aviculare*, interacts with α-glucosidase (PDB: 3A4A) by forming a conventional hydrogen bond with GLU277 (2.24 Å), engaging in *π-π* T-shaped stacking with TYR72, establishing *π*-alkyl interactions with VAL216, and exhibiting *π*-cation and *π*-anion contacts with ARG442 and ASP352/GLU411, respectively. Similarly, kaempferol (Figure 6) bound to the same enzyme through hydrogen bonds with ASP352 (2.02 Å) and ASP215 (1.97 Å) alongside *π*-alkyl interactions with VAL216 and *π-π* T-shaped stacking with PHE178 and TYR72. Positive control, acarbose, functions as a potent competitive inhibitor of α-glucosidase by structurally mimicking polysaccharide substrates, particularly at the catalytic site responsible for hydrolyzing α-1,4-glycosidic linkages. The critical structural distinction lies in the inter-ring linkage: while natural substrates utilize an oxygen-containing α-1,4-glycosidic bond (-O-), acarbose incorporates a nitrogen atom at this position, forming an isosteric but non-hydrolyzable N-glycosidic bond (-NH-). This nitrogen atom enhances binding affinity and prolongs inhibitor residence time within the enzyme’s active site primarily by enabling stronger hydrogen-bonding interactions with catalytic residues compared to the native oxygen linkage. Consequently, acarbose exhibits a markedly reduced dissociation rate, resulting in sustained active site occupancy and effective inhibition of oligosaccharide hydrolysis [52,53,54] (Figure 7). The predicted binding free energies of quercetin and kaempferol toward α-glucosidase were −8.6 and −8.5 kcal/mol, respectively (Table 8), consistent with the trend observed in their experimental IC_50_ values (Table 5). Given that the difference in docking scores between compounds was marginal (≤1 kcal/mol), these computational results are interpreted as broadly supportive of the experimental bioactivity data rather than as definitive predictors of relative potency. These predicted binding free energies were generally consistent with their experimentally observed inhibitory activities, suggesting a certain degree of correlation between the docking results and biological data. Nevertheless, the observed inhibitory activities are unlikely to be explained solely by the predicted binding energies, and the relationship between docking results and biological activity warrants further investigation. It is important to note that *S. cerevisiae* isomaltase (PDB: 3A4A) was utilized as the structural model in this study to ensure direct correlation and internal consistency with our in vitro biological assays, which evaluated inhibitory potential using α-glucosidase sourced from *S. cerevisiae*. While this computational model successfully corroborates the observed in vitro experimental trends, extrapolating these findings to human physiology represents a limitation. Notable structural and active site differences exist between the yeast enzyme and human intestinal maltase-glucoamylase (e.g., PDB: 3TOP). Therefore, further computational and experimental studies utilizing human α-glucosidase models are necessary to accurately validate the human therapeutic relevance of these bioactive flavonoids.

The interactions between bioactive compounds and *Mus musculus* iNOS (PDB: 1M9T) were analyzed using optimal docking poses. The active site of iNOS comprises four distinct binding pockets that facilitate its catalytic function, with the substrate-binding S pocket being particularly critical due to its incorporation of a heme cofactor (Fe-protoporphyrin IX), which serves as the primary docking target for ligand orientation and energy calculations [55]. The molecular docking model of quercetin, kaempferol, apigenin, and iNOS is shown in Figure 8, Figure 9 and Figure 10.

Integrated NO inhibition and Western blot analyses support the anti-inflammatory effects of quercetin and kaempferol through iNOS binding. Quercetin specifically forms a hydrogen bond with TYR367 (2.46 Å), a *π*-anion interaction with GLU371, and *π*-alkyl contacts with VAL346 and ILE906, as shown in Figure 8. Kaempferol forms a key hydrogen bond with TYR367 (2.09 Å) and exhibits *π*-cation and *π*-anion interactions with GLU371 and ARG382, along with van der Waals forces involving ASP376 (Figure 9). The positive control apigenin binds iNOS via *π*-anion interaction with GLU371 but displays destabilizing clashes with HEM901 (unfavorable bump distances at 1.82 Å, 1.92 Å, and 2.08 Å) (Figure 10).

As summarized in Table 8, quercetin (−8.6 kcal/mol) and kaempferol (−8.5 kcal/mol) exhibited strong binding affinities for α-glucosidase, both surpassing the positive control acarbose (−6.5 kcal/mol). These computational predictions align well with the in vitro α-glucosidase inhibitory trends previously reported in Table 5.

Separately, the docking analysis for iNOS (Table 9) revealed a similar pattern. Quercetin demonstrated the strongest binding affinity (−9.0 kcal/mol), followed by kaempferol (−8.6 kcal/mol), with both compounds outperforming the positive control apigenin (−7.6 kcal/mol). This in silico binding profile is consistent with the macrophage NO inhibition results presented in Table 7. Collectively, these results suggest that quercetin and kaempferol may represent promising dual-acting candidates for further exploration as natural α-glucosidase and iNOS inhibitors. However, it must be noted that standard molecular docking utilizes a rigid receptor model, which does not account for the dynamic conformational changes in the enzymes upon ligand binding under physiological conditions; thus, these computational findings warrant further functional validation.

### 3.11. In Silico Predicted Physicochemical Properties of Bioactive Compounds

Drug-likeness is a critical concept that aids in the development of viable drug candidates into marketable medicines [56]. To explore the potential applications of the bioactive compounds of *P. aviculare*, their physicochemical properties were predicted using MolSoft online web server. Key parameters, including lipophilicity (LogP), molecular weight (M.W.), number of hydrogen bond acceptors (HBA) and donors (HBD), drug-likeness score, polar surface area (PSA), and blood–brain barrier (BBB) penetration, were calculated and presented in Table 10. Additionally, Lipinski’s rule of five violations were determined to evaluate drug-likeness, while the oral absorption rate (%ABS) was computed to assess bioavailability [42].

Comprehensive in silico evaluation of the isolated compounds (Table 10) revealed that physicochemical properties and predicted drug-likeness are highly dependent on the structural class. The aglycones, including gallic acid, quercetin, isorhamnetin, kaempferide, and kaempferol, fully complied with Lipinski’s rule of five (zero violations), exhibiting optimal parameters (HBA ≤ 10, HBD ≤ 5, LogP ≤ 5, and M.W. ≤ 500). Extended ADME profiling supported these findings, highlighting their exceptional oral bioavailability potential, as evidenced by polar surface area (PSA) values below 140 Å^2^ and calculated absorption percentages (%ABS) exceeding 63% [56,57,58].

In contrast, the glycosylated derivatives, quercitrin, myricitrin, and avicularin, exhibited lower predicted oral absorption (51.47–57.61%) and accrued up to two Lipinski violations. These deviations are directly attributable to their appended sugar moieties, which significantly elevate the polar surface area (PSA > 150 Å^2^) and exceed the optimal threshold for hydrogen bond donors and acceptors, thereby restricting their capacity for passive transcellular diffusion. Similarly, the clinical α-glucosidase inhibitor acarbose displayed three Lipinski violations and the lowest predicted absorption rate (17.25%), driven by its exceptionally high PSA (265.95 Å^2^). However, this restricted systemic bioavailability represents a distinct pharmacological advantage rather than a limitation, as acarbose is designed to exert its inhibitory effects locally within the gastrointestinal lumen rather than through systemic circulation. In stark contrast, the high predicted systemic absorption (%ABS > 68%) of the active aglycone flavonoids (such as quercetin and kaempferol) underscores their superior potential for targeting peripheral or systemic inflammatory pathways.

In the MolSoft drug-likeness model, a positive score (greater than 0) indicates that a molecule contains structural properties commonly found in known commercial drugs. Notably, quantitative drug-likeness scoring identified compounds **2**, **3**, **5**, **6**, and **9** with robust positive scores ranging from 0.50 to 0.82, further corroborating their viability as promising candidates for oral drug development [59]. Blood–brain barrier (BBB) penetration scores were calculated using the BBB scoring model proposed by Gupta et al., in which values range from 0 (low permeability) to 6 (high permeability); scores < 4 are generally associated with limited CNS penetration and are therefore preferred for peripherally acting drug candidates intended to minimize central nervous system side effects [60]. All isolated compounds displayed BBB scores < 4, suggesting restricted CNS penetration and supporting their predicted suitability as non-CNS therapeutic agents targeting peripheral metabolic and inflammatory pathways [60].

The above results demonstrate that compounds **3** and **9** exhibit combined antioxidant, anti-inflammatory, and α-glucosidase inhibitory activities, alongside favorable predicted physicochemical properties from in silico analysis, suggesting their value as leads for further pharmacological investigation. While compliance with Lipinski’s rule of five and favorable PSA values serve as valuable preliminary indicators in early drug discovery, these parameters alone are insufficient to predict true oral bioavailability or therapeutic efficacy. Despite these limitations, the highly favorable drug-likeness profiles observed for these compounds suggest they warrant further investigation.

### 3.12. Structure–Activity Relationships

To systematically evaluate the influence of structural features on the observed biological activities, the isolated compounds were analyzed with respect to three principal structural determinants: glycosylation, hydroxylation pattern, and methylation (Figure 2).

Glycosylation consistently attenuated biological activity relative to the corresponding aglycone across all assays. In the α-glucosidase inhibition assay, rhamnose conjugates quercitrin and myricitrin exhibited the greatest potency reduction relative to their aglycones quercetin and myricetin, respectively, followed by the arabinose derivative avicularin, suggesting that the steric bulk and hydrophilicity of the sugar moiety modulate the degree of active site occlusion. The same trend was consistently observed across the antioxidant assays (DPPH, ABTS, FRAP, and superoxide), demonstrating that glycosylation reduces radical-scavenging capacity by shielding and reducing the number of free hydroxyl groups available for electron donation.

The hydroxylation pattern markedly influenced both antioxidant and enzyme inhibitory activities. In the α-glucosidase assay, myricetin (IC_50_ = 9.25 ± 0.17 μM), which bears an additional 5′-hydroxyl group on the B-ring relative to quercetin (IC_50_ = 13.4 ± 2.88 μM), demonstrated superior inhibitory potency, consistent with the capacity of additional hydroxyl substituents to form supplementary hydrogen bonds with active site residues Asp352 and Asp215. In the antioxidant assays, the *ortho*-dihydroxyl (catechol) B-ring configuration of quercetin and myricetin conferred superior radical-scavenging activity relative to monohydroxylated counterparts such as kaempferol, attributable to enhanced resonance stabilization of the resulting phenoxyl radical [61]. Furthermore, in the NO inhibition assay, the dihydroxylated B-ring of quercetin (IC_50_ = 5.04 ± 1.25 μM) was associated with stronger inhibitory activity than the monohydroxylated kaempferol (IC_50_ = 15.06 ± 1.50 μM), suggesting that the B-ring hydroxylation pattern also contributes to anti-inflammatory potency.

Methylation at C-4′ consistently impaired activity across all biological targets. This structural impact is most clearly demonstrated by comparing kaempferol and its 4′-*O*-methyl derivative kaempferide. In the α-glucosidase assay (Table 5), 4′-*O*-methylation resulted in an approximately 7-fold reduction in potency, with the IC_50_ value dropping from 4.50 ± 0.90 μM for kaempferol to 31.3 ± 3.90 μM for kaempferide, confirming that the free C-4′-OH is critical for hydrogen-bond donation to active site residues, as corroborated by the molecular docking results. A parallel attenuation was observed in the antioxidant and NO inhibition assays, where kaempferide consistently ranked among the weakest compounds. In contrast, C-3′ methylation in isorhamnetin retained partial antioxidant and NO inhibitory activity but impaired superoxide scavenging ability, indicating that the positional effect of methoxylation is more detrimental at C-4′ than at C-3′, likely owing to the greater electronic and steric influence of C-4′ substitution on B-ring electron density.

Beyond in vitro efficacy, structural modifications significantly dictated cellular biocompatibility and the therapeutic window. While the *ortho*-dihydroxy B-ring configuration of quercetin and the monohydroxylated B-ring of kaempferol conferred potent anti-inflammatory activity without inducing cellular damage, further functionalization compromised this safety profile. Specifically, the introduction of a third hydroxyl group on the B-ring in myricetin or the methylation of the C-4′ hydroxyl group in kaempferide resulted in notable cytotoxicity, with cell viability dropping below 80% at concentrations ≥ 50 μM. This indicates that while certain B-ring modifications may theoretically enhance target binding, they concurrently narrow the non-toxic therapeutic window in cellular models.

Finally, the structural determinants governing in vitro bioactivity are closely aligned with in silico pharmacokinetic predictions. The precise structural features that diminished target inhibition, notably glycosylation, simultaneously reduced predicted oral bioavailability and violated Lipinski’s parameters due to excessive hydrogen bonding capacity. Conversely, the aglycones quercetin and kaempferol, which demonstrated the optimal balance of unhindered B-ring hydroxyl groups for high potency and minimal cytotoxicity, also possessed the most favorable drug-likeness scores and predicted systemic absorption. However, while the introduction of a third hydroxyl group on the B-ring in myricetin theoretically enhances hydrogen-bond-driven enzyme inhibition and antioxidant capacity, this modification substantially increases the polar surface area (PSA = 118.09 Å^2^). Consequently, myricetin exhibits a lower predicted oral absorption rate (%ABS = 68.26%) compared to the dihydroxylated quercetin (%ABS = 73.60%), highlighting the delicate balance between maximizing target binding and maintaining optimal systemic bioavailability.

In summary, this comprehensive structure–activity relationship analysis demonstrates that a highly specific structural triad determines the multi-target bioactivity of *P. aviculare* flavonoids. First, the aglycone skeleton is crucial; the absence of a bulky glycosidic moiety avoids steric hindrance within enzyme active sites (e.g., α-glucosidase and iNOS), which not only enhances in vitro inhibitory potency but also correlates directly with favorable in silico predictions, including an optimal topological polar surface area (PSA < 140 Å^2^) and improved predicted systemic absorption (%ABS > 68%). Second, B-ring hydroxylation is a key regulatory factor influencing efficacy and safety. The *ortho*-dihydroxy configuration (e.g., quercetin) maximizes free radical scavenging capacity and target binding affinity, while monohydroxylated B-rings (e.g., kaempferol) exhibit excellent biocompatibility, with no cytotoxicity observed at any tested concentrations. Finally, maintaining the free hydroxyl group at the C-4′ position is essential. Methylation at this site (e.g., kaempferide) severely disrupts the hydrogen-bond-driven enzyme inhibition necessary for antidiabetic and anti-inflammatory efficacy. Ultimately, this specific structural arrangement relies on an aglycone core, controlled B-ring hydroxylation, and an unhindered C-4′ hydroxyl group to determine the precise balance between maximizing pleiotropic pharmacological activity and maintaining both the cellular safety and pharmacokinetic viability of the lead compounds.

## 4. Conclusions

This study provides a comprehensive phytopharmacological characterization of the aerial parts of *P. aviculare*, identifying quercetin and kaempferol as potent, multi-target bioactive leads. We demonstrate that these flavonoids exert concurrent antioxidant, α-glucosidase inhibitory, and anti-inflammatory activities, with structure–activity relationships highlighting the negative impact of glycosylation on pharmacological efficacy. In the cellular model, the attenuation of LPS-induced inflammation by kaempferol was associated with the down-regulation of pro-inflammatory signaling (p38 MAPK) and an increase in M2 macrophage polarization markers (KLF4 and Arg-1). Importantly, this study provides a comprehensive evaluation of the dual α-glucosidase inhibitory and anti-inflammatory potential of *P. aviculare* through bioactivity-guided isolation, cellular validation, and molecular docking analyses, thereby expanding the current phytopharmacological evidence for this medicinal plant. Supported by molecular docking affinities and favorable in silico drug-likeness profiles, these findings position compounds **3** and **9** as promising multifunctional agents for managing free radical damage and metabolic inflammation. However, the scope of this study is limited to in vitro and computational models. Future research should prioritize in vivo validation using streptozotocin (STZ)-induced diabetes models to evaluate antidiabetic efficacy, and dextran sulfate sodium (DSS)-induced colitis models to validate systemic anti-inflammatory effects and the functional involvement of macrophage polarization.

## Figures and Tables

**Table 1:** TPC and TFC of the aerial part of *P. aviculare* from different solvents.

Extracting Solvents	Relative Polarity	TPC (mg/g) ^a^ (GAE)	TFC (mg/g) ^b^ (QE)
*n*-Hexane	0.009	0.16 ± 0.05	7.52 ± 0.86
CH_2_Cl_2_	0.269	12.76 ± 2.14	52.00 ± 3.92
EtOAc	0.288	47.17 ± 3.79	44.06 ± 5.88
Acetone	0.355	55.65 ± 6.06	71.08 ± 6.64
EtOH	0.654	74.49 ± 9.06	41.26 ± 3.44
MeOH	0.762	80.27 ± 4.66	72.98 ± 7.42
Water	1.000	28.34 ± 2.14	10.42 ± 1.05
Water 100 °C	1.000	102.91 ± 12.79	62.18 ± 4.18

^a^ TPC expressed as gallic acid equivalents (GAE) in milligrams per gram of extract (mg GAE/g). ^b^ TFC reported as quercetin equivalents (QE) in milligrams per gram of extract (mg QE/g).

**Table 2:** The antioxidant effects of the aerial part of *P. aviculare* extract.

Extracting Solvents	SC_50_ (μg/mL) ^a^			TE (mM/g) ^b^
	DPPH	ABTS	Superoxide	FRAP
*n*-Hexane	>400	>400	>400	82.35 ± 2.34 ***
CH_2_Cl_2_	>400	314.46 ± 2.15 ***	>400	197.17 ± 4.79 ***
EtOAc	248.78 ± 6.53 **	152.53 ± 3.52 ***	>400	449.96 ± 5.82 ***
Acetone	166.16 ± 6.57 *	>400	>400	402.05 ± 2.19 ***
EtOH	134.43 ± 2.06 *	47.50 ± 0.92 **	>400	604.22 ± 6.50 ***
MeOH	77.04 ± 5.99 **	49.42 ± 1.21 **	>400	576.12 ± 7.15 ***
Water	>400	146.99 ± 5.07 ***	104.92 ± 8.12 ***	374.94 ± 3.50 ***
Water 100 °C	100.76 ± 7.08	36.04 ± 0.46 **	56.34 ± 3.47 **	651.23 ± 6.53 ***
BHT ^c^	107.06 ± 4.63	19.02 ± 1.50	–	4232.67 ± 32.53
Cynaroside ^c^	–	–	13.98 ± 1.28	–

^a^ SC_50_ values were expressed as 50% scavenging concentration (mean ± SD, *n* = 3); ^b^ FRAP values were expressed as Trolox equivalents per gram of extract (mM TE/g); ^c^ BHT and cynaroside were served as positive controls; * *p* < 0.05, ** *p* < 0.01, and *** *p* < 0.001 compared with the control.

**Table 3:** The antioxidant effects of isolated compounds from *P. aviculare*.

Compounds	SC_50_ (μM) ^a^			TE (mM/g) ^b^
	DPPH	ABTS	Superoxide	FRAP
Gallic acid (**1**)	3.37 ± 0.82 ***	6.46 ± 0.60 ***	39.87 ± 3.25 *	9871.37 ± 42.34 ***
Quercitrin (**2**)	9.57 ± 1.09 ***	17.31 ± 2.11	91.47 ± 11.87 **	3752.46 ± 23.68 **
Quercetin (**3**)	4.31 ± 0.51 ***	7.89 ± 0.83 **	30.67 ± 3.30 *	25,511.50 ± 88.61 ***
Myricetin (**4**)	3.55 ± 0.48 ***	9.62 ± 1.77 **	35.54 ± 5.01 *	15,243.65 ± 39.74 ***
Myricitrin (**5**)	28.41 ± 3.48 *	17.40 ± 2.82	41.63 ± 3.98 **	3497.19 ± 21.92 **
Avicularin (**6**)	6.50 ± 0.91 ***	12.44 ± 2.53 *	35.93 ± 1.69 *	3838.15 ± 23.66 ***
Isorhamnetin (**7**)	4.60 ± 0.32 ***	23.53 ± 1.48 **	>100	15,026.44 ± 63.13 ***
Kaempferide (**8**)	59.15 ± 6.55 *	33.44 ± 2.75 **	>100	5147.44 ± 31.59 *
Kaempferol (**9**)	31.23 ± 4.09	10.63 ± 1.96 *	>100	5032.79 ± 25.72 *
BHT ^c^	36.99 ± 4.54	17.36 ± 3.14	–	4561.78 ± 12.93
Cynaroside ^c^	–	–	20.52 ± 2.87	–

^a^ SC_50_ values were expressed as 50% scavenging concentration (mean ± SD, *n* = 3); ^b^ FRAP values were expressed as Trolox equivalents per gram of extract (mM TE/g); ^c^ BHT and cynaroside were served as positive controls; * *p* < 0.05, ** *p* < 0.01, and *** *p* < 0.001 compared with the control.

**Table 4:** Effects of various solvent extracts on the inhibition of α-glucosidase activity.

Extracting Solvents	IC_50_ (μg/mL) ^a^
*n*-Hexane	575.90 ± 17.76 **
CH_2_Cl_2_	453.42 ± 21.69 *
EtOAc	195.01 ± 4.86 **
Acetone	162.99 ± 14.91 **
EtOH	138.62 ± 16.93 ***
MeOH	210.70 ± 17.46 *
Water	396.59 ± 22.75
Water at 100 °C	187.67 ± 12.98 **
Acarbose ^b^	368.67 ± 21.40

^a^ IC_50_ values (mean ± SD, *n* = 3) represent the concentration required for 50% inhibition; ^b^ Acarbose was served as positive control; * *p* < 0.05, ** *p* < 0.01, and *** *p* < 0.001 compared with the control.

**Table 5:** Bioactive compounds from *P. aviculare* with α-glucosidase inhibition.

Compounds	IC_50_ (μM) ^a^
Gallic acid (**1**)	377.3 ± 49.32 **
Quercitrin (**2**)	473.5 ± 46.68 *
Quercetin (**3**)	13.4 ± 2.88 ***
Myricetin (**4**)	9.25 ± 0.17 ***
Myricitrin (**5**)	>800
Avicularin (**6**)	206.3 ± 29.69 **
Isorhamnetin (**7**)	33.9 ± 2.17 ***
Kaempferide (**8**)	31.3 ± 3.90 ***
Kaempferol (**9**)	4.5 ± 0.90 ***
Acarbose ^b^	519.7 ± 18.67

^a^ IC_50_ values (mean ± SD, *n* = 3) represent the concentration required for 50% inhibition; ^b^ Acarbose as positive control; * *p* < 0.05, ** *p* < 0.01, and *** *p* < 0.001 compared with the control.

**Table 6:** Inhibitory effects of *P. aviculare* solvent extracts on LPS-induced NO production in RAW 264.7 macrophages.

Extracting Solvents	IC_50_ (μg/mL) ^a^
*n*-Hexane	31.37 ± 3.69 **
CH_2_Cl_2_	34.47 ± 2.76 **
EtOAc	33.81 ± 3.69 **
Acetone	28.57 ± 3.18 **
EtOH	30.69 ± 3.84 **
MeOH	31.04 ± 1.89 **
Water	57.16 ± 6.92 **
Water at 100 °C	54.37 ± 6.48 **
Apigenin ^b^	5.14 ± 0.96

^a^ IC_50_ values (mean ± SD, *n* = 3) represent the concentration required for 50% inhibition; ^b^ Apigenin served as the positive control; ** *p* < 0.01 compared with the control.

**Table 7:** Effects of isolated compounds on nitric oxide (NO) production in RAW 264.7 cells.

Compounds	IC_50_ (μM) ^a^
Gallic acid (**1**)	12.60 ± 2.03 *
Quercitrin (**2**)	>100
Quercetin (**3**)	5.04 ± 1.25 **
Myricetin (**4**)	N/A ^b^
Myricitrin (**5**)	>100
Avicularin (**6**)	23.47 ± 3.68 *
Isorhamnetin (**7**)	15.50 ± 2.22
Kaempferide (**8**)	N/A ^b^
Kaempferol (**9**)	15.06 ± 1.50
Apigenin ^c^	16.08 ± 1.86

^a^ IC_50_ values (mean ± SD, *n* = 3) represent the concentration required for 50% inhibition; ^b^ Not applicable due to cytotoxicity-induced artifacts (cell viability < 80%); ^c^ Apigenin served as positive control; * *p* < 0.05 and ** *p* < 0.01 compared with the control.

**Table 8:** Binding energies of bioactive components and acarbose against α-glucosidase.

Compounds	Affinity (kcal/mol)
Quercetin (**3**)	−8.6
Myricetin (**4**)	−8.0
Kaempferol (**9**)	−8.5
Acarbose ^a^	−6.5

^a^ Acarbose served as a positive control.

**Table 9:** Binding energies of bioactive compounds and apigenin against iNOS.

Compounds	Affinity (kcal/mol)
Gallic acid (**1**)	−7.8
Quercetin (**3**)	−9.0
Myricetin (**4**)	−8.1
Avicularin (**6**)	−7.7
Isorhamnetin (**7**)	−7.8
Kaempferide (**8**)	−7.4
Kaempferol (**9**)	−8.6
Apigenin ^a^	−7.6

^a^ Apigenin served as a positive control.

**Table 10:** Computational prediction of physicochemical properties.

Compound	M.W.	LogP	HBA	HBD	Drug-Likeness Score	%ABS ^a^	PSA (Å^2^)	BBB ^b^	Lipinski’s Violation
Gallic acid (**1**)	170.12	0.78	5	4	−0.22	82.76	77.22	2.52	0
Quercitrin (**2**)	448.38	0.32	11	7	0.82	57.61	150.41	1.66	2
Quercetin (**3**)	302.24	1.19	7	5	0.52	73.60	102.61	2.55	0
Myricetin (**4**)	318.24	0.97	8	6	−0.24	68.26	118.09	2.32	1
Myricitrin (**5**)	464.38	0.10	12	8	0.67	51.47	165.88	1.61	2
Avicularin (**6**)	434.36	0.34	11	7	0.60	56.86	152.57	1.70	2
Isorhamnetin (**7**)	316.26	1.34	7	4	0.39	76.08	93.69	2.66	0
Kaempferide (**8**)	300.26	2.14	6	3	0.27	82.91	77.06	2.91	0
Kaempferol (**9**)	286.24	1.61	6	4	0.50	78.94	87.13	2.78	0
Apigenin	270.24	3.22	5	3	0.39	83.92	73.57	2.97	0
Acarbose	645.25	−4.58	19	14	0.40	17.25	265.95	0.81	3

^a^ %ABS calculated via 109 − (0.345 × PSA). ^b^ BBB scores: 0 (low) to 6 (high) permeability; ≤4 preferred for non-CNS drugs.

## Data Availability

Data is contained within the article and Supplementary Material. Publicly available datasets were analyzed in this study. The crystal structures of *Saccharomyces cerevisiae* isomaltase (PDB ID: 3A4A) and *Mus musculus* iNOS (PDB ID: 1M9T) can be found in the Protein Data Bank (https://www.rcsb.org/structure/3A4A and https://www.rcsb.org/structure/1M9T, respectively; accessed on 25 February 2026). The occurrence record data for *Polygonum aviculare* can be found in the Global Biodiversity Information Facility (GBIF) database (https://doi.org/10.15468/dL.46f4nm; accessed on 25 February 2026).

## Supplementary Materials

The following supporting information can be downloaded at https://www.mdpi.com/article/10.3390/antiox15080947/s1: Figures S1–S3: Spectroscopic data of compound **1**; Figures S4–S6: Spectroscopic data of compound **2**; Figures S7–S9: Spectroscopic data of compound **3**; Figures S10–S12: Spectroscopic data of compound **4**; Figures S13–S15: Spectroscopic data of compound **5**; Figures S16–S18: Spectroscopic data of compound **6**; Figures S19–S21: Spectroscopic data of compound **7**; Figures S22–S24: Spectroscopic data of compound **8**; Figures S25–S27: Spectroscopic data of compound **9**; Figure S28: Major compounds of *P. aviculare* and positive control apigenin on MTT assay using RAW264.7 cell line; Figure S29: HPLC Chromatographic Conditions; Figure S30: Validation of the molecular docking RMSD.

## Abbreviations

%ABS	Oral absorption rate
ABTS	2,2′-Azino-bis(3-ethylbenzothiazoline-6-sulfonic acid)
ADME	Absorption, distribution, metabolism, and excretion
Arg-1	Arginase-1
BBB	Blood–brain barrier
BH-4	Tetrahydrobiopterin
BHT	Butylated hydroxytoluene
BSA	Bovine serum albumin
CC	Column chromatography
CNS	Central nervous system
COX-2	Cyclooxygenase-2
DMEM	Dulbecco’s Modified Eagle Medium
DPBS	Dulbecco’s phosphate-buffered saline
DPPH	2,2-Diphenyl-1-picrylhydrazyl
DSS	Dextran sulfate sodium
DTT	Dithiothreitol
EDTA	Ethylenediaminetetraacetic acid
ESI-MS	Electrospray ionization mass spectrometry
FAD	Flavin adenine dinucleotide
FBS	Fetal bovine serum
FC	Folin–Ciocalteu
FMN	Flavin mononucleotide
FRAP	Ferric reducing antioxidant power
GAE	Gallic acid equivalent
GBIF	Global Biodiversity Information Facility
HBA	Hydrogen bond acceptor
HBD	Hydrogen bond donor
HEPES	4-(2-Hydroxyethyl)-1-piperazineethanesulfonic acid
I.D.	Internal diameter
IC_50_	Half maximal inhibitory concentration
IκBα	Inhibitor of nuclear factor kappa B alpha
iNOS	Inducible nitric oxide synthase
IR	Infrared ray
KLF4	Krüppel-like factor 4
LogP	Lipophilicity
LPS	Lipopolysaccharide
M.W.	Molecular weight
MAPK	Mitogen-activated protein kinase
MTT	3-(4,5-Dimethylthiazol-2-yl)-2,5-diphenyltetrazolium bromide
NADH	Nicotinamide adenine dinucleotide
NADPH	Nicotinamide adenine dinucleotide phosphate
NBT	Nitroblue tetrazolium
NCDs	Non-communicable diseases
NF-κB	Nuclear factor-kappa B
NMR	Nuclear magnetic resonance
NO	Nitric oxide
NSAIDs	Non-steroidal anti-inflammatory drugs
*p*-NPG	*p*-Nitrophenyl-α-D-glucopyranoside
PAGE	Polyacrylamide gel electrophoresis
PDB	Protein Data Bank
PMS	Phenazine methosulfate
PSA	Polar surface area
PTLC	Preparative thin-layer chromatography
PVDF	Polyvinylidene fluoride
QE	Quercetin equivalent
RIPA	Radioimmunoprecipitation assay buffer
RMSD	Root-mean-square deviation
*R_f_*	Retention factor
ROS	Reactive oxygen species
RT	Room temperature
SAR	Structure–activity relationship
SC_50_	Half maximal scavenging concentration
SD	Standard deviation
SDS	Sodium dodecyl sulfate
STZ	Streptozotocin
TBST	Tris-buffered saline with Tween 20
TCM	Traditional Chinese Medicine
TE	Trolox equivalent
TEMED	Tetramethylethylenediamine
TFC	Total flavonoid content
TLC	Thin-layer chromatography
TPC	Total phenolic content
TPTZ	2,4,6-Tripyridyl-s-triazine
UV	Ultraviolet
WHO	World Health Organization
