# Supplementary material for: Comprehensive Phytopharmacological Profiling of Polygonum aviculare L.: Antioxidant, Anti-Inflammatory, and α-Glucosidase Inhibitory Activities of Bioactive Constituents with Molecular Docking and In Silico Drug-Likeness Analysis
Source: Antioxidants (Basel). 2026 Jul 29;15(8):947. doi: 10.3390/antiox15080947 (PMC13509247; doi:10.3390/antiox15080947)
Supplement: Supplementary file 1 [file antioxidants-15-00947-s001.zip › antioxidants-4415532-supplementary.pdf]

Supporting Information

Comprehensive Phytopharmacological Profiling of *Polygonum aviculare* L.: Antioxidant, Anti-inflammatory, and α-Glucosidase Inhibitory Activities of Bioactive Constituents with Molecular Docking and *In silico* Drug-likeness Analysis

Yuen-Sing Lee [a], Sin-Min Li [b], Hui-Ju Chen [c], and Jih-Jung Chen [a,b,d,e,*]

[a] Biomedical Industry Ph.D. Program, College of Life Sciences, National Yang Ming Chiao Tung University, Taipei 112304, Taiwan

[b] Department of Pharmacy, School of Pharmaceutical Sciences, National Yang Ming Chiao Tung University, Taipei 112304, Taiwan

[c] College of Management, Executive Master of Business Administration (EMBA), National Yang Ming Chiao Tung University, Taipei 112304, Taiwan

[d] Traditional Herbal Medicine Research Center, Taipei Medical University Hospital, Taipei 110301, Taiwan

[e] Department of Medical Research, China Medical University Hospital, China Medical University, Taichung 404333, Taiwan

* Corresponding author

E-mail address: jjungchen@nycu.edu.tw Tel.: +886-2-2826-7195; Fax: +886-2-2823-2940

Contents

Spectrum data of isolated compounds……S3

MS, IR, and NMR spectrum of isolated compound **1**……S5

MS, IR, and NMR spectrum of isolated compound **2**……S6

MS, IR, and NMR spectrum of isolated compound **3**……S8

MS, IR, and NMR spectrum of isolated compound **4**……S9

MS, IR, and NMR spectrum of isolated compound **5**……S11

MS, IR, and NMR spectrum of isolated compound **6**……S12

MS, IR, and NMR spectrum of isolated compound **7**……S14

MS, IR, and NMR spectrum of isolated compound **8**……S15

MS, IR, and NMR spectrum of isolated compound **9**……S17

MTT assay with RAW264.7 cell line……S18

HPLC Chromatographic Conditions……S19

Validation of the molecular docking RMSD……S20

Spectrum data of isolated compounds

Gallic acid (**1**): white powder; UV (MeOH) λ_{max} (log ε) 271 (4.1), 217 (3.8) nm; IR (neat) ν_{max} 3493, 3271 (OH), 1666 (carboxylic acid C=O stretch), 1645, 1606, 1539 (conjugated aromatic C=C stretch) cm^{-1}; ^{1}H-NMR (400 MHz, acetone-d_6) δ 8.57 (3H, br s, OH-3, OH-4, OH-5), 7.14 (2H, s, H-2, H-6); ESI-MS *m/z* 169 $[M–H]^{-}$.

Quercitrin (**2**): yellow powder; $[\alpha]^{25}_{D}$ = +78.3° (*c* 0.1, MeOH); UV (MeOH) λ_{max} (log ε) 256 (4.2), 352 (3.9) nm; IR (neat) ν_{max} 3242 (OH), 1651 (conjugated ketone C=O stretch), 1597, 1573 (aromatic C=C stretch) cm^{-1}; ^{1}H-NMR (600 MHz, CD_3OD) δ 7.35 (1H, d, *J* = 1.7 Hz, H-2'), 7.31 (1H, dd, *J* = 1.7 Hz, H-6'), 6.94 (1H, d, *J* = 8.3 Hz, H-5'), 6.40 (1H, d, *J* = 1.7 Hz, H-8), 6.22 (1H, d, *J* = 1.8 Hz, H-6), 5.33 (1H, d, H-1''), 4.23 (1H, dd, *J* = 3.4, 1.7 Hz, H-2''), 3.76 (1H, dd, *J* = 9.4, 3.4 Hz, H-3''), 3.40 (1H, dq, *J* = 6.0, 3.4 Hz, H-5''), 3.35 (1H, dd, *J* = 9.4, 3.4 Hz, H-4''), 0.93 (3H, d, *J* = 6.0 Hz, H-6'); ESI-MS *m/z* 434 $[M–H]^{-}$.

Quercetin (**3**): yellow powder; UV (MeOH) λ_{max} 257 (4.5), 369 (4.3) nm; IR (neat) ν_{max} 3390, 3265 (OH), 1646 (conjugated ketone C=O stretch), 1604, 1517 (aromatic rings C=C stretch) cm^{-1}; ^{1}H-NMR (400 MHz, acetone-d_6): δ 12.17 (1H, s, OH-5), 9.70 (1H, s, OH-7), 8.53 (1H, s, OH-4'), 8.37 (1H, s, OH-3'), 8.02 (1H, s, OH-3), 7.83 (1H, d, *J* = 2.3 Hz, H-2'), 7.70 (1H, dd, *J* = 8.5, 2.3, H-6'), 6.99 (1H, d, *J* = 8.5, H-5'), 6.52 (1H, d, *J* = 2.2 Hz, H-8), 6.26 (1H, d, *J* = 2.2 Hz, H-6); ESI-MS *m/z* 300 $[M–H]^{-}$.

Myricetin (**4**): yellow powder; UV (MeOH) λ_{max} (log ε) 254 (4.4), 372 (4.2) nm; IR (neat) ν_{max} 3418, 3271 (OH), 1661 (conjugated ketone C=O stretch), 1618, 1605, 1593, 1518 (aromatic C=C stretch) cm^{-1}; ^{1}H-NMR (600 MHz, CD_3OD) δ 7.34 (1H, d, *J* = 2.3 Hz, H-2'), 7.34 (1H, d, *J* = 2.3, H-6'), 6.40 (1H, d, *J* = 2.2 Hz, H-8), 6.20 (1H, d, *J* = 2.2 Hz, H-6); ESI-MS *m/z* 317 $[M–H]^{-}$.

Myricitrin (**5**): yellow powder; $[\alpha]^{25}_{D}$ = +62.5 ° (*c* 0.1, MeOH); UV (MeOH) λ_{max} 265 (4.0), 343 (3.7) nm; IR (neat) ν_{max} 3288 (OH), 1651 (conjugated ketone C=O stretch), 1600, 1575, 1552 (aromatic C=C stretch); ^{1}H-NMR (600 MHz, CD_3OD) δ 6.95 (2H, s, H-2', H-6'), 6.38 (1H, d, *J* = 2.0 Hz, H-8), 6.21 (1H, d, *J* = 2.0 Hz, H-6), 5.30 (1H, d, *J* = 1.5 Hz, H-1''), 4.23 (1H, d, *J* = 3.3 Hz, H-2''), 3.79 (1H, dd, *J* = 9.5, 3.3 Hz, H-3''), 3.50 (1H, m, H-5''), 3.34 (1H, t, *J* = 9.5 Hz, H-4''), 0.96 (3H, d, *J* = 6.2 Hz, H-6''); ESI-MS *m/z* 463 $[M–H]^{-}$.

Avicularin (**6**): yellow powder; $[\alpha]^{25}_{D}$ = +49.8° (*c* 0.1, MeOH); UV (MeOH) λ_{max} 258 (4.3), 355 (4.0) nm; IR (neat) ν_{max} 3248 (OH), 1647 (conjugated ketone C=O stretch), 1604, 1550, 1529, 1500 (aromatic C=C stretch) cm^{-1}; ^{1}H-NMR (400 MHz, CD_3OD) δ 7.53 (1H, d, *J* = 2.1 Hz, H-2'), 7.48 (1H, dd, *J* = 8.4, 2.1 Hz, H-6'), 6.90 (1H, d, *J* = 8.4 Hz, H-5'), 6.39 (1H, d, *J* = 2.0 Hz, H-8), 6.21 (1H, d, *J* = 2.1 Hz, H-6), 5.47 (1H, s, H-1''), 4.35 (1H, d, *J* = 2.1 Hz, H-2''), 3.93 (1H, m, H-3''), 3.89 (1H, m, H-4''), 3.51 (2H, m, H-5''); ESI-MS *m/z* 435 $[M+H]^+$.

Isorhamnetin (**7**): white powder; UV (MeOH) λ_{max} (log ε) 255 (4.2), 366 (3.9) nm; IR (neat) ν_{max} 3269 (OH), 1651 (conjugated ketone C=O stretch), 1614, 1597, 1556, 1502 (aromatic C=C stretch) cm^{-1}; ^{1}H-NMR (600 MHz, acetone-d_6) δ 12.16 (1H, brs, 5-OH), 9.67 (1H, s, 7-OH), 8.31 (1H, s, 4'-OH), 8.05 (1H, s, 3-OH), 7.90 (1H, d, *J* = 2.0 Hz, H-2'), 7.82 (1H, dd, *J* = 8.5, 2.0 Hz, H-6'), 7.01 (1H, d, *J* = 8.5 Hz, H-5'), 6.56 (1H, d, *J* = 1.9 Hz, H-8), 6.28 (1H, d, *J*= 1.9 Hz, H-6), 3.94 (3H, s, 3'-OMe); ESI-MS *m/z* 315 $[M–H]^-$.

Kaempferide (**8**): white powder; UV (MeOH) λ_{max} (log ε) 266 (4.1), 327 (3.8) nm; IR (neat) ν_{max} 3518, 3462 (free OH), 3277 (hydrogen-bonded OH), 1652 (conjugated ketone C=O stretch), 1612, 1556, 1508 (aromatic C=C stretch) cm^{-1}; ^{1}H-NMR (600 MHz, acetone-d_6) δ 12.15 (1H, s, 5-OH), 9.70 (1H, s, 7-OH), 8.23 (2H, dt, *J* = 9.0, 2.1 Hz, H-2', H-6'), 8.08 (1H, s, 3-OH), 7.14 (2H, dt, *J* = 9.0, 2.1 Hz, H-3', H-5'), 6.55 (1H, d, *J* = 2.0 Hz, H-8), 6.28 (1H, d, *J* = 2.0 Hz, H-6), 3.90 (3H, s, 4'-OMe); ESI-MS *m/z* 299 $[M–H]^-$.

Kaempferol (**9**): white powder; UV (MeOH) λ_{max} 265 (4.6), 365 (4.4) nm; IR (neat) ν_{max} 3304, 3194 (OH), 1657 (conjugated ketone C=O stretch), 1609, 1566, 1503 (aromatic C=C stretch) cm^{-1}; ^{1}H-NMR (600 MHz, acetone-d_6) δ 12.17 (1H, s, 5-OH), 9.68 (1H, s, 7-OH), 9.06 (1H, s, 4'-OH), 8.16 (2H, dt, *J* = 8.8, 2.3 Hz, H-2', H-6'), 8.02 (1H, s, 3-OH), 7.02 (2H, dt, *J* = 8.8, 2.3 Hz, H-3', H-5'), 6.55 (1H, d, *J* = 2.2 Hz, H-8), 6.28 (1H, d, *J* = 2.2 Hz, H-6); ESI-MS *m/z* 285 $[M–H]^-$.

MS, IR, and NMR spectrum of isolated compounds

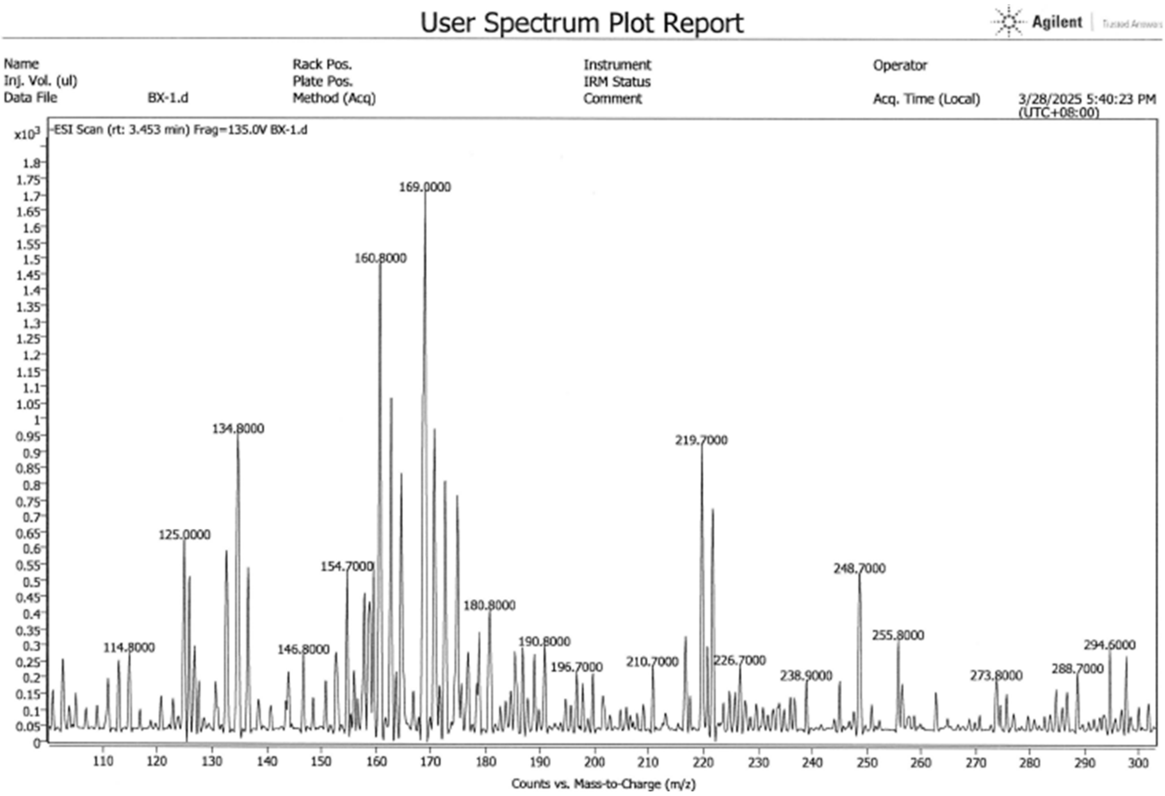

Figure S1. ESI-MS spectrum of gallic acid (**1**).

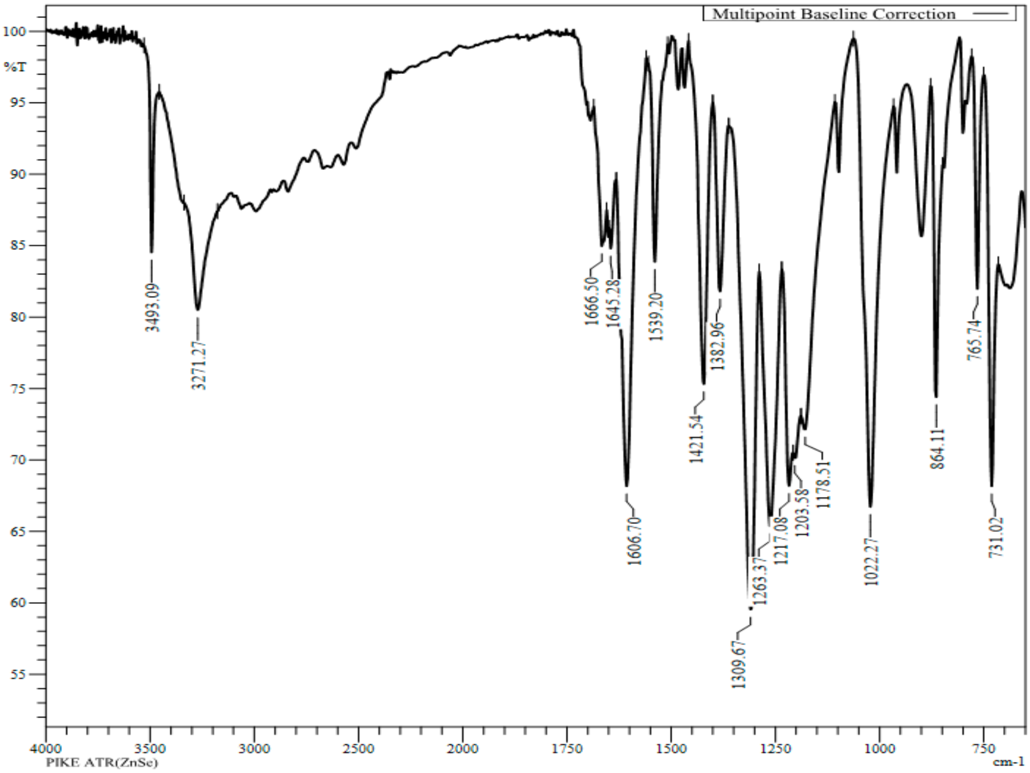

Figure S2. IR spectrum of gallic acid (**1**).

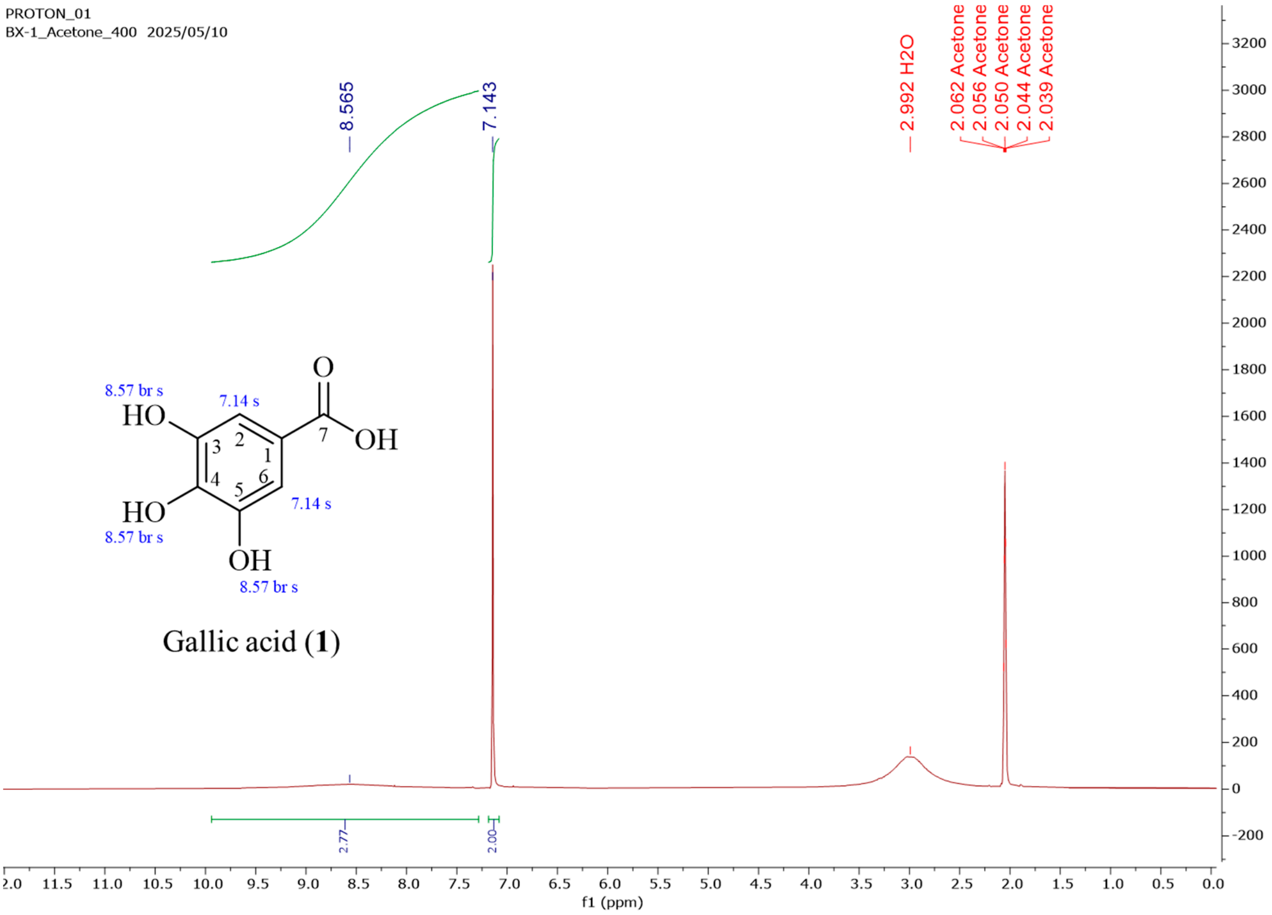

Figure S3. ^{1}H-NMR spectrum of gallic acid (**1**).

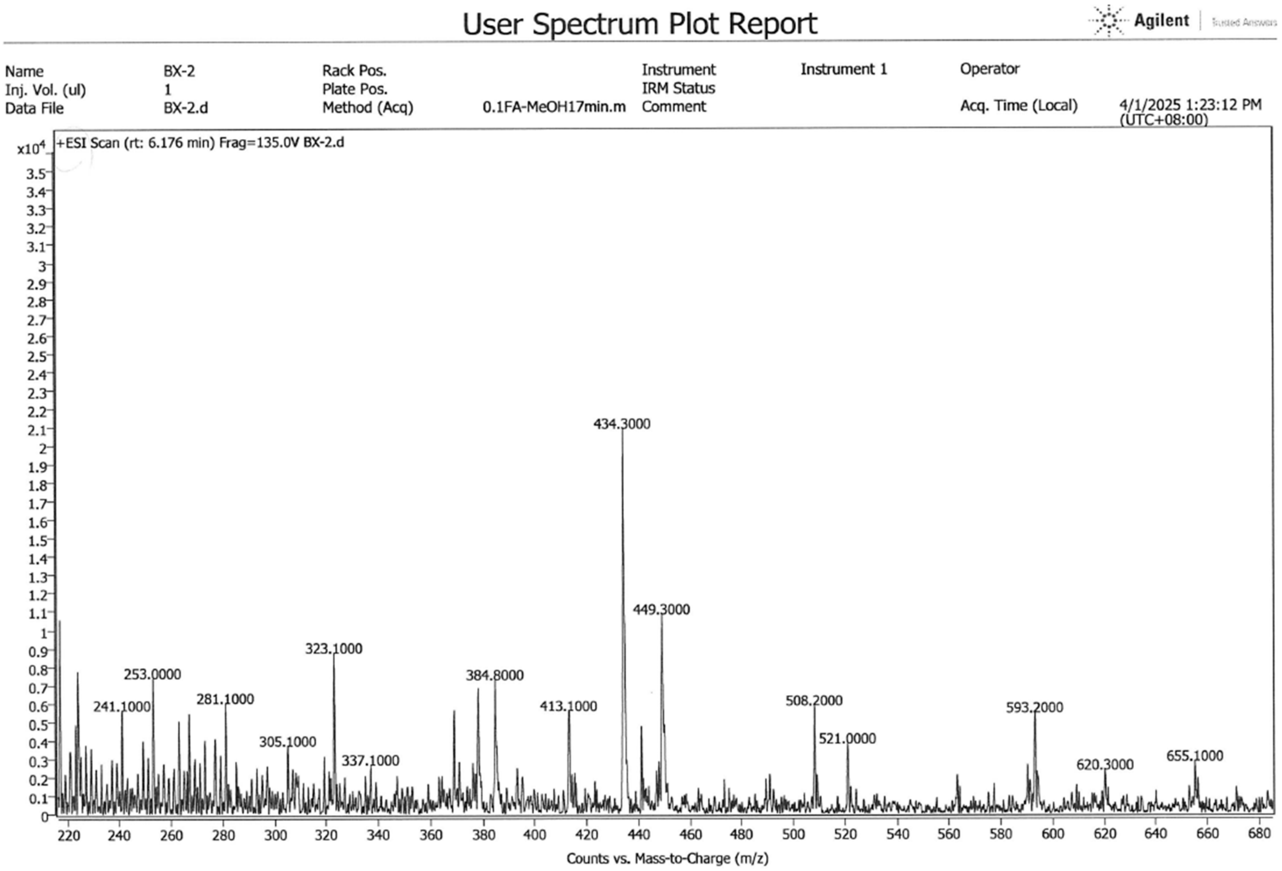

Figure S4. ESI-MS spectrum of quercitrin **(2)**.

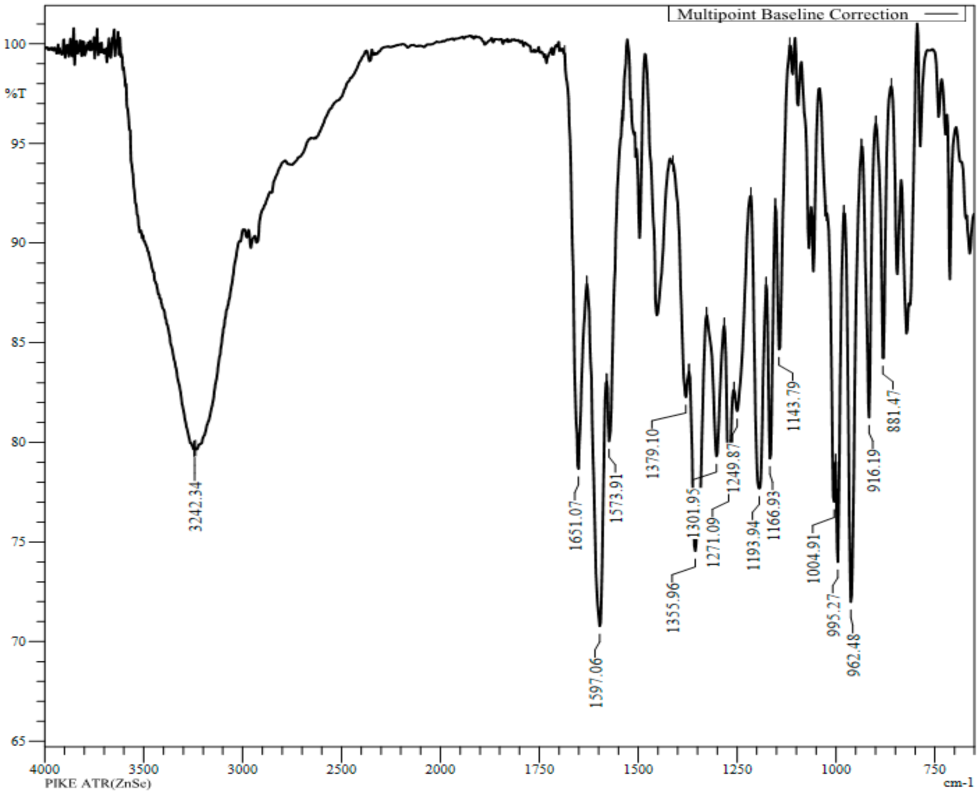

Figure S5. IR spectrum of quercitrin (**2**).

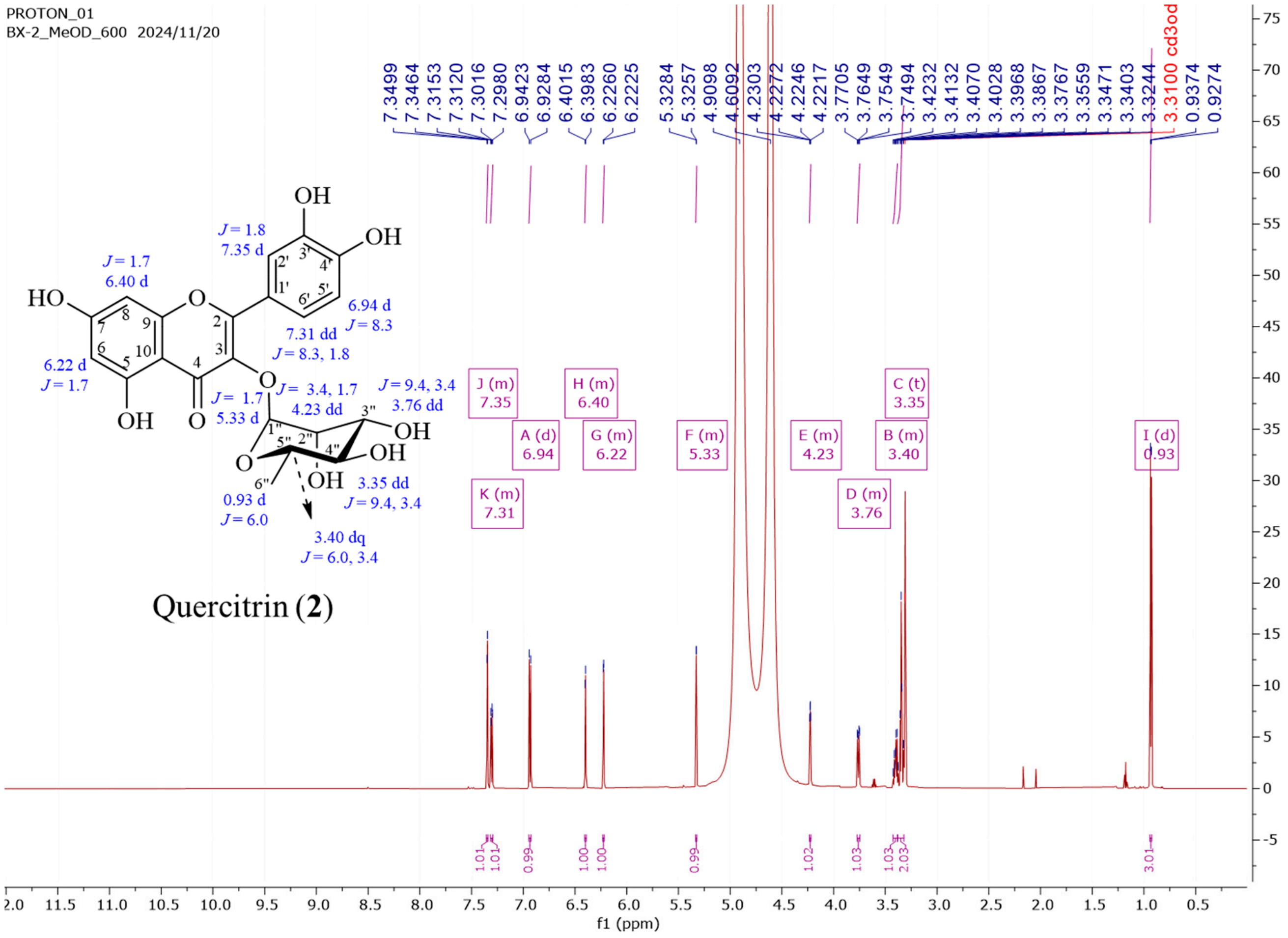

Figure S6. ^{1}H-NMR spectrum of quercitrin (**2**).

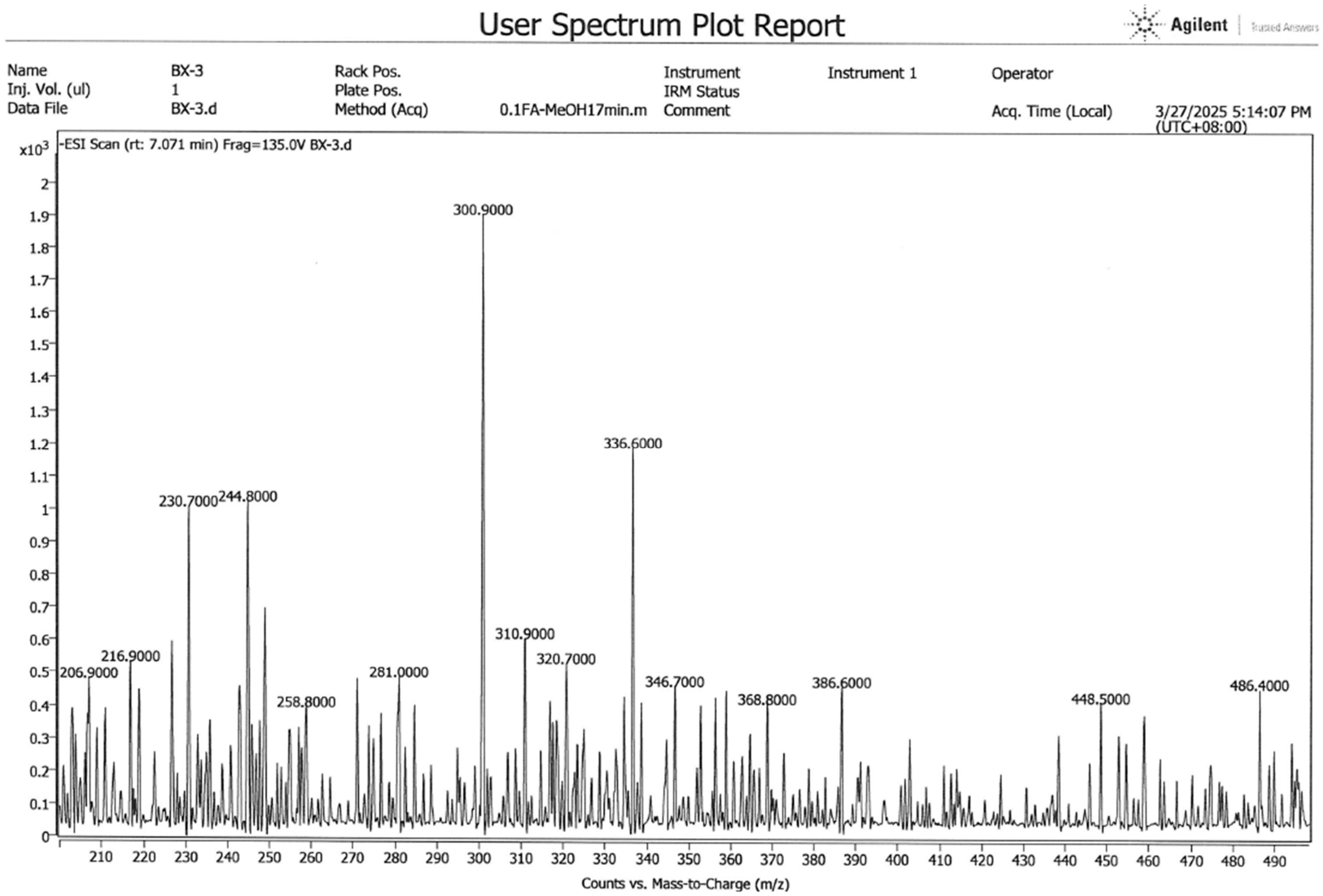

Figure S7. ESI-MS spectrum of quercetin (**3**).

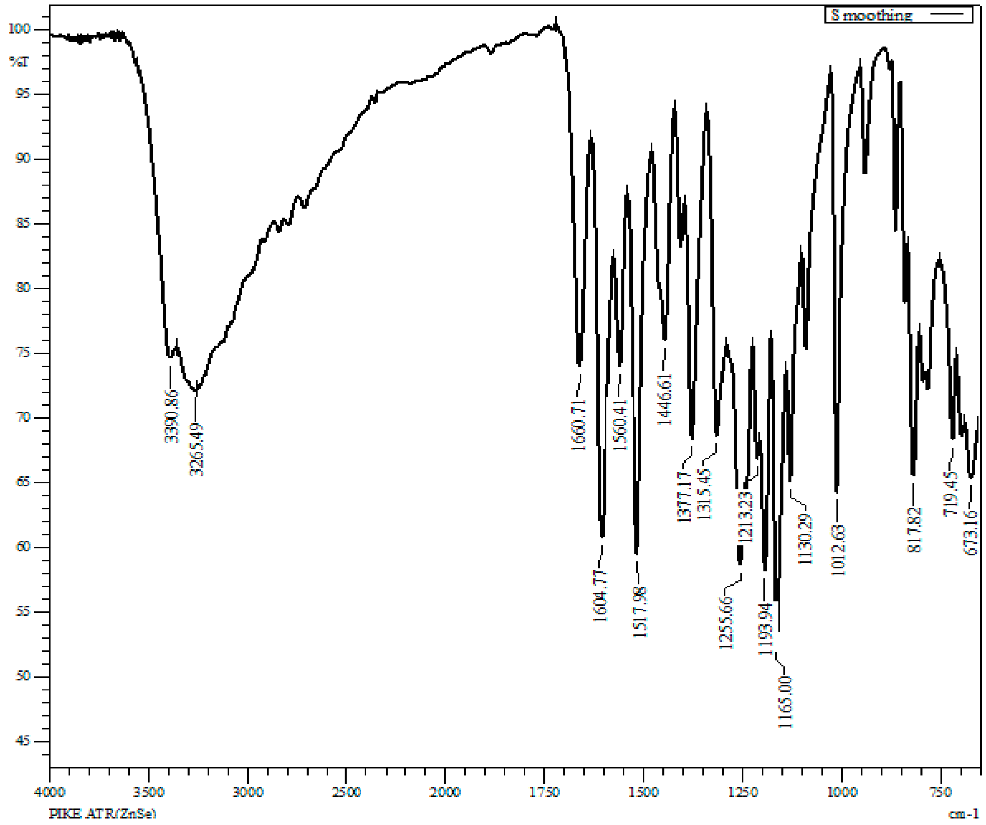

Figure S8. IR spectrum of quercetin (**3**).

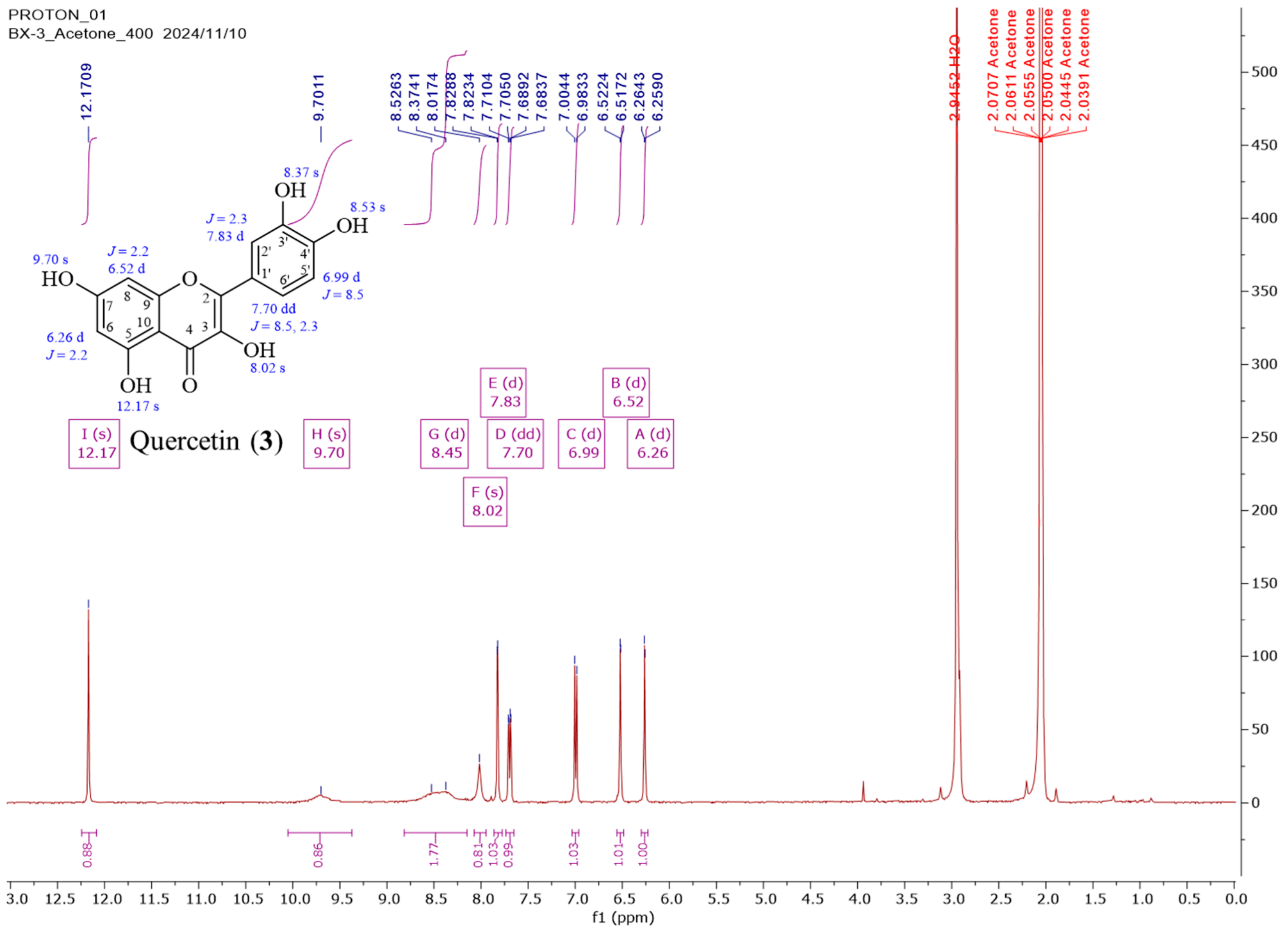

Figure S9. ^{1}H-NMR spectrum of quercetin (**3**).

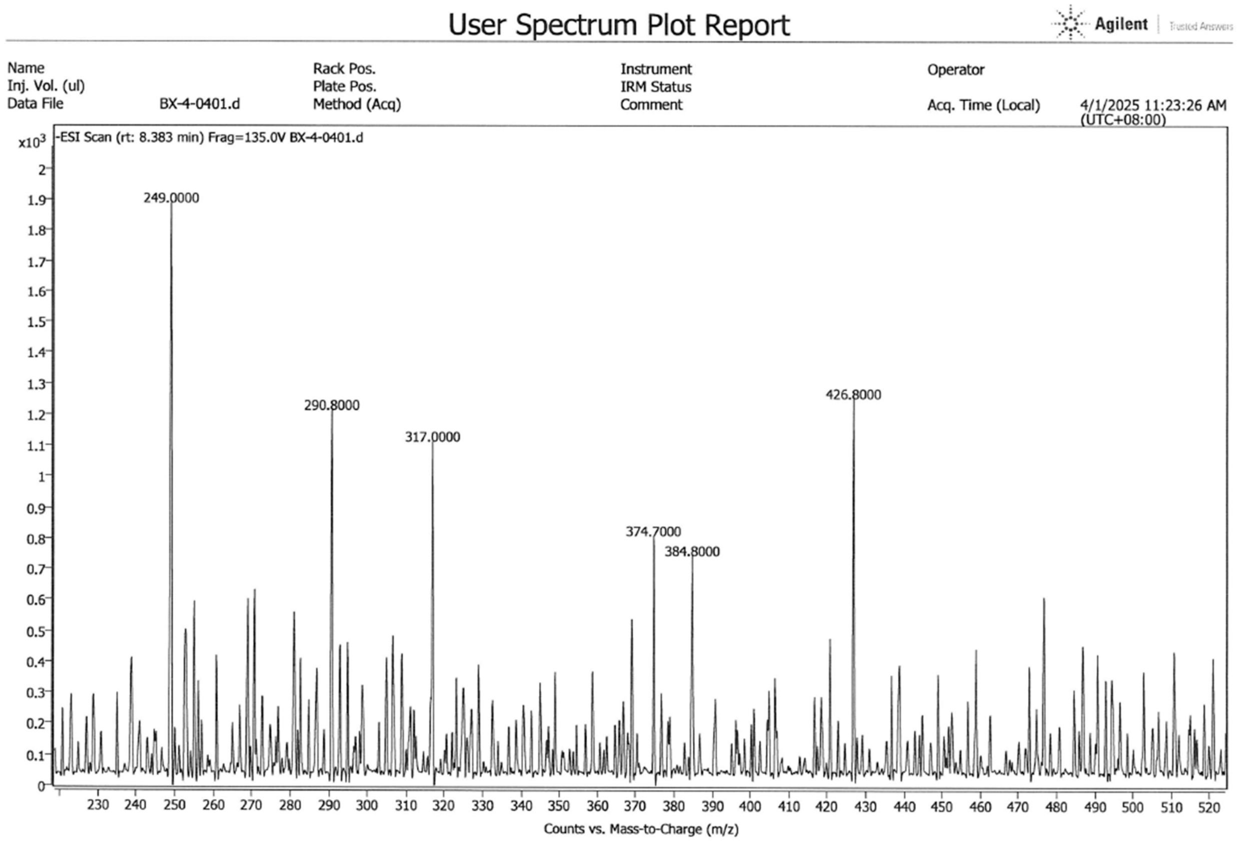

Figure S10. ESI-MS spectrum of myricetin (**4**).

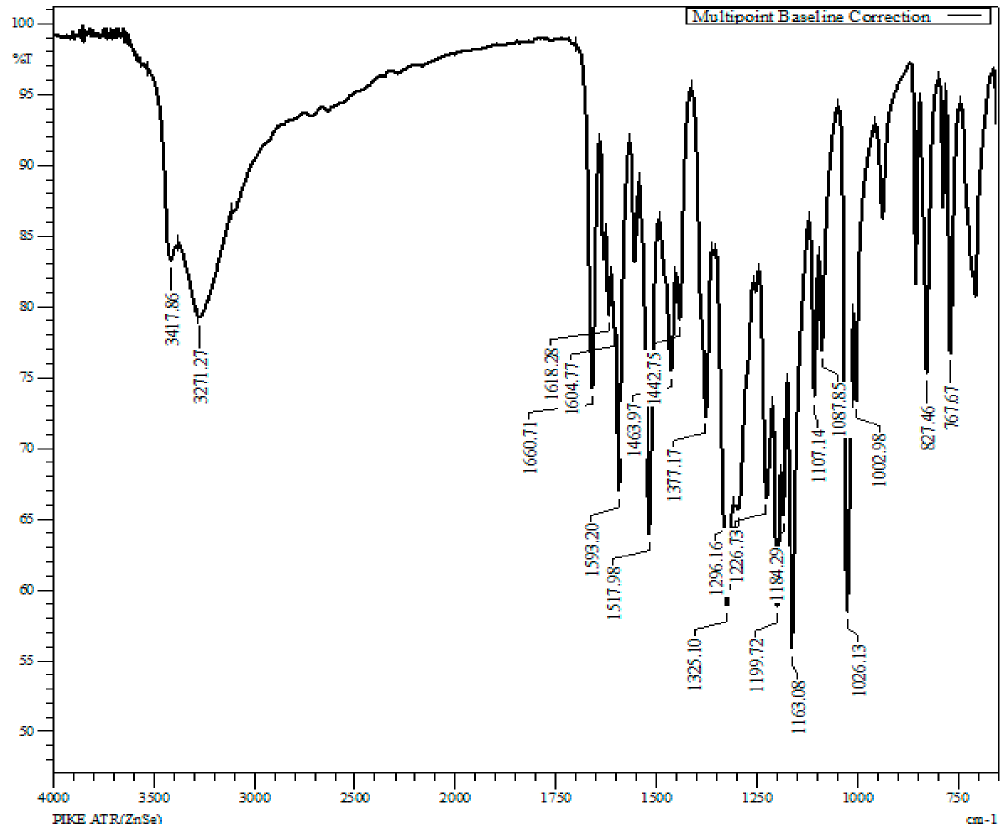

Figure S11. IR spectrum of myricetin (**4**).

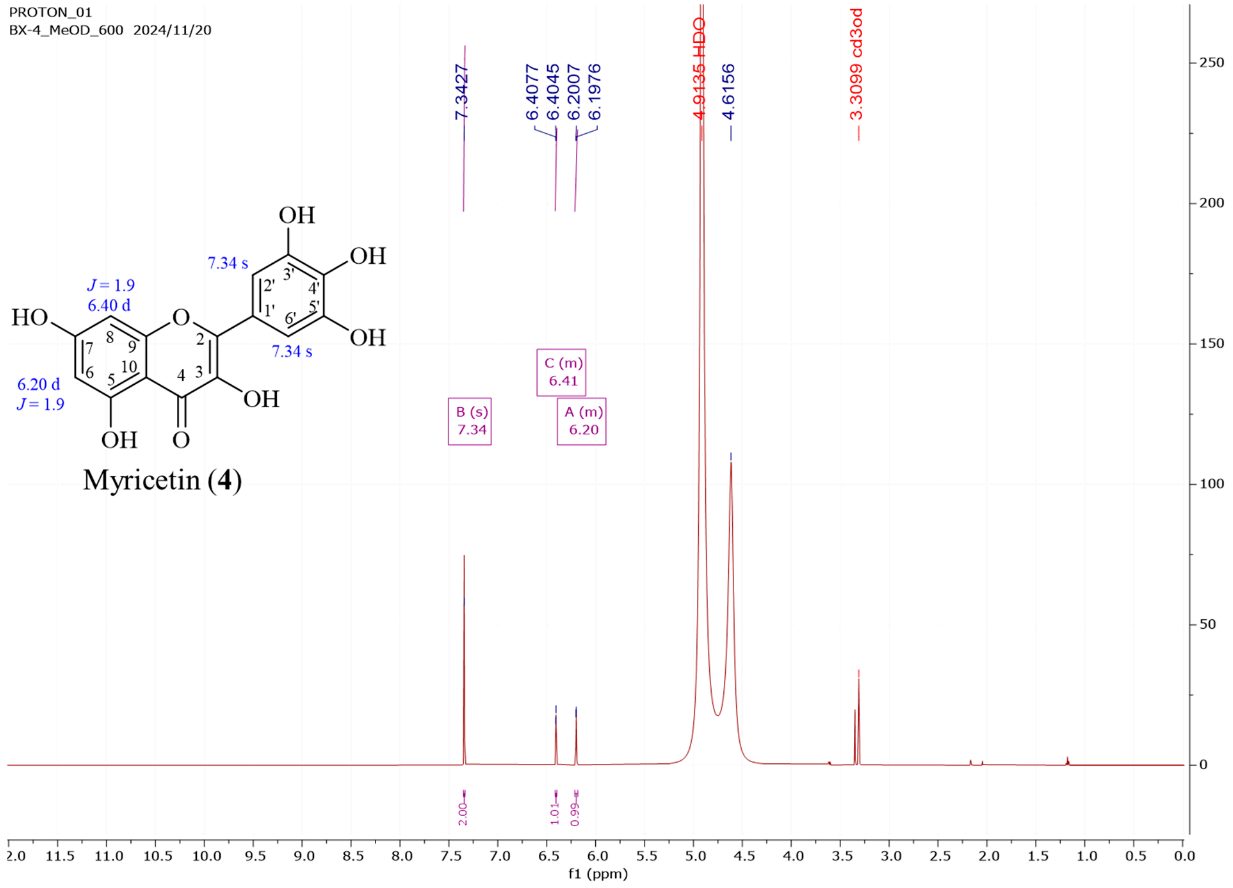

Figure S12. ^{1}H-NMR spectrum of myricetin (**4**).

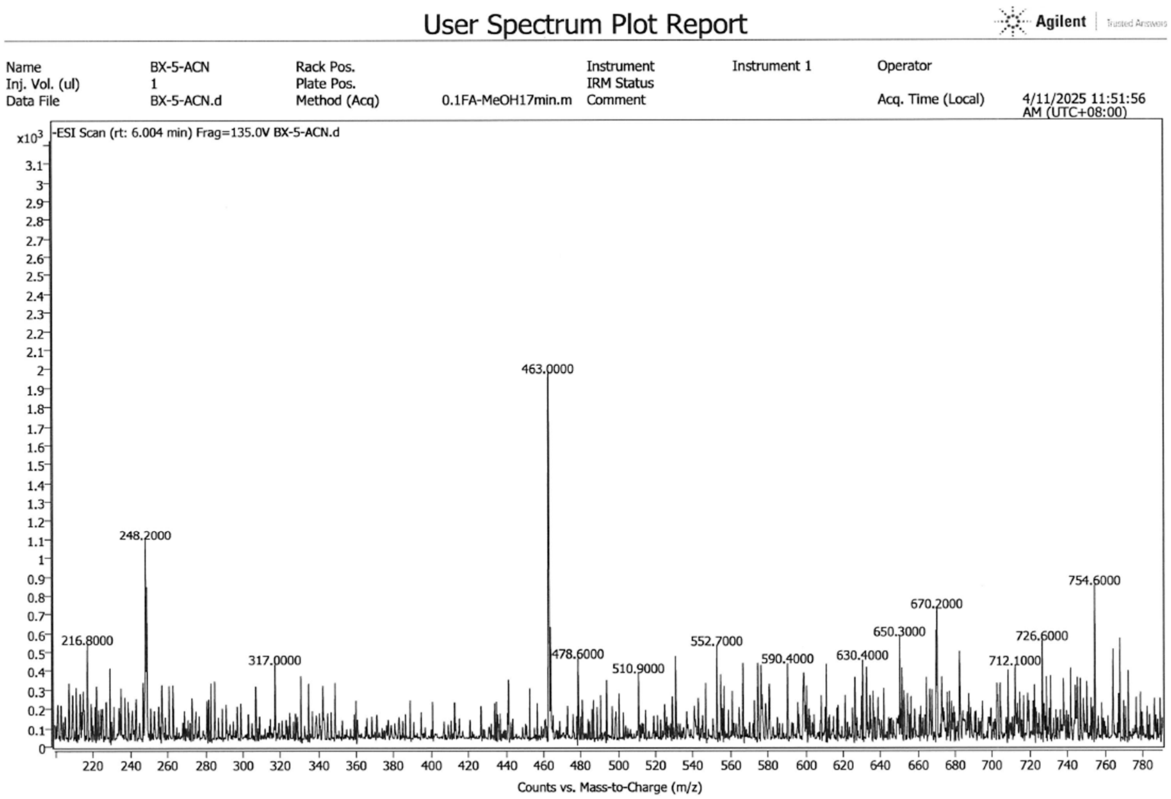

Figure S13. ESI-MS spectrum of myricitrin (**5**).

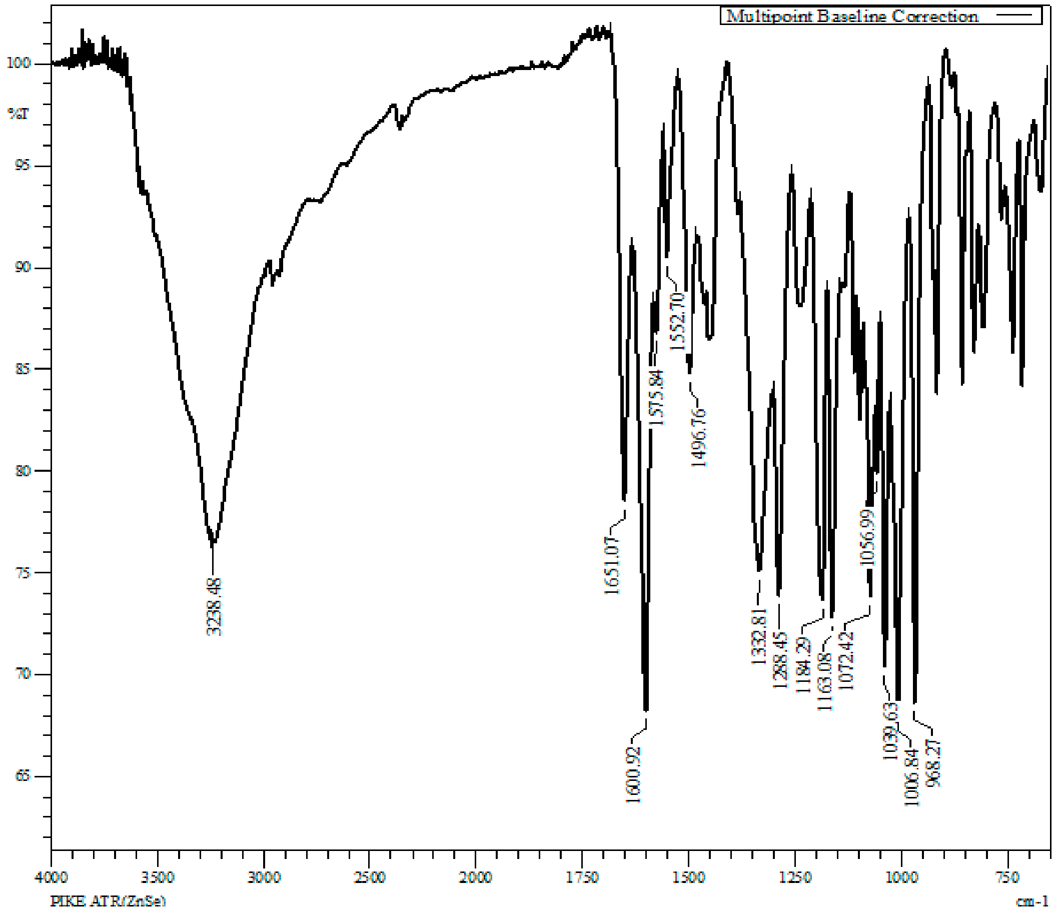

Figure S14. IR spectrum of myricitrin (**5**).

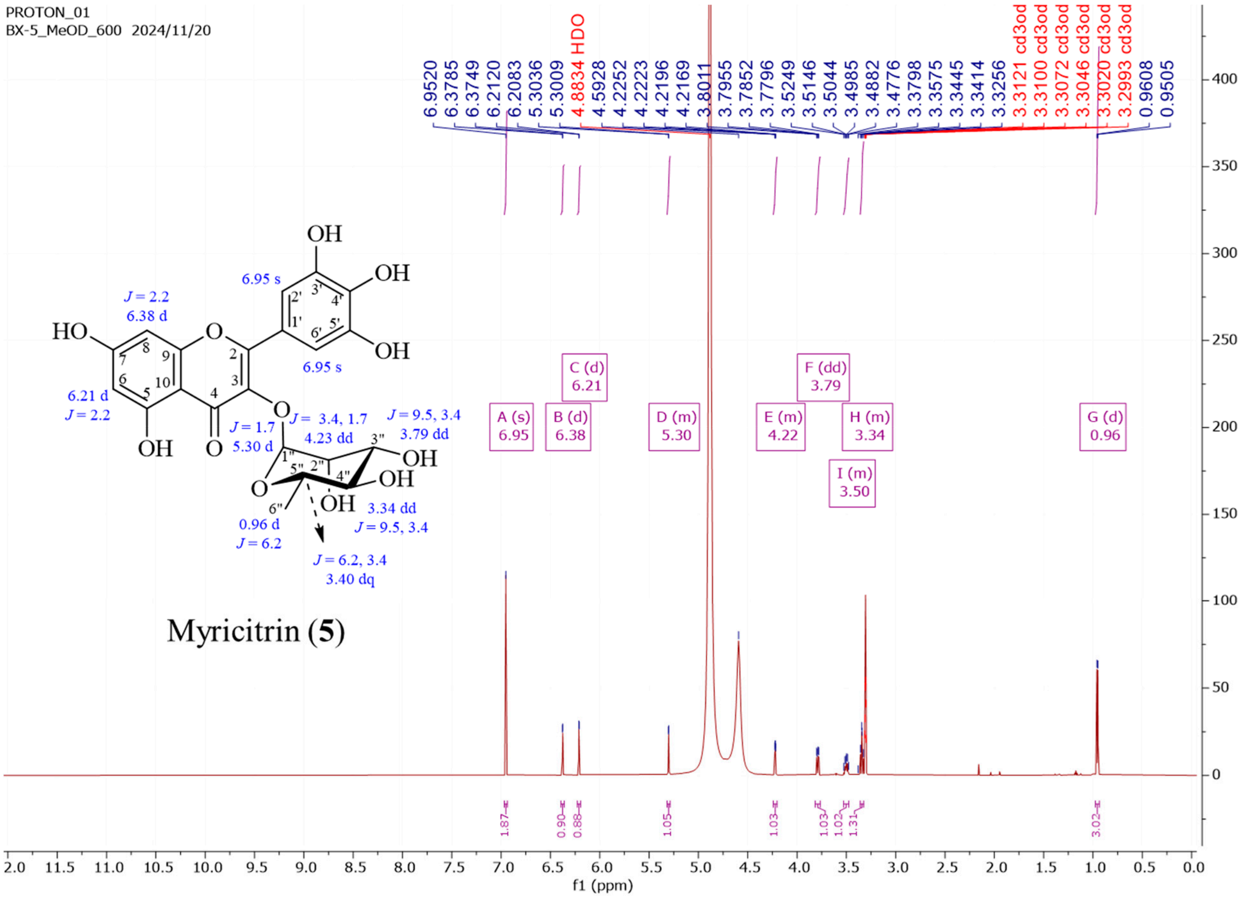

Figure S15. ^{1}H-NMR spectrum of myricitrin (**5**).

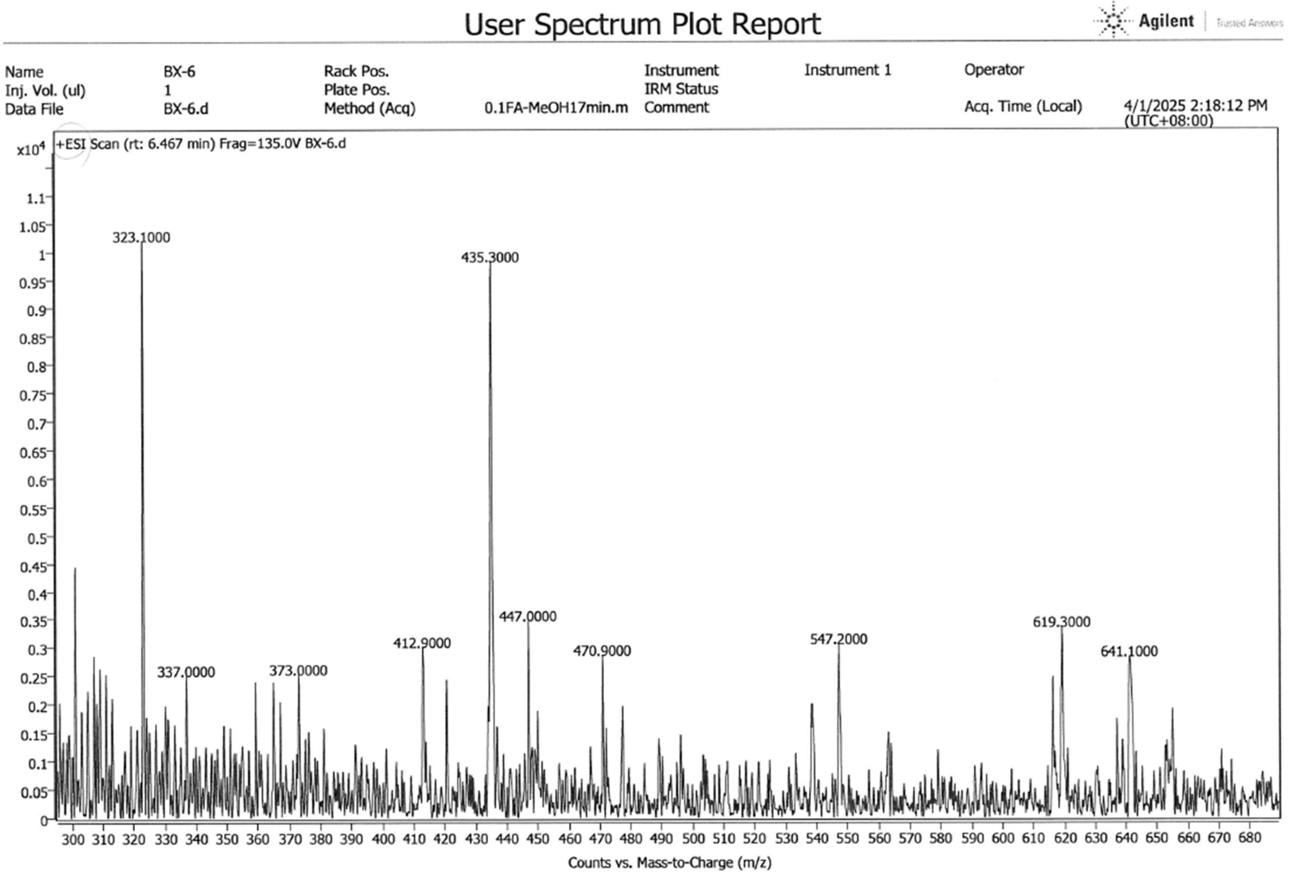

Figure S16. ESI-MS spectrum of avicularin (**6**).

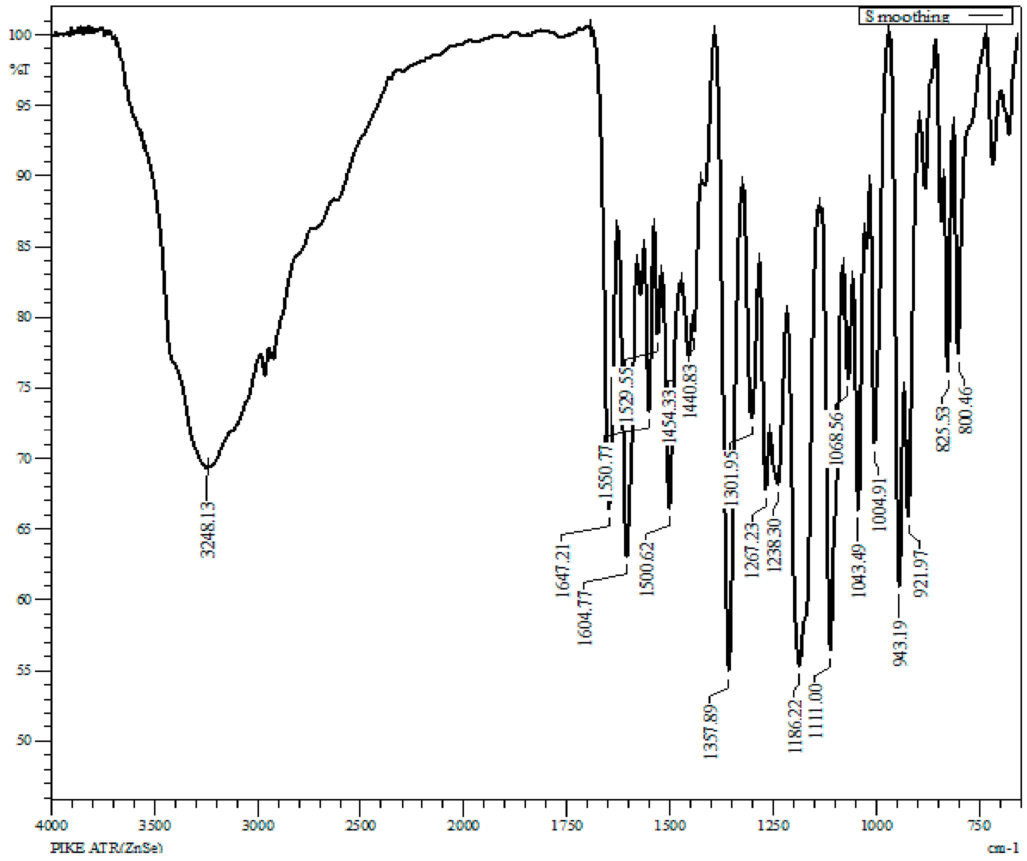

Figure S17. IR spectrum of avicularin (**6**).

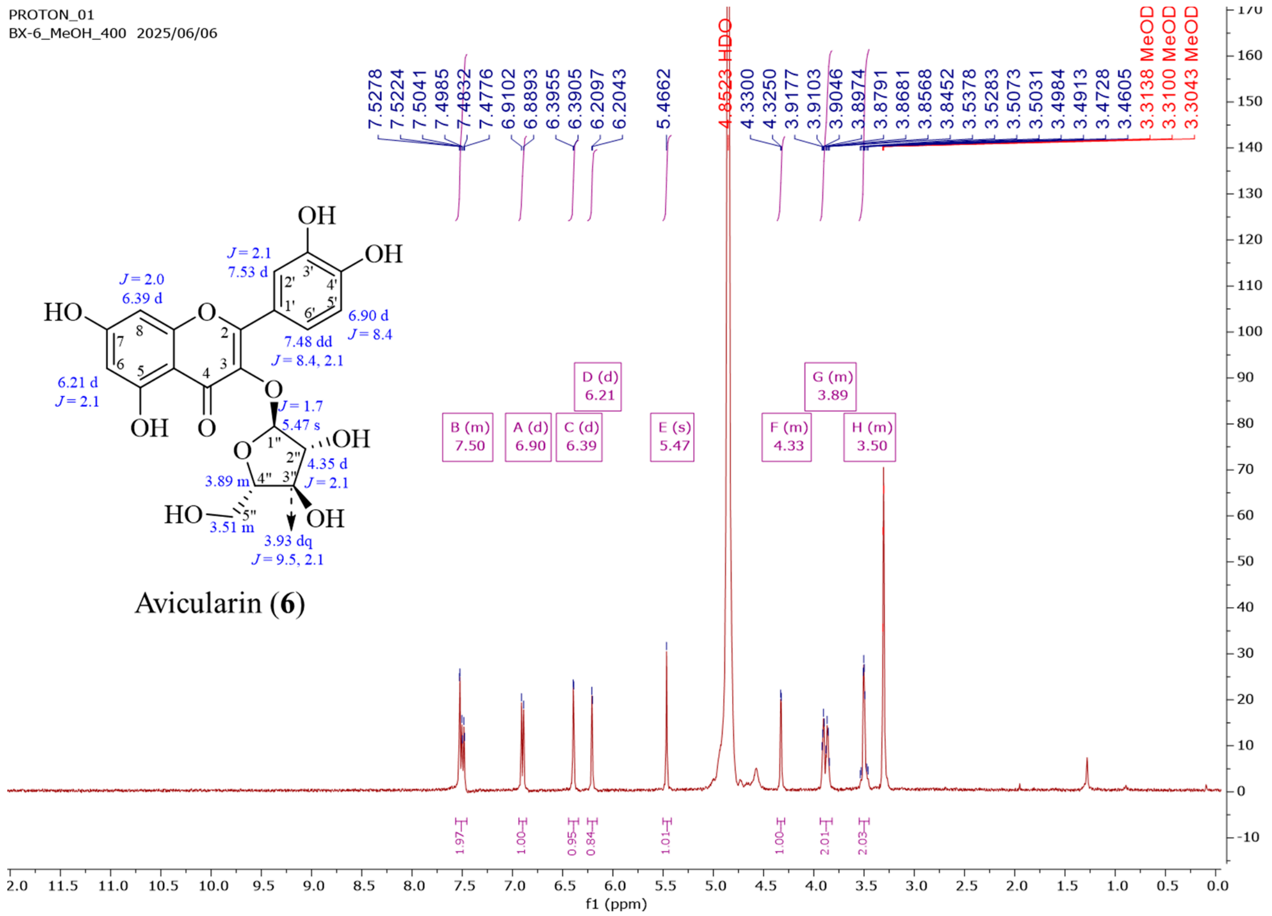

Figure S18. ^{1}H-NMR spectrum of avicularin (**6**).

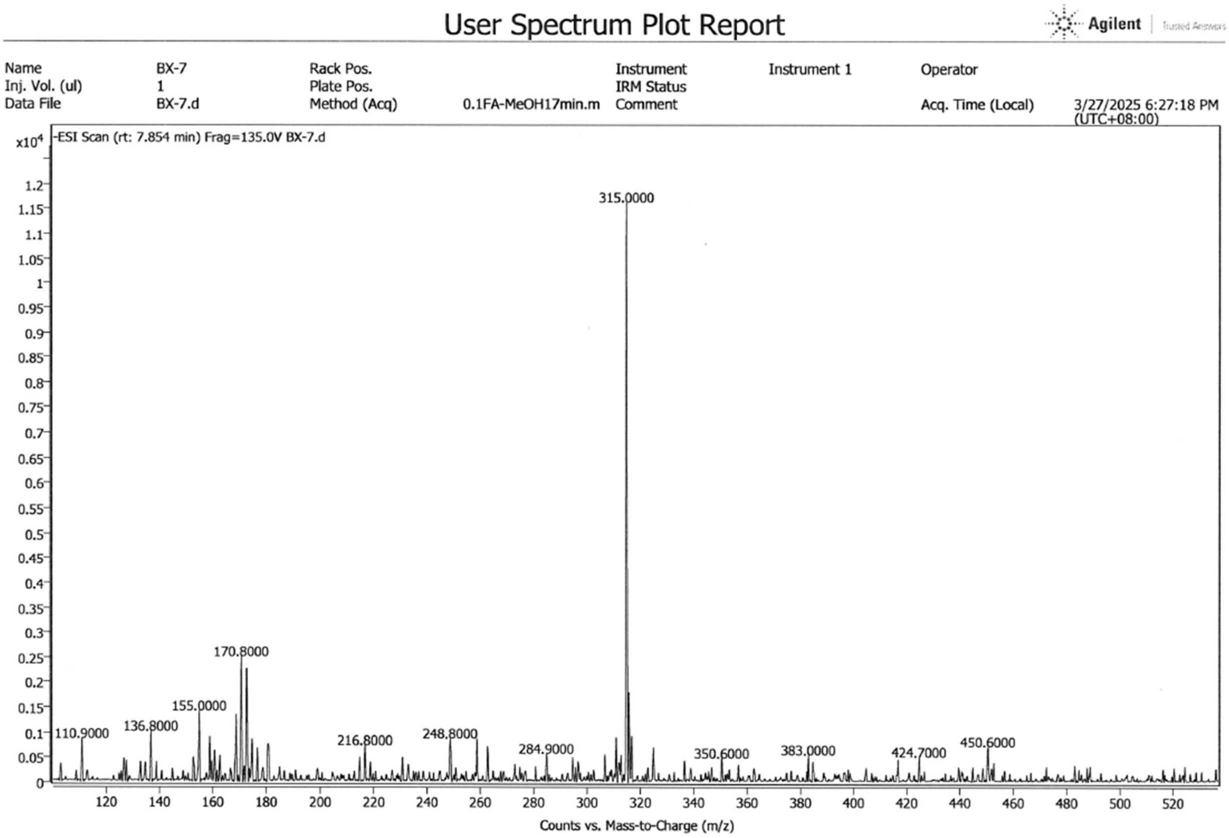

Figure S19. ESI-MS spectrum of isorhamnetin (**7**).

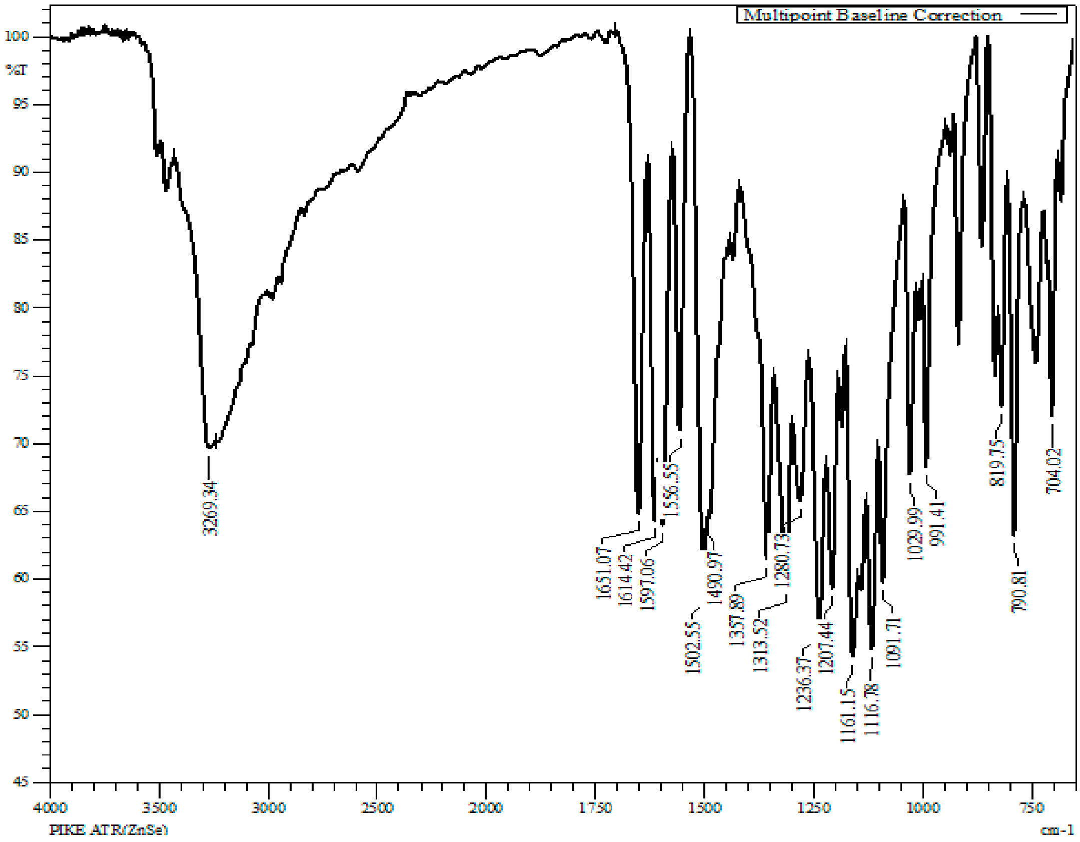

Figure S20. IR spectrum of isorhamnetin (**7**).

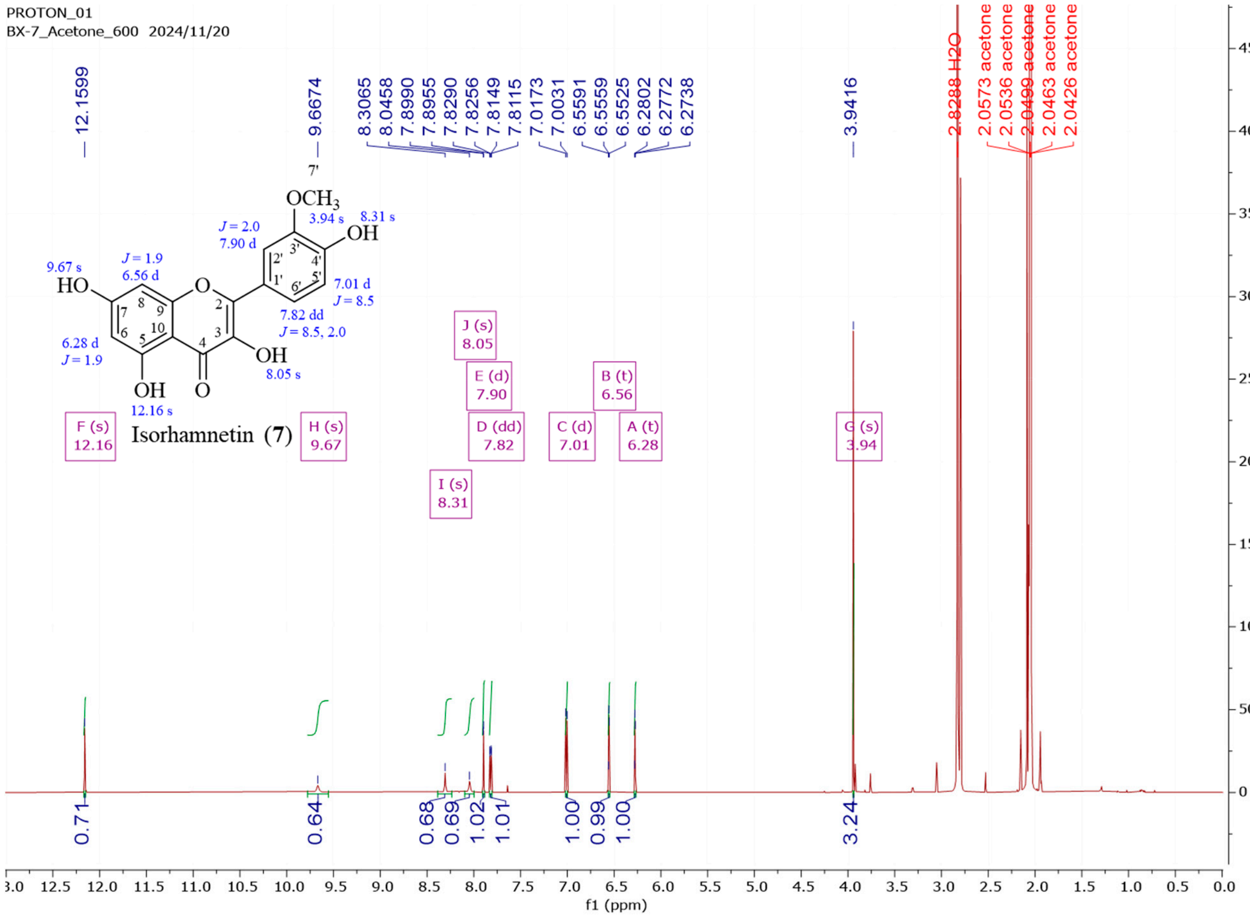

Figure S21. ^{1}H-NMR spectrum of isorhamnetin (**7**).

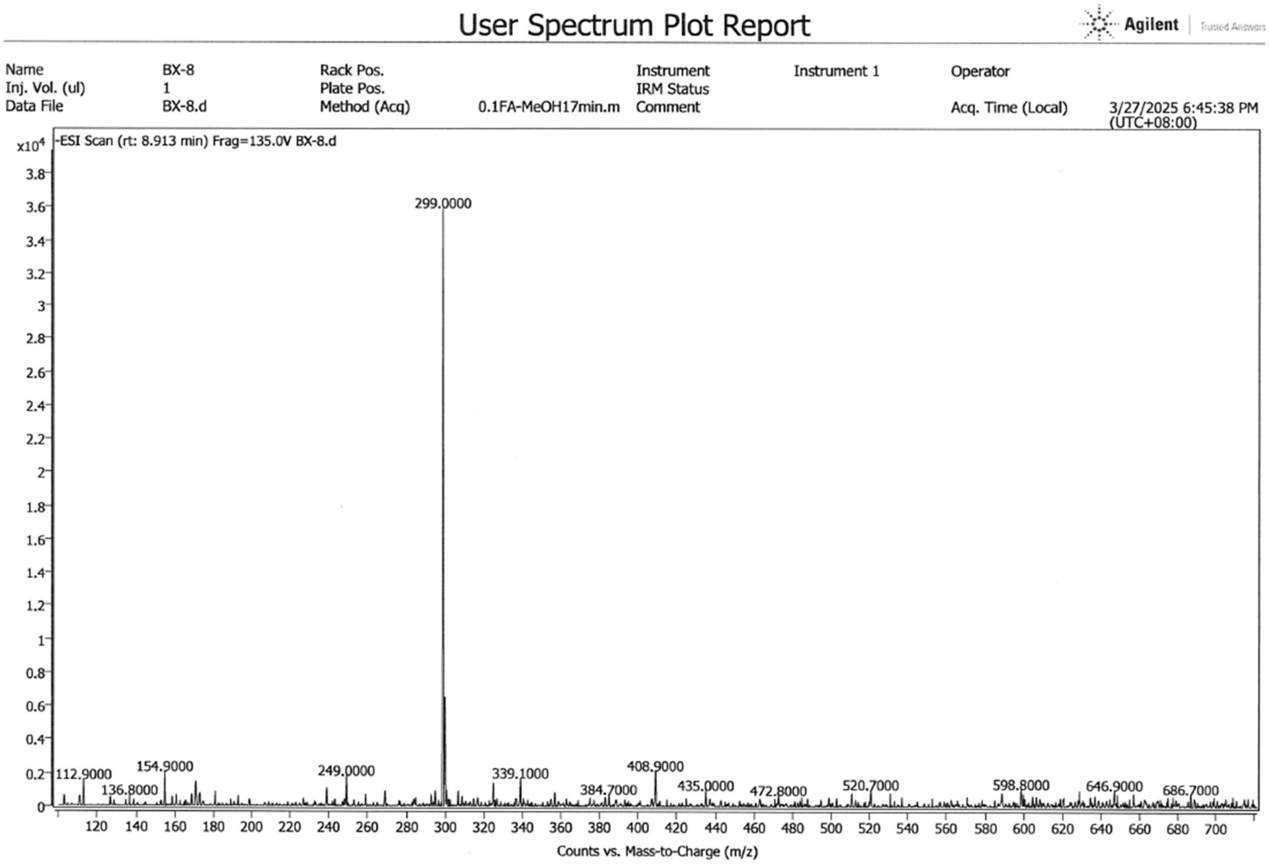

Figure S22. ESI-MS spectrum of kaempferide (**8**).

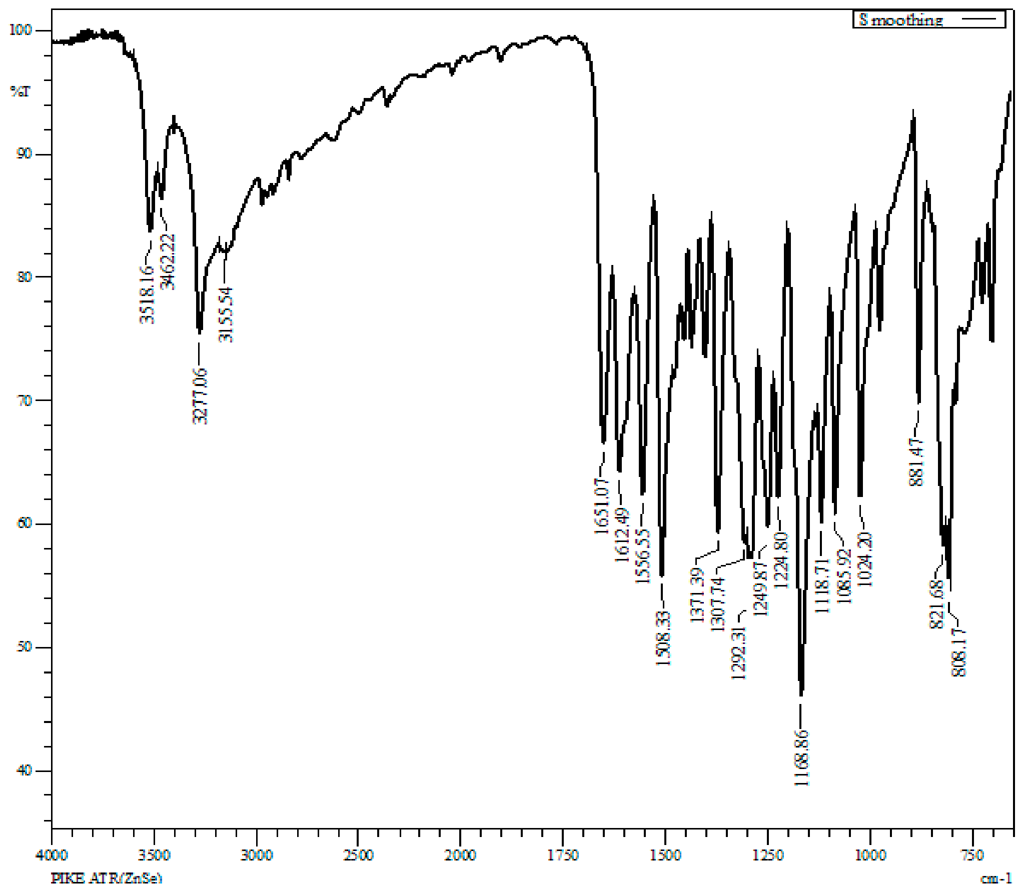

Figure S23. IR spectrum of kaempferide (**8**).

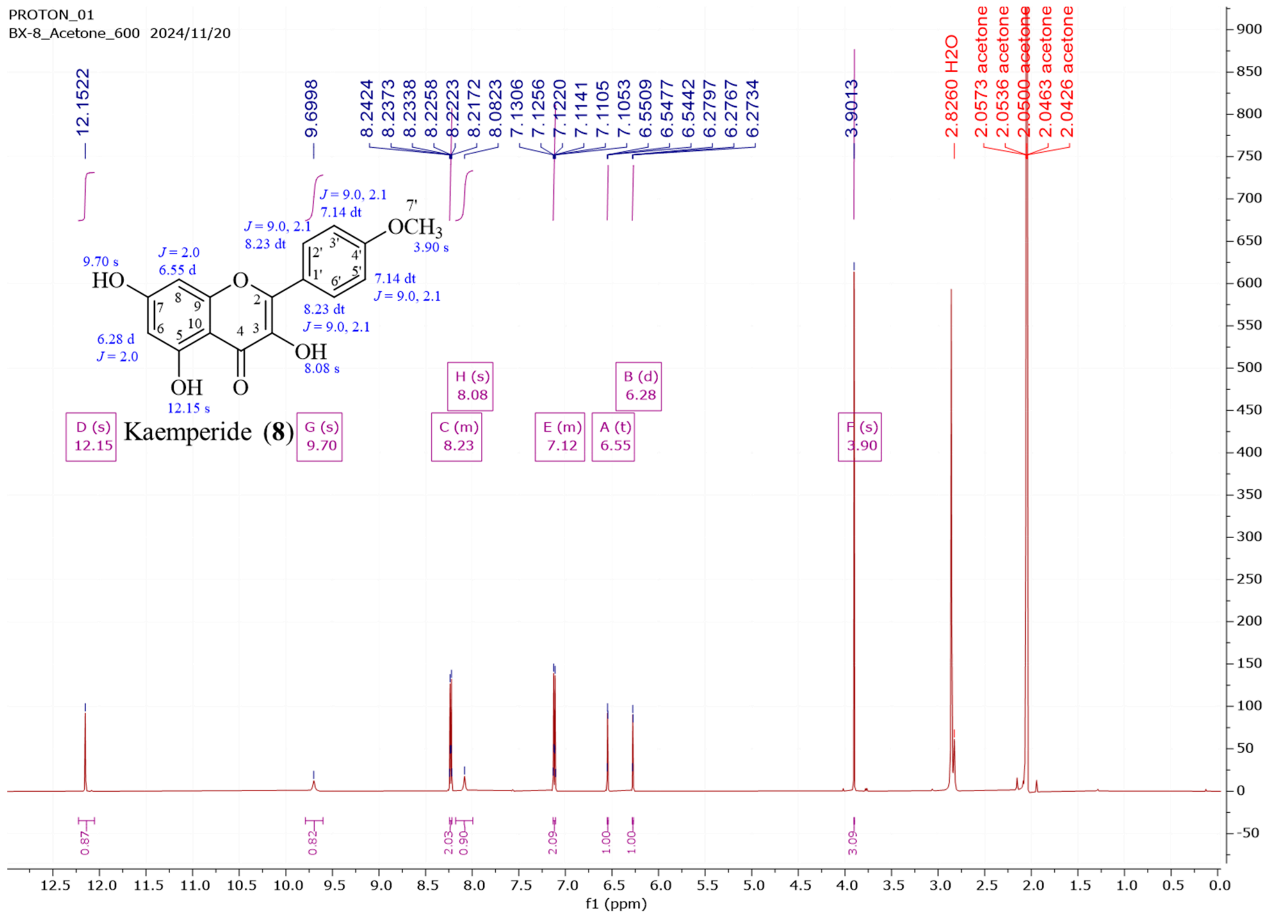

Figure S24. ^{1}H-NMR spectrum of kaempferide (**8**).

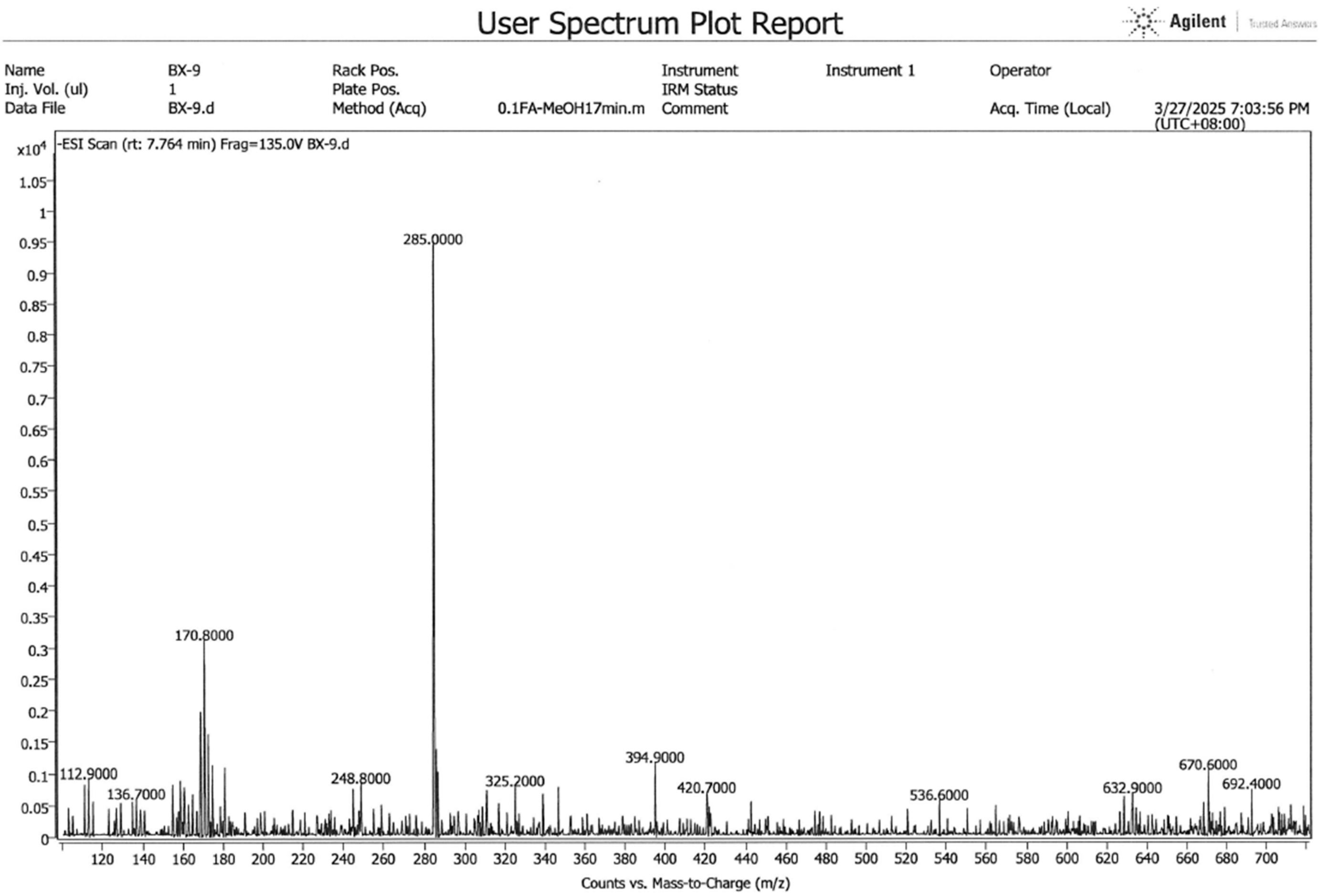

Figure S25. ESI-MS spectrum of kaempferol (**9**).

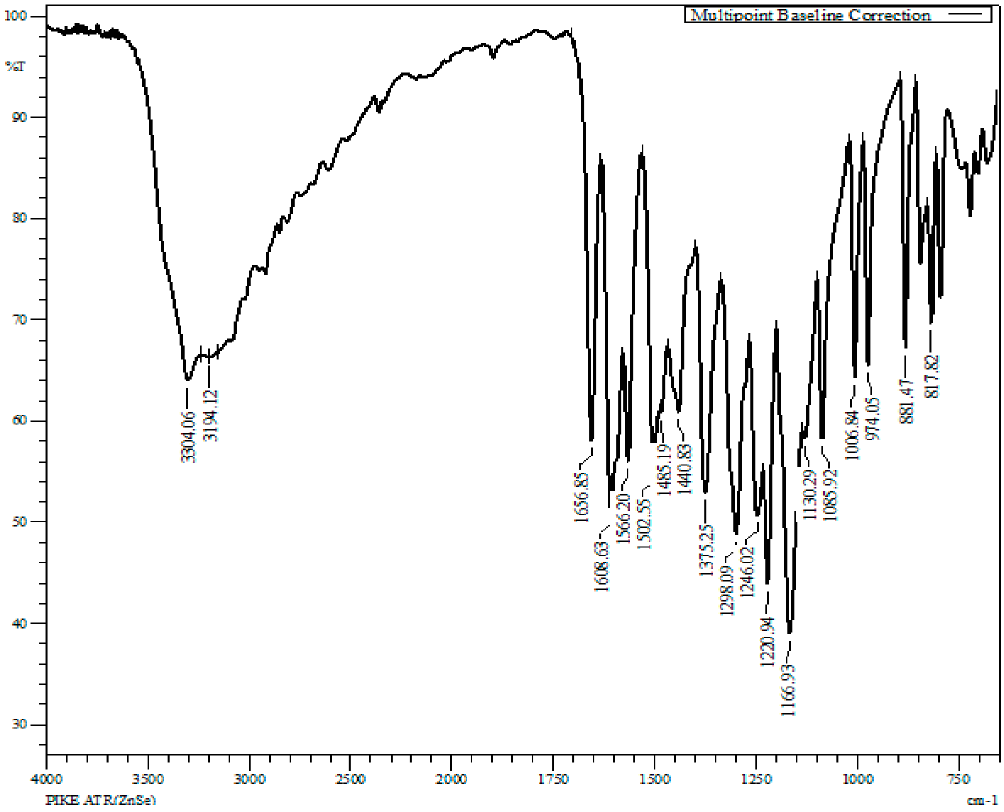

Figure S26. IR spectrum of kaempferol (**9**).

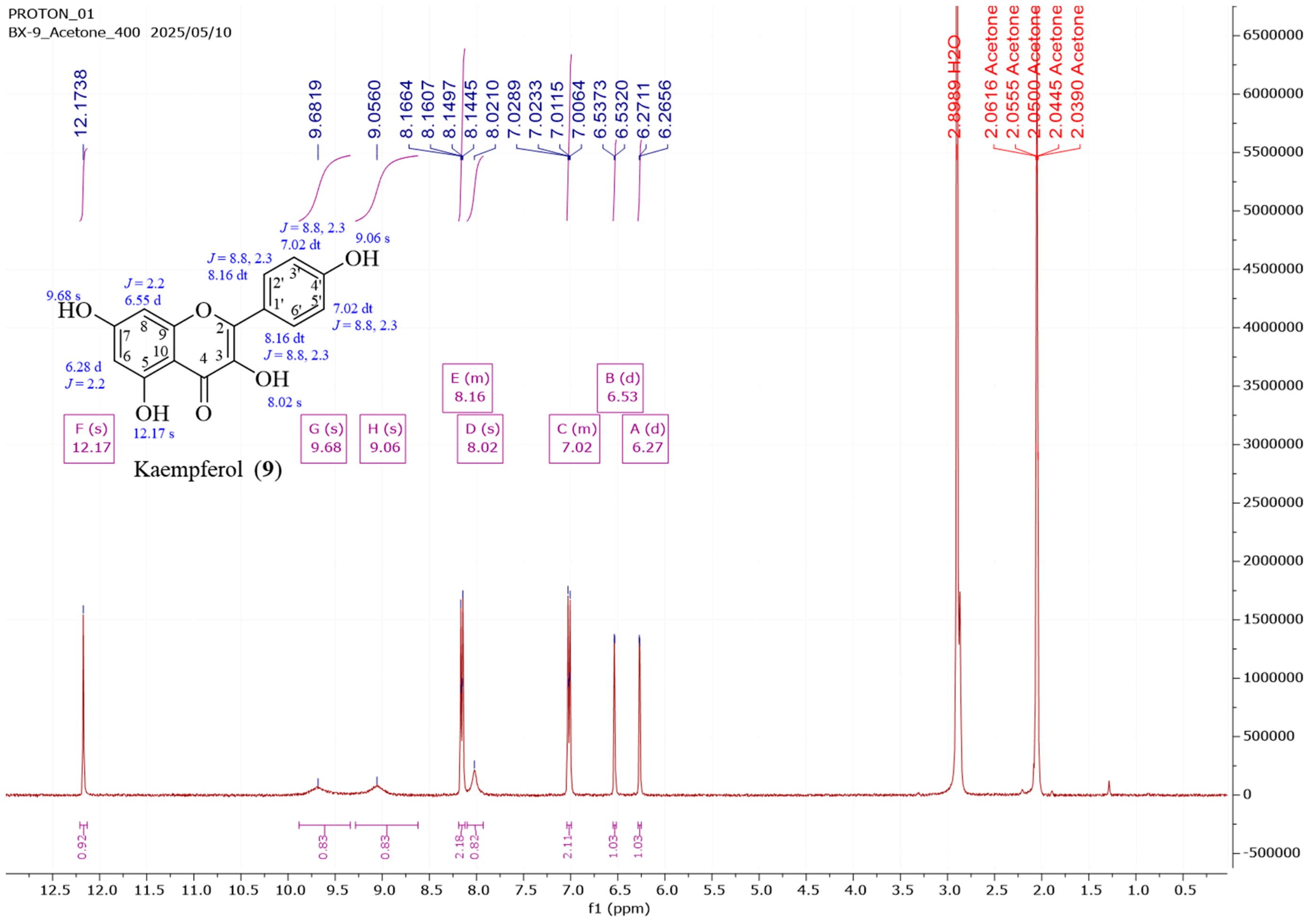

Figure S27. ^{1}H-NMR spectrum of kaempferol (**9**).

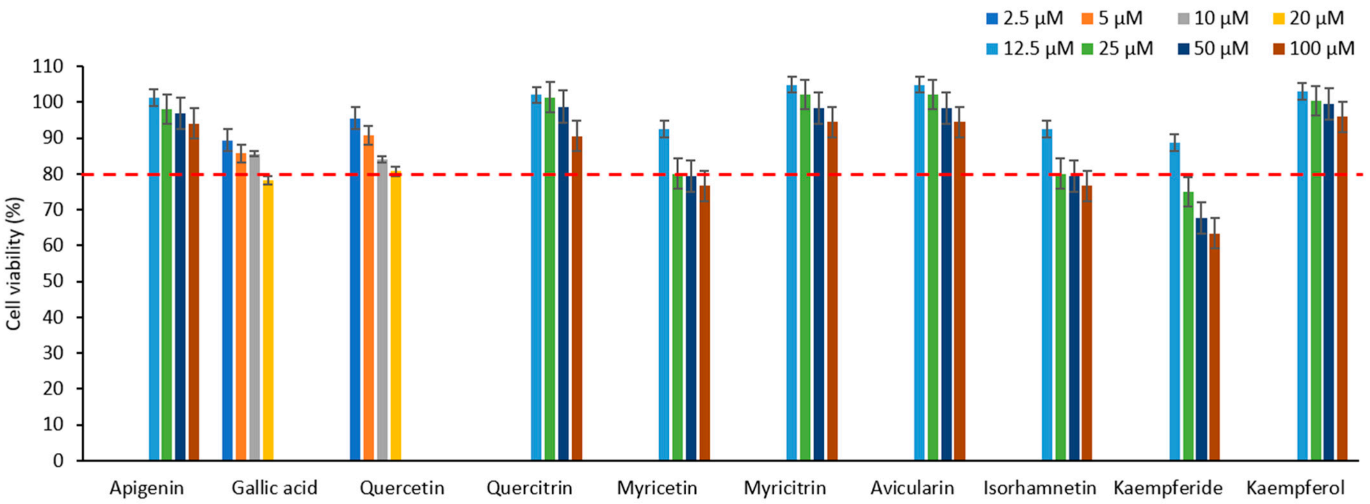

Figure S28. Major compounds of *P. a□iculare* and positive control apigenin on MTT assay using RAW264.7 cell line.

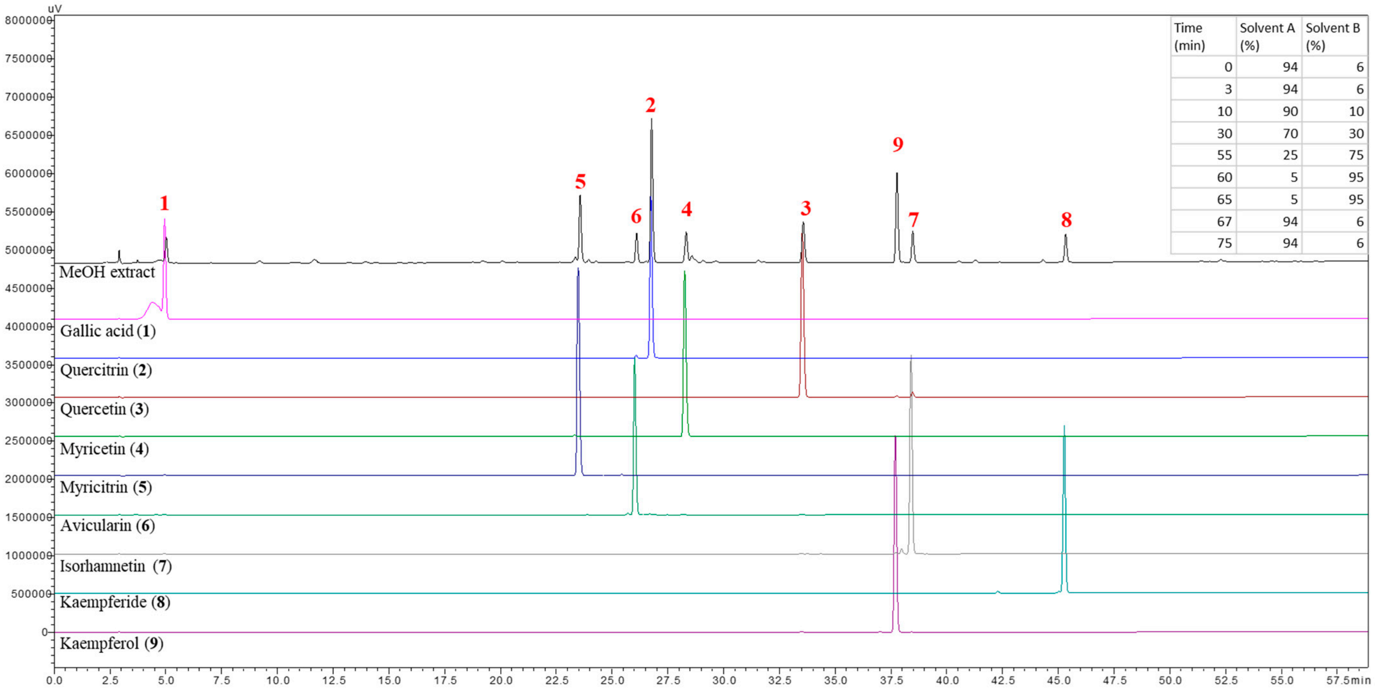

Figure S29. High-performance liquid chromatography (HPLC) profiles of the methanolic extract of *Polygonum a□iculare* aerial parts and the nine isolated reference standards.

HPLC Chromatographic Conditions

Liquid chromatography analysis was performed using a Shimadzu Nexera LC-40 system equipped with an LC-40D X3 pump, a SIL-40C X3 autosampler, and a photodiode array (PDA) detector. Chromatographic separation was achieved on a COSMOSIL 5C18-AR-II reversed-phase column (250 mm × 4.6 mm i.d., 5 µm particle size; Nacalai Tesque) maintained at a constant temperature of 40 °C. The mobile phase consisted of water containing 0.1% formic acid (Solvent A) and acetonitrile containing 0.1% formic acid (Solvent B). The flow rate was maintained at 1.0 mL/min, and the column eluate was monitored at a wavelength of 254 nm. The autosampler cooler was set to 15 °C, and the injection volume was 10 µL with a sampling speed of 5.0 µL/s.

The chromatographic separation was executed using a 75-minute linear gradient elution program as follows: 0–3 min, 6% B; 3–10 min, 6–10% B; 10–30 min, 10–30% B; 30–55 min, 30–75% B; 55–60 min, 75–95% B; 60–65 min, 95% B; 65–67 min, 95–6% B; followed by an 8-minute equilibration period at 6% B (67–75 min).

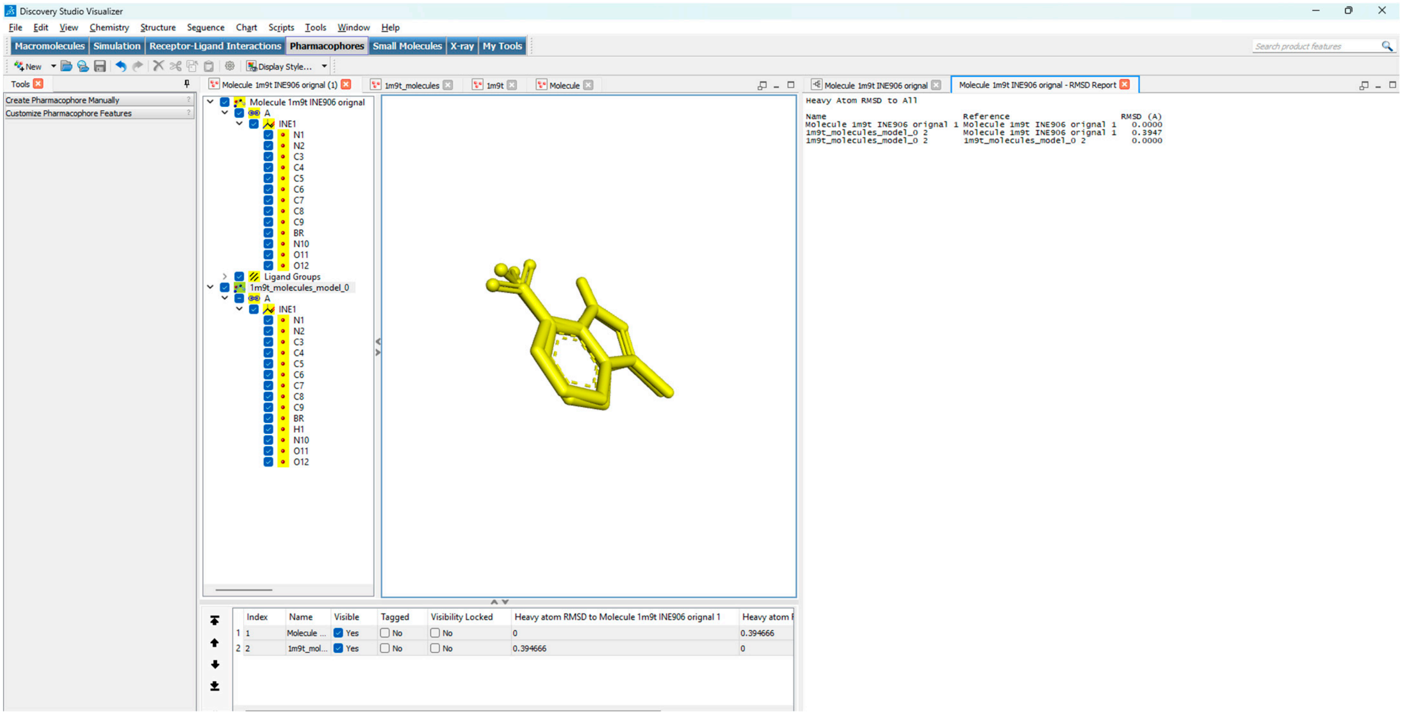

Figure S30. Validation of the molecular docking protocol by re-docking the co-crystallized ligand into the active site of iNOS (1m9t).
